# Comparative dentition in free-living bird nest astigmatan mites

**DOI:** 10.1007/s10493-025-01091-x

**Published:** 2025-12-24

**Authors:** Clive E. Bowman

**Affiliations:** Mathematical Institute, University of Oxford, Oxford, OX2 6GG UK

**Keywords:** Acariformes, Axes, Carpentry saws, Chelicerae, Claws, Competitive exclusion principle, Composite tools, Crushing, Cutting, Ecomorphology, Glaive, Grinding, Gryporial hooking, Nidicolous habitat, Piercing, Scalporial scratching, Sockets, Stripping, Tube cutter, Weaponry

## Abstract

The chelal moveable digit patterns of seventeen free-living astigmatan mite species commonly found in bird nests are decomposed into functional groups using the scoring of observed asperities in order to explain the mites’ coexistence in that commensal community. Type ‘A’ (tearing hook-like) and Type ‘B’ (nibbling) moveable digit morphotypes are found and the default durophagous ‘Bauplan‘ of the mastication surface is described in great detail. The angles of mastication surface features are important and suggest that asperities are adapted to handle aggregate material. The mites’ cutting and crushing moveable digit is asymmetrically designed anterior$\rightarrow$posterior. Stochastically the mastication surface of the moveable digits of bird nest astigmatans map between International Roughness Grade Numbers N5 to N7 suitable for fine grinding. Various gripping and bladed adaptations for browsing, scooping and scraping are highlighted. Miniaturisation reduces the opportunity for trophic design space differentiation. Examples of underbite suitable for levering up material are found. Blunt and shallow features (i.e., ‘pads’) may be suitable for contact with and the gripping of compliant or wetted material. The function of digit tips is discussed. *Aleuroglyphus ovatus* is a crusher. *Chortoglyphus arcuatus* is a chewer. Some glycyphagid moveable digits approximate saw designs. *Acarus farris* is highly variable around a plesiomorphic form. The differentiated surface of *Tyrophagus putrescentiae* proximal to the condyle adapted for specialised gleaning is confirmed. Some species have a derived form proximally with a ‘latch’ comprised of an almost right angled zenith tooth and a very strong ‘pocketed’ gullet just before the end of the mastication surface for ‘snapping’ material. The detailed pattern of gullets is more nuanced across species than the pattern for teeth. *Rhizoglyphus robini* showed adaptations to deal with plant material. *Dermatophagoides pteronyssinus* is designed unusually. Nematophagy is discussed. Of the taxa reviewed, it only remains unclear how *Tyrophagus palmarum* and *Tyrophagus similis* morphologically avoid direct trophic competition in this habitat.

## Introduction

The background to this review is OConnor (1982) who said: “...As a consequence of their small size, mites (Acari) are able to exploit specific habitats and ecological niches unavailable to larger arthropods. Among these habitats are many that are specifically created by other animals, both invertebrates and vertebrates. Such habitats include nests, burrows, galleries, food storages, fungus gardens, uneaten prey remains, refuse piles, dung and carrion, as well as a multitude of microhabitats on and in the bodies of the host animals themselves. The ability to exploit such niches is the result of numerous adaptations, many of which have transformed an ancestral predatory animal, capable of ingesting only liquid food, into a variety of predatory and non-predatory forms whose food sources are extremely diverse, particularly when compared to other arachnid groups. Although such ecological associations with other animal groups have evolved in a larger number of acarine groups, the cohort Astigmata (Order Acariformes, Suborder Sarcoptiformes) with 69 families and 785 genera represents by far the largest adaptive radiation of mites into symbiotic associations. Other acarine lineages include relatively diverse radiations in association with both arthropods and vertebrates, but none approach the diversity of the Astigmata....”

Natural tree cavities and nest boxes are a haven for arthropods McComb and Noble (1982). Indeed, “... Bird nests amaze us with a diversity of forms, occupied places, structures and building materials used, and provide various abiotic and biotic conditions for invertebrates....” Laska et al. (2023). They are a model system for a “... better knowledge of non-parasitic relationships involving... mites and other animals to be inferred...” Fain et al. (1993). They are unstable microhabitats (merocenoses) and the biology of nest-inhabiting acarines is often modified by adaptations for living in such habitats, particularly for phoresy (Napierała and Błoszyk 2013). The bulk of bird nest dwelling arthropods are saprophilous Krištofík et al. (2013).

Species can be partitioned into discrete niches within a habitat in many ways over time and space. Undoubtedly, “.... nest characteristics... influence the composition and abundance of mite fauna...” Laska et al. (2023). However, how, and therefore on what, an animal feeds is very varied (https://en.wikipedia.org/wiki/List_of_feeding_behaviours). As such it is a key factor in avoiding direct competition for the same niche leading to species elimination or exclusion (Begon and Mortimer 1981). Mandibular size and shape is important in allowing chiropterans to occupy distinct ecological niches (Crampton et al. 2024). Bowman (2023a) has already shown the importance of moveable digit mastication length (aka ‘tooth row’ $x_{i_{e}}$), and thus gape as in fishes Mihalitis and Bellwood (2017), in affecting the likely maximum foodstuff size and resource use by UK beehive living astigmatans. Bowman (2024a) went on to examine the apparent crushing, sawing, stabbing, slicing, cutting and hooking surfaces using whole profile derived measures in these insect nidicolous mites.

**Fig. 1:**
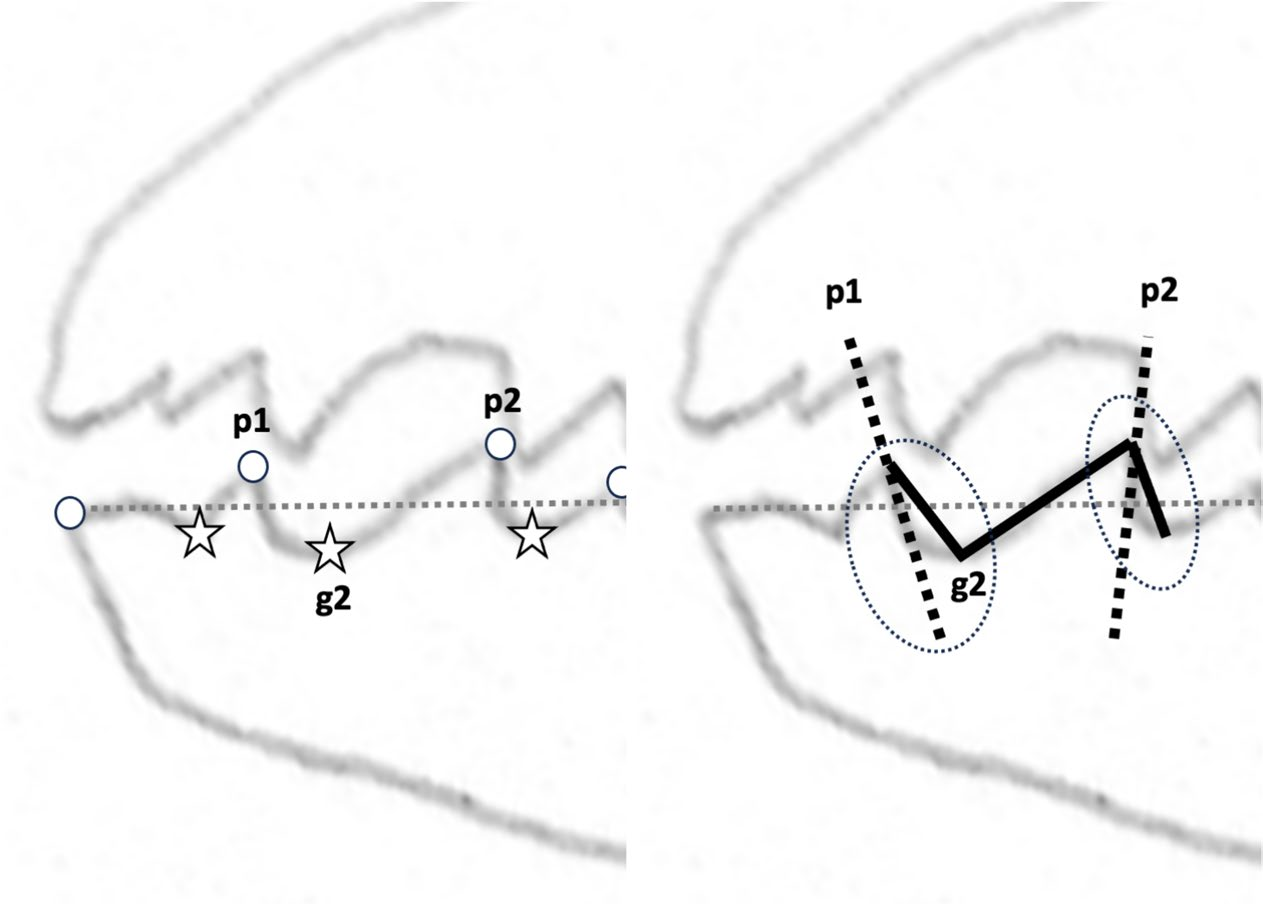
Location of *actual* peaks (open dots, p*i*) and gullets (stars, g*i*) on anterior section of the chelal moveable digit profile in *Tyrophagus putrescentiae* (amended from Fig. 12 in Bowman 2024a with permission, Creative Commons Attribution 4.0 International License http://creativecommons.org/licenses/by/4.0/). Left: Marked up profile. Zeroth peak is at moveable digit tip (= origin). Star posterior of this is first gullet (g1). Right: Solid lines showing how nadir angle for gullet g2 and zenith angle for peak p2 is estimated. Note in circled areas how the initial curved slope of the asperity’s profile (dashed lines) is lost by this *continuous* linear approximation

This second paper investigating and reviewing the biomechanical design of free-living astigmatans found in bird nests will (like Bowman 2024a) focus on the detailed dentition of the moveable digit as a tool. In contrast to Bowman (2025b), it will use a variety of measured angular measures of the actual observed asperities (Fig. 1). This is in order to resolve outstanding questions from that previous whole profile study and to set up a methodology possibly suitable for use on other chelate-dentate mites. This review will draw upon publicly available historical engineering material regarding saws (http://www.backsaw.net/index.php/2-uncategorised/4-saw-literature-on-line) and weapons (https://www.thearma.org and Turner 2002) which should be consulted for more detail on such tools.

Arthropods reach back to the Ediacaran (Giribet and Edgecombe 2019) and actinotrichid mites have been around since the Devonian period (Dunlop 2010) 419.2 to 358.9 million years ago. Many small interrelated changes feature in animal evolution (even for the origin of higher taxa, Kemp 2016). Structures arising from these need to retain their practicality. Accordingly ‘echoes’ of the same ’fingerprint’ of species differentiation should be present at many scales whether in physiology or structures (for example: 3D bone geometric measures can predict muscle mass well, Cassini and Vizcaíno 2012; a combination of craniofacial features predicts lifestyle in kangaroos and wallabies, Mitchell and Wroe 2019). Any particular species is adapted for its life as a whole, so there may be many different designs that as *composites* favour saprophagy. In order to resolve “...the tension between simplicity and complexity...”, such designs will be analytically dissected (i.e., ‘un-made’) and reassembled (i.e., ’re-made’) in this investigation honouring the legacy of the great anatomist Andreas Vesalius.

Biomechanical analyses will follow the approaches used on other animals (e.g., Nabavizadeh 2023). Differentiation in dentition along the whole digit will be examined in this review and jointly interpreted in terms of macro-scale tools (Bowman 2025b). Just like in ants (https://www.antwiki.org/wiki/Morphological_and_Functional_Diversity_of_Ant_Mandibles), astigmatans may use their mouthparts for the manipulation of objects, food processing, hunting and defence. Accordingly much use will be made therefore of comparisons to macroscopic tools using historical catalogues (many of which can be found at https://www.blackburntools.com/articles/rose-tools-catalog-archives/index.html). These highlight the diversity of composite tool form and engineering function invented by man. Since saprophagous astigmatans must be challenged to consume nidicolous material approximating cheese, fresh meat, fish, processed foods (like sausage, ham, bacon etc.,) not just resources of a fungal or vegetable origin, consideration will also be made of industrial designs for foodstuff slicing (and their mechanics Bremer and Matthiesen 2020). The abilities needed for possible adventitious feeding upon nematodes will also be discussed.

## Rationale for studied measures

Multiplicity of design will be true for saprophagous mites (just as historically, there are many ways to be a small carnivore (https://www.researchgate.net/publication/341940273_Multiple_ways_to_be_a_small_carnivore_in_the_Late_Miocene).The maxim behind this review can then be mapped to a quote (here translated from Russian) taken from Lev Nikolayevitch Tolstoy, *Anna Karenina* (1875-1877) Part 1 Chapter 1:

“All happy families resemble one another, each unhappy family is unhappy in its own way”.

That is, mites which may trophically compete directly will be designed the same mechanically. However, those that can successfully co-exist, each will be designed distinctly.

**Fig. 2:**
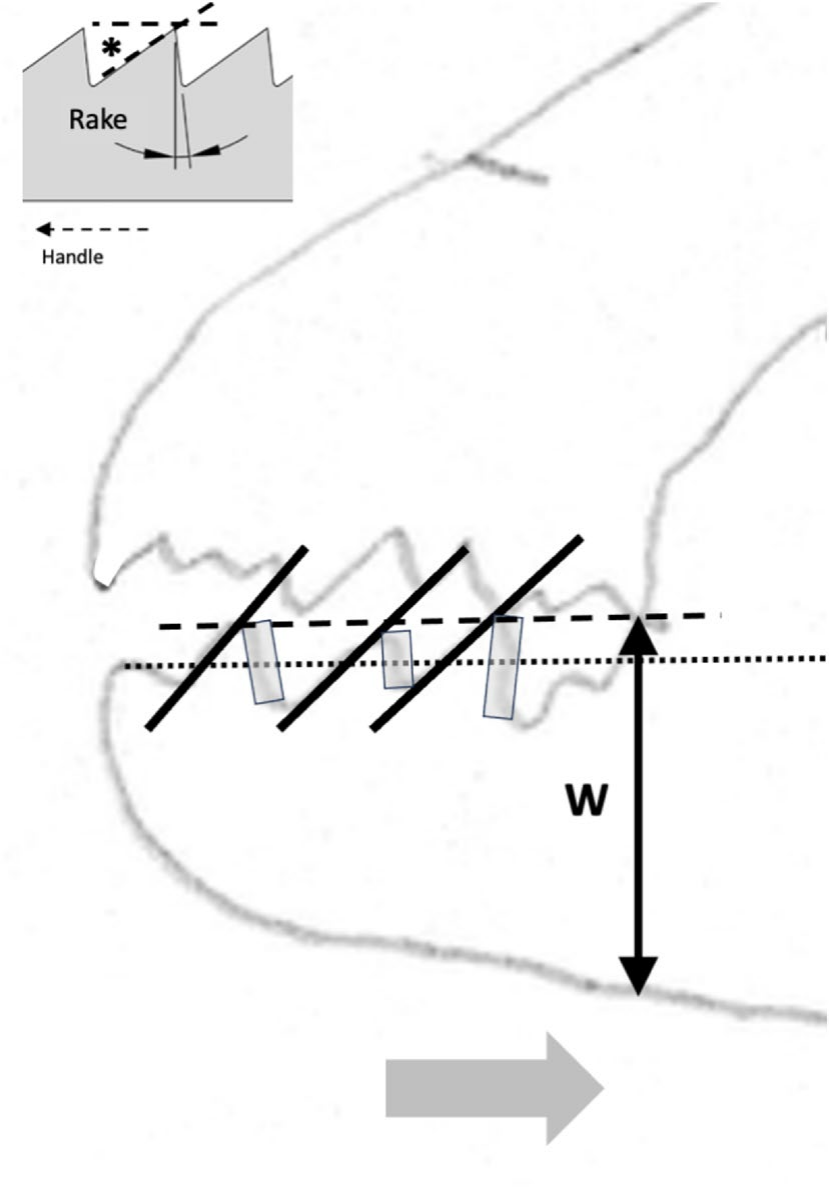
Illustrating back angle (denoted with *) compared to rake. Based upon *Glycyphagus domesticus* (specimen 224(1)−2) on retraction of the chelicera, large grey arrow). Amended from Bowman (2024a) with permission (Creative Commons Attribution 4.0 International License http://creativecommons.org/licenses/by/4.0/). Solid lines show approximately equal (positive) back angles for each gullet despite varying rake angles (point slope facets highlighted in grey boxes. Heavy double ended arrow indicates the saw ‘width’ (*W*) $\equiv$ moveable digit depth Bowman (2024b) at the end of the tooth row. Back angle from the last gullet to here (at *W*) can be estimated. The effective rake of the moveable digit (zeroth) tip can also be estimated

Many free-living astigmatans are saprophagous (see Flechtmann and Vacante 2024 for a recent comprehensive treatise). Tough, wood-like fibrous material must be encountered by free-living acarines in bird nests as they move around supposedly blindly. Plant derived material has some challenges for arthropod feeding (Bernays 1991), that has been overcome in many different ways by insects for example (Labandeira 1997). Wood like any surface can be ground or cut. In carpentry, crosscut saws are used to cut against the grain in wood, ripsaws are used to cut with the grain of the wood (http://www.vintagesaws.com/library/primer/sharp.html). Rasps are used to file excess material away. Wood is much less likely to chip and splinter when being cut with the grain. Taking this and other practical experience (widely available over the Internet) into account, then various acarological corollaries (enumerated 1–10) arise for individually scored astigmatan cheliceral moveable digit features when considered as possible saw-like tools, as follows:

1. Rake (Bowman 2024a) determines saw-blade operation. A saw of high positive rake angle (Fig. 2) will yield a fast feed rate and a very aggressive cut suitable for ripping wood (with the grain), but they “...are also more difficult to start.” (http://www.vintagesaws.com/library/primer/sharp.html). A low $<20^{\circ }$ (or negative) rake angle slows the feed rate, inhibits ‘binding’ of the material and the blade’s tendency to ‘climb’ the material being cut. Experience confirms, that it gives a more controlled cut with less ‘tear-out’ and smoother results cross-grain or on brittle material (like plywood and plastic). Chitinous exoskeletons may behave like such materials. SO: rake angles will be measured for the mite moveable digits.
2. The shape of the saw’s gullets is *per force* determined by the the rake angle and the back angle (= the number of degrees that the backside of the tooth leans backward of $90^{\circ }$, Fig. 2). Deep gullets (needed for increased detrital dust removal) thus have high back angles. Low back angles typify saws designed for softer material cutting than those with high back angles. High back angles need more power in the saw action and so can be used on tougher material. SO: back angles will be measured for the mite moveable digits.
3. Tooth height (= vertical distance from the tooth tip to the lowest point of the corresponding gullet) must be tall enough to allow the gullet to ‘carry out and away’ all the detrital dust from the cut, but need more power applied to the saw. $R_{t}$ is a good summary measure here. SO: $R_{t}$ will be measured for the mite moveable digits (both rising to the next peak (including $x_{i_{e}}$), and falling to the next valley). Indeed,
  Carpentry experience shows that blades with more teeth yield a smoother cut, and blades with fewer teeth remove material faster. Ripsaws have fewer teeth and are designed to quickly remove material (with some ‘scoring’ damage). However, a crosscut blade is designed to produce a smooth cut across the grain of the wood material without splintering or tearing it. Such a saw will have more teeth so that each tooth removes less material. However, as the saw moves through the material it *per force* makes many more individual cuts so it requires a slower feed rate of the material towards it. If the cut surface of a crosscut blade is examined it will look smooth and polished, with a cleaner cut at the edges. SO: the number of moveable digit peaks (teeth) will be subjectively scored and the location of their $R_{p}$ values measured.
  With a rip saw, experience shows that the feed rate can be faster and the wood-chip size is bigger. So the gullet (which is the space in front of each tooth allowing such chip removal) needs to be deep enough for the larger amount of raw material it has to handle. In comparison with the crosscut saw, such chips are smaller and fewer per tooth so the gullet is much smaller. Smaller gullets inhibit fast feed rates. Indeed, in order to produce a general purpose blade there are often larger gullets between groups of (small gulleted crosscut) teeth so as to help clear out the larger amounts of material generated when ripping with the grain. The small gullets within the grouped teeth inhibit too fast a material feed rate when cutting across the wood grain. SO: the number of moveable digit valleys (gullets) will be subjectively scored and the location of their depth $R_{v}$ values measured..
4. Tooth shape is important in saw function. Many crosscutting hand saws have a larger tooth zenith angle than rip saws SO: the angle at the zenith of each peak will be calculated for the mite moveable digits.
5. Low power sawing uses shorter tooth spacing (= distance from one tooth tip to another horizontally) or higher pitch (Bowman 2024a). An arrangement of wide tooth spacing i.e., lower pitch, characterises high power saws. SO: inter-tooth zenith distances (horizontally along *L2M*) will be measured for the mite moveable digits.
6. Gullet arrangement is important in saw function (see Bowman 2024a). SO: inter-gullet spacing (= distance from one gullet nadir to another horizontally along *L2M*) will be measured for the mite moveable digits.
7. Gullet shape is important in saw function. Crosscutting hand saws have a larger gullet nadir angle than rip saws SO: the angle at the nadir of each gullet will be calculated for the mite moveable digits and compared to the ‘hook’ or ‘claw angle’ ($\gamma$) from Bowman 2024a.
8. The blade width (*W*) of a saw is the distance between the tip of the teeth and the base of the blade (Fig 2). Bowman (2024b) models this moveable digit depth in terms of a stack of declining length longitudinal (zero intrinsic curvature) filaments resisting occlusive forces. Consiliently, in practice deeper saw blades match higher power input and fast feed rate ripping. Narrow blades match more difficult sawing and lower power inputs. SO: moveable digit depth (*W*) at the posterior end of the tooth row ($x_{i_{e}}$) will be measured.
9. Saw blades are available in ‘full-kerf’ and ‘thin-kerf’ varieties depending upon the width of the sawn slot (or kerf) which the blade cuts into the material. It includes the blade thickness plus any tooth ‘set’ (Bowman 2024a) measurements, to give the total amount of wood removed by the blade with each pass. Experience shows that full kerf blades require more power to move them through the material as they must remove more material. Thin kerf blades have a less tendency to bog down during cutting at inappropriate feed rates however they are more flexible and prone to vibration (= loss of stability). Thin kerf blades have the advantage of less material lost as detrital dust, so material is divided up more efficiently (although the item size is smaller). Now chelae are sub-cylindrical in form being taller than wider (Bowman 2020, 2021, 2023a, decreasing posteriorly$\rightarrow$anteriorly Bowman 2024a). They sit laterally next to each other but usually alternatively work one at a time with broadly spherical basal rami (Bowman 2024a). SO: kerf will be equated to $0.5*max([2*L1U],[2*W])=max(L1U,W)$ and compared to $thick=0.298*CHI$ using Bowman 2021, 2023a).
10. Teeth tips in saws also matter
  The flat-top tooth (FT) design is the most efficient for cutting and raking material out of the cut (such as for ripsaws which quickly and efficiently remove material).
  Alternate top bevel teeth (ATB) form a knife-like edge on either side of the blade and make a cleaner cut than flat top teeth. They yield a smoother cross-cut on wood and veneers. This is particularly so for high alternate top bevel teeth (HiATB) saws where the higher bevel angle increases the knife-like action at the edge of the blade. Such shearing action is needed for extra-fine crosscutting or cutting materials prone to chipping like those surfaced with melamine. Chitin (as a bonded fibrillar laminate) might behave like this.
  Arrangements of one FT and four ATB teeth are found on general purpose combination saw blades, with a large gullet between the groups of five.
  Triple chip grind (TCG) configured teeth are excellent at cutting hard materials such as laminates, medium density fibre board and plastics, Chitinous exoskeletons are also structured somewhat like these. TCG teeth alternate between a raking flat tooth and a higher ‘trapeze’ tooth. This configuration is also useful for cutting non-ferrous metals. Predatory acarines should carry such a tooth design. SO: an attempt will be made to subjectively but systematically classify each tooth with the preliminary binary choice ‘approximately FT’ or ‘likely to be ATB’, pending SEM and 3D morphological follow-up work to investigate if any are actually faceted more like a TCG arrangement using methods like Winchester (2016) which were previously used in reptile jaw studies (Melstrom 2017).

Together with the digitised moveable digit profile projected onto the reference axis (= adductive output moment lever arm *L*2*M*, Bowman 2024a) and the measured length of the adductive input moment lever arm (*L*1*U*, Bowman 2021), available laboratory samples of the bird nest community mites will be assessed as above in order to understand better how the different species might co-exist with little trophic competition. Some measures are best summarised and analysed as between species, some as between individuals within species and some as between mastication surface locations within individuals of a species.

It is expected (like free-living mustelids, Selig 2023) that the various mite species will show morphological divergence into different feeding style ‘niches’ within the bird nest habitat reflecting their reliance on tough or soft foods, together with evidence of convergence on aspects of form that relate to recurrent ‘everyday behaviour’ (e.g., foraging, feeding etc.,) across the established familial taxonomic groups (see Bels and Russell 2023). It is expected that cheliceral versus chelal versus moveable digit profile adaptations may vary independently (Bowman 2024a). Multiple adaptive zone shifts (Arbour et al. 2019) may be indicated. As the actual sizes of structures matter, covariance-based analyses rather than correlation-based analyses will be used when necessary.

Bowman (2024a) points to the commonality of various mite digit features with hooks and claws. Using such devices can be categorised into three ‘functional spaces’: piercing, gryporial hook-and-pull, and scalporial scratch-digging (Qin et al. 2023). The first is facilitated in mites by cheliceral protrusion, the latter two by cheliceral retraction, all three augmenting any crushing or cutting function of the chela. Evolutionary models suggest that claw shape evolves toward multiple adaptive peaks, with structural habitat use pulling species toward a specific selective optimum (Baeckens et al. 2019).

Bowman (2024a) found that beehive inhabiting mite "...mastication surface macro-roughness values..." were "...in the range of international Roughness Grade Numbers N5-N6.". This makes them suitable for grinding to ‘finish’ surfaces via abrasion (see https://www.an-engineering.co.uk/surface-finish-chart/). Such burnishing (a squeezing failure mode of sliding material, https://en.wikipedia.org/wiki/Burnishing_(metal)) or overall finishing (https://en.wikipedia.org/wiki/Linishing) of foodstuff may occur along with peening (i.e., chelal asperity ‘hammer-blows’,https://en.wikipedia.org/wiki/Peening) to "...expand surfaces via compressive stresses..." and "...releasing tensile stresses already present..." therein. If loose abrasive material is available as finer abrasive agents in the gnathosomal area then buffing (https://en.wikipedia.org/wiki/Polishing_(metalworking)) and lapping (https://en.wikipedia.org/wiki/Lapping) of the foodstuff could occur.

So, several questions arise:

- To what extent is the design differentiation already found amongst astigmatan co-inhabitants of bee hives supported in another commensal community (e.g., bird nests)?
- Is there evidence of the three previous particular (beehive mite) designs (or more extreme versions suitable for very hard food or even liquids)?
- Furthermore, do bird nest astigmatans span the three functional spaces (aka Qin et al. 2023) and so can avoid trophic competition?

Even though the skulls of herbivorous dinosaurs, for example, are fairly similar to each other in overall shape, the way they worked during biting is substantially different in each case (Lautenschlager et al. 2016). So a detailed understanding of how tools like saws (and jagged blades) are designed to function is important. The profile of a saw is like a set of actually distinct small sharp hooking and cutting surfaces. These are pushed or drawn through material cutting (and tearing) it. Therefore the stochastic distribution of such asperities is important in their ability to grind things. For any nidicolous mite species with a saw-like design to their moveable digits, could inferences be made as to how the chelae might be actually used on nest material? Note that pushing a jagged surface through material is different than pulling it. A saw blade can be designed to cut when pulled (Japanese style) or designed to be pushed (Western style) in order to cut material. The Technical Appendix explains how these two designs relate to each other and to mite dentate moveable digits. Also recall that asperities can act as ‘grippers’ on chelal closure as well.

The use of bio-mechanically meaningful measurements improves confidence in the ability to infer function based on morphology (Thomson and Motani 2021). However, even when structural descriptors are based upon bio-mechanical analyses, function still cannot be inferred unambiguously from morphology alone (Swartz et al. 2003)—confirmatory experimental manipulations to validate ideas are needed.

Geometric morphometrics with its attendant thin-plate splines and principal components analysis is a useful summary of *intrinsic* shapes and their variation. However, it is the *actual* size, position, and orientation of the chelal moveable digit features that Nature sees in the phenotype. The latter is what this ecomorphological study explores. Two extremum instances of possibly murderous weaponry that could be unthinkingly condensed into a single Procrustean analysis illustrate this. You cannot be more easily killed by a miniature ‘dolls-house’ toy plastic axe (originally held upside down ’at a thousand yards’ to your skin) no matter how sharp, than a ‘normal-sized’ blunt dagger (originally held perpendicularly close to your head)—yet both would be normalised by the re-scaling, rotating and translating analytics and the former design ‘preferred’. A heavy dagger is the one to worry about in actuality. *Intrinsic* shape is not the issue for the use of weaponry. As J Clements points out (What Makes an Effective Sword Cut? https://www.thearma.org/essays/howacutworks.htm): "...A laceration can of course be made by striking on the human body with almost any object (e.g., a car antenna or even a spoon) provided you have enough velocity behind it—it won’t sever tissue or deeply shear but it will tear and scratch...", so it is more important as how a tool is actually used for real than its intrinsic (Procrustean) shape. Indeed recently Bookstein (2024) introduces a new method eschewing Procrustean co-ordinates to explain actual growth.

This comparative biology study unashamedly uses observed pattern to infer process. It relies upon natural variation existing in mites for a primary adaptive functional reason (herein a ‘mechanism’), as opposed to arising from neutral random or (decimatory) contingent chance. As bumblebees and social wasps are non-competitive occupants of bird nest boxes (Broughton et al. 2015) erected to stimulate bird breeding, particular notice will be taken of comparisons to bee associated acarines. The species investigated by Bowman (2023a) should share nest box habitat features but not necessarily unique bird nest inhabiting adaptive features. Similarly not only are nests and nest boxes a feature of some mammal lifestyles (Jaworski et al. 2022), cadavers (whether wet or dry) could also be considered a similar necrophagous protein source to bird nests. Comparison to astigmatan species from these sources could probe which features really might be important for mite success in the bird nest habitat.

**Table:** 

Glossary of abbreviations	
Abbreviation	Meaning
$\alpha$	Subtended angle for chela ex Akimov and Gaichenko (1976)
$\eta _{p}$	Number of peaks
$\eta _{v}$	Number of valleys
$\gamma$	‘Claw’ or ‘hook’ angle
$\varPi$	3.142....
$\sigma ^2$	Tribological variance
$\varTheta$	Hang angle *sensu* Isaac Smith
$\varOmega$	Semi-definite profile matrix (see Bowman 2024b)
ATB	Alternate top bevel tooth
*b*	Width
$b_{i}$	Back angle at semi-landmark *i*
*BFQ*	Bite force quotient (regression residual from *F*2 bite force by body mass ($IL^3$)
$c_{i}$	Profile curvature at *i*th semi-landmark
*CHI*	Cheliceral height index
*CLE*	Claw length equivalent
*CLI*	Reach or cheliceral length index
*df*	Difference in flexure
$F_{b}$	Downward force on material at saw teeth/support from board *sensu* Isaac Smith
$F_{c}$	Resistance of the material to being cut by the saw *sensu* Isaac Smith
*F*1	Chelal adductive input lever arm muscular force ($PHI^2 \equiv CHI^2$
	in Bowman 2020)
*F*1*AV*	Chelal adductive input lever arm muscular force in Bowman (2020)
*F*2	Occluding chelal crunch force (*VR*.*F*1)
*F*2*AV*	Occluding chelal crunch force (*VR*.*F*1*AV* in Bowman 2020)
$fR_{t}$	Falling total $R_{t}$
FT	Flat top tooth
$g_{i}$	Profile gradient at *i*th semi-landmark
*h*	Shear or creep measure
*H*	Height
HiATB	High alternate top level tooth
*IL*	Idiosomal length index
*L*1*U*	Adductive input moment lever arm
*L*2*M*	Adductive output moment lever arm
*m*	Length of mastication surface (‘drape’ or ‘chain’ distance)
	over asperities/gullets (consequence of stretch)
*MDL*	Moveable digit length (or ‘Gape’ approximation)
*na*	Gullet nadir angle
*ogcar*	Aspect ratio $\frac{fR_{t}}{x_{i_{e}}}$
*r*	Correlation coefficient (statistical use)
*rel*	Relative elongation
$R^{2}$	Percentage variation explained
$R_{a}$	Tribological average absolute deviation, mean surface roughness
$r_{i}$	Rake angle at semi-landmark *i*
$R_{p}$	Distance between highest asperity and average, highest peak (at $x_{Rp}$)
$R_{q}$	Tribological *Root Mean Square*
$R_{t}$	Distance from highest peak to lowest valley
$R_{v}$	Distance between average and lowest valley, lowest gullet (at $x_{Rv}$)
*RMS*	Root mean square $R_{q}$ ex Bowman (2024a)
$rR_{t}$	Rising total $R_{t}$
SEM	Scanning electron microscopy
*t*	Thrusting distance
*tan* ^*-1*^	Arctan function
*tel*	Stretch measure ex Bowman (2024a)
TCG	Triple chip grind (tooth)
TCSA	Tip cross-sectional area
*Var*	Variance
*W*	Moveable digit depth at the end of the mastication surface
*VR*	Adductive moveable digit lever arm velocity ratio $\frac{L1U}{L2M}$ (or
	mechanical advantage Bowman 2020)
$x_{i}$	Moveable digit co-ordinates along L2M
$x_{i_{e}}$	End of mastication surface (*e*) distance along *L*2*M* axis towards condyle
$y_{i}$	Moveable digit height at location $x_{i}$
*za*	Tooth zenith angle

## Materials and methods

Preserved samples of mite individuals from previous work (i.e., Bowman 2021) were used. Mites were cleared in lactic acid and examined as wet mounts with Nomarski phase contrast microscopy. Drawings with micrometer scales were made using a Zeiss research microscope drawing tube. Twenty adult female specimens were used of each bird nest inhabiting taxon (Table 1) for locating the 18 projected profile data points to ensure the full rank of matrices and comparison to earlier work. The taxa reviewed cover the four feeding habit dimensions of ‘Surface-living’ versus ‘Interstitial’ by ‘Omnivore’ versus ‘Fragmentary feeder’ (Bowman 2021). No mites from the family Histiostomatidae (or anoetids) were used. Samples of other mites known from bird nests: *Austroglycyphagus* spp., *Blomia* spp., *Campephilocoptes* spp., *Carpoglyphus nidicolous*, *Dermacarus pilitarsus*, *Dermatophagoides evansi*, *Echimyopus orphanus*, *Euglycyphagus intercalatus*, *Fusacarus* spp., *Glycyphagodes spheniscicola*, *Glycyphagus ornatus*, *Gymnoglyphus longior*, *Hirstia chelidonis*, *Neoxenoryctes reticulatus*, *Psylloglyphus parapsyl!us* (Winterschmidtiidae), *Sapracarus tuberculatus*, *Schwiebia talpa*, *Tyrophagus mixtus*, *Tyrophagus formicetorum* and *Mycetoglyphus fungivorus* were not available to the author.

**Table 1:** Free-living astigmatans reported as common in bird nests available as laboratory samples (from Hughes 1976, Solarz 2004, Kew et al. 2015) grouped by genus

Family	Taxon	‘Presence in other habitats’	Population
Acaridae			
	*Acarus farris*	0100100111	A17
	*Acarus gracilis*	1000000000	A4
	*Acarus immobilis*	0000000101	A1*
	*Aleuroglyphus ovatus*	0100100010	AL2$^\dag$
	*Rhizoglyphus robini*	0000000001	R1*
	*Thyreophagus entomophagus*	0000000010	TH3*
	*Tyrolichus casei*	0100000110	T62*
	*Tyrophagus longior*	0000100101	T40*
	*Tyrophagus palmarum*	0000000101	T17*, T32
	*Tyrophagus putrescentiae*	0000010110	T13
	*Tyrophagus similis*	0000000101	T21*, T44
Chortoglyphidae			
	*Chortoglyphus arcuatus*	0011100010	CH1$^\dag$
Glycyphagidae			
	*Glycometrus hugheseae*$\ddag$	0000000000	G3$^\dag$
	*Glycyphagus domesticus*	1011001110	G5$\dag$
	*Lepidoglyphus destructor*	0100100011	G6$^\dag$*
Pyroglyphidae			
	*Dermatophagoides pteronyssinus*	0011000000	D3
Suidasidae			
	*Suidasia pontifica*$\ddag \ddag$	0000000110	S5

$^\ddag$was *Austroglycyphagus geniculatus*,$^\ddag{^\ddag}$was *Suidasia medanensis*. ‘Presence in other habitats’ records from Hughes (1976) not including feathers, nor fruit. Number string: 1 = positive, 0 = negative, in the order: Bat roosts, Mammal nests, Mattresses, Dust, Broiler houses, Cadavers (Braig and Perotti 2009), Meat, Cheese, Storage, Grassland. Population ex Bowman (2021).$^\dag$Omnivore (others are Fragmentary feeders). *Surface habit (others are Interstitial)

The methods (see Glossary of Abbreviations) used were those of Bowman (2024a) who makes a distinction between angles and curvatures. The tooth-line was always oriented horizontally (i.e., *L*2*M* is the reference direction). It is assumed that forces at the teeth act at a single point.

All data manipulations were carried out in Excel for Mac version 16.78.3 and R version 4.3.1 (2023-0616) ‘Beagle Scouts’. Analysis of angle data used the package ’circular’ where necessary. Appropriate reduced subsets of measurements were used in analyses as some parameters are linearly dependent.

### A model for actual asperities

Measurements for each individual were made for the: moveable digit depth at the end of mastication surface (*W*), back angle at *W*, number of peaks (teeth), number of valleys (gullets), tooth rake angles (including moveable digit tip), tooth back angles, inter-tooth distances, inter-gullet distances, rising totals *rRt*(*i*), falling totals (including moveable digit tip) *fRt*(*i*), tip angle, and the digit distal angle ($=tan^{-1}[\frac{W}{x_{i_{e}}}]$), see Glossary of abbreviations.

The tooth angle (in $^{\circ }$) at the zenith of the $ith\ peak=90-rake_{[ith\ peak]}-back\ angle_{[ith\ peak]}$ by simple trigonometry. The gullet angle (in $^{\circ }$) at the nadir of the $ith\ valley=90-rake_{[(i-1)th\ peak]}-back\ angle_{[ith\ peak]}$ by simple trigonometry (where the 0th peak $\equiv$ the moveable digit tip).

The subtended angle $\alpha$ used by Akimov and Gaichenko (1976) (see Fig. 11 in Bowman 2024a) was calculated assuming that, the moveable digit tip $\rightarrow$ condyle $\rightarrow$ adductive tendon insertion at the top of basal ramus $\rightarrow$ moveable digit tip, was a scalene triangle. Then $\alpha =90-\gamma$ in $^{\circ }$ using the formula for $\gamma$ given in Fig. 24 of Bowman (2024a).

Given such separate explicit scoring of different numbers of visible features across each specimen, one can pose a model growth mechanism for the individually measured horizontal ramus asperities (i.e., the teeth and gullets, counting separately in order from the moveable digit tip), as follows

- An individual particular tooth ‘grows’ and it becomes more peaked i.e., its zenith angle ($za_{j}$, for $j=1...no.\ of\ peaks$) becomes smaller starting from a totally flat $+180^{\circ }$ to approach a highly pointed impossible $0^{\circ }$ from above (and vice versa for each ‘tooth’ shrinkage)
- An individual particular gullet ‘grows’ and it becomes deeper i.e., its nadir angle ($na_{k}$, for $k=1...no.\ of\ gullets$) becomes smaller starting from a totally flat $+180^{\circ }$ to approach a highly pointed impossible $0^{\circ }$ from above (and vice versa for each ‘gullet’ shrinkage).

Then,

- $\sum _{j=1}^{no.\ of\ peaks}(za_{j})$ can be modelled versus *j* for each species across each individual,

as well as separately

- $\sum _{k=1}^{no.\ of\ gullets}(na_{k})$ can be modelled versus *k* for each species across each individual,

and appropriate comparisons of the summarised moveable digit mastication surface’s local flexure (*tel*) if necessary separately, ‘up’ as teeth or ‘down’ as gullets, then can made across species categorised by similar functional forms ($\equiv mastication\ tool\ type$).

Further,

- As a peak (or the rise in the ascending ramus) must follow a gullet, or a gullet must follow the moveable digit tip or any peak $\Rightarrow no.\ of\ gullets= \eta _{v}=1+no.\ of\ peaks=1+\eta _{p}$

The *TurtleGraphics* library in R may be used to illustrate typical forms. Split-plot (mixed effects) analysis will be used to model between and within individual measurements using *nlme* in R.

## Results

Measurements for each individual were made for: the number of peaks (teeth), number of valleys (gullets), tooth rake angles (including moveable digit tip), tooth back angles (including that at *W*), inter-tooth distances, inter-gullet distances, rising totals *rRt*(*i*), and falling totals (including moveable digit tip) *fRt*(*i*) and are summarised graphically. Illustrative comparative data (with matching relevant modes of action) are presented in Table 2 (more vertebrate examples could be chosen from https://en.wikipedia.org/wiki/Handbook_of_the_Mammals_of_the_World).

**Table 2:** Illustrative comparative data of human tools and various animals. Zenith and nadir angle measured as in Fig. 1

Object	Zenith angle ($^{\circ }$)	Nadir angle ($^{\circ }$)	*σ zenith*	*σ nadir*	Stated role	Origin
Archaeopteryx jaw (Aves)	102.6^*^	99.8 †	15.4	7.9	Biting	Wellnhofer (1990)
Arrow heads (Thermopyles) *convex edge*	146.2	–	6.5	–	Slicing	National Archaeological Museum, Athens, Greece
Bronze Axes Iron Age	144.3	–	–	–	Chopping	Motya, Sicily, Italy
Tomb 82	153.6	–	3.4	–	Chopping	National Archaeological Museum, Athens, Greece
Bronze Razors (Tombs 26, 56)	147.0	–	2.3	–	Slicing	National Archaeological Museum, Athens, Greece
Circular saws (standard gauge)						Disston & Sons (1918)
No 1	33.3	45.2	4.3	20.4	Ripping/Crosscutting	
No 2	51.1	56.1	4.7	8.0	Crosscutting	
No 4	51.1	48.6	1.7	5.2	Crosscutting	
No 5	42.2	74.4	4.1	0.4	Crosscutting	
No 6	27.8	49.4	2.5	7.4	Ripping/Crosscutting	
No 8	43.3	93.3	3.5	6.3	Ripping/Crosscutting	
No 11	27.4	50.9	–	7.2	Ripping	
No 12	39.2	53.3	–	7.5	Ripping	
No 13	39.7	56.3	–	0.8	Ripping	
No 14	35.7	59.4	–	0.5	Ripping	
No 17	43.7	99.5	9.0	25.8	Crosscutting	
No 18	28.4	33.5	5.7	7.3	Crosscutting/Mitreing	
Cookie-cutter shark jaw	50.7	50.7	4.5	5.6	Biting	Simon (2013)
Copper war axe	133.4	–	–	–	Chopping	Chalcolithic, Danube River Valley, Balkans
Fish *Aplodinotus grunniens*						[Internet image]
pharyngeal teeth	118.2	–	10.3	–	Crushing	
*Archosargus probatocephalus*						[Internet image]
front teeth	149.7	–	20.1	–	Crushing/Scraping	
*Pogonias cromis*						[Internet image]
pharyngeal teeth	98.1	–	12.2	–	Crushing	
Flint hand-axe	134.4	–	–	–	Chopping	Neolithic, Medway Gravel Pits, Medway, Kent, UK
	70.8	–	–	–	Chopping	Neolithic, Sussex Archaeological Society, 2005-12-15, UK
	115.2	–	–	–	Chopping	Neolithic, Malton Museum, NYorks, UK
	100.9	–	–	–	Chopping	Neolithic, Brora Heritage, Brora, Scotland
	159.0	–	–	–	Chopping	Neolithic-Bronze Age, Bridlington, Yorkshire, UK
	135.0	–	–	–	Chopping	Neolithic-Bronze Age, Hertfordshire, UK
Grappling hooks	–	71.8	–	21.5	Hooking	20 random images from Internet
Hand saws						Disston & Sons (1918)
4-point (p128)	64.0	63.4	3.2	3.0	Ripping	
5-point (p128)	70.7	69.8	3.3	4.8	Crosscutting	
D17 (p133)	51.9	53.9	4.8	15.5	Diagonal cutting	
*Iguanodon maxilla (Dinosauria)* *Comptonotus chasei*	158.6	–	31.6	–	Grinding	Lockwood et al. (2024)
Jadeite hand-axe	112.8	–	–	–	Chopping	Neolithic, Craven Museum & Gallery, Skipton, Yorkshire, UK
Lawn-edging garden tool	98.3	–	–	–	Slicing	Ironmongery/Hardware store UK
Mammals Eurasian water shrew mandible	76.5	87.0	13.6	24.1	Biting	Tiberti and Mori (2016)
Mustelid mandibles *Martes foina*	82.8	88.3	13.6	22.5	Chewing	Pleistocene, Cave Deposits, Transylvania, Romania
*Megalictis ferox*	83.4	95.8	21.4	20.9	Chewing	Valenciano et al. (2016)
*Mustella frenata*	82.4	89.7	26.9	22.3	Chewing	University of Michigan Museum of Zoology, USA
*Permodiadonta oklahoma* (Paramhibia)						McMenamin (2015)
tooth crowns	114.8	–	17.0	–	Crushing	
Reptiles *Aethesia frangens*						Hutchinson and Scanlon (2009)
tooth crowns	85.1	–	15.1	–	Crushing	>
Alligator mandible						[Internet image]
Tooth summits	68.6	–	27.4	–	Gripping/Cutting	
*Chromatogenis tiliquoides*						Makádi and Nydam (2014)
mandible	103.6 **	92.8$\ddag$	28.4	25.7	Crushing	
tooth crowns	118.9	–	13.6	–	Crushing	
in between teeth	–	143.4	–	0.9	Crushing	
*Tiliqua scincoides*						Makádi and Nydam (2014)
maxilla	126.5	126.4	14.2	19.2	Crushing	
*Tubinambis teguixin*						Makádi and Nydam (2014)
dentary	83.2**	84.68$\ddag$	23.4	25.6	Crushing	
tooth crowns	98.5	–	22.1	–	Crushing	
Spear heads						National Archaeological Museum, Athens, Greece
Thermopyles						
*leaf − shaped edge*	141.7	–	–	–	Slicing	
Tombs 47, 82						
*leaf − shaped edge*	157.5	–	9.7	–	Slicing	
Socket sets (p8)						Anon (1964)
6 point	–	120	–	–	Gripping	
8 point	135	90	–	–	Gripping	
12 point	145	110	–	–	Gripping	
Strigil Roman	–	140.1	–	7.7	Scraping	Harvard Art Museum, USA
Roman	–	145.2	–	9.2	Scraping	Metroiploitan Museum of Art, USA

*Note* *Overestimated by method herein as each peg-like tooth on its own (i.e., ignoring the gullet either side of it) is approximately half this zenith angle (mean $=53.0$, $\sigma =13.2$). $^\dag$Underestimated by method herein as the jaw surface is essentially horizontal between widely dispersed scimitar shaped teeth. ** = Underestimated by method herein as blunt shaped teeth separated by deep gulleted surface between. $^\ddag$Overestimated by method herein as deep gulleted surface between blunt shaped teeth. A crushing role is assigned to known durophages

**Fig. 3:**
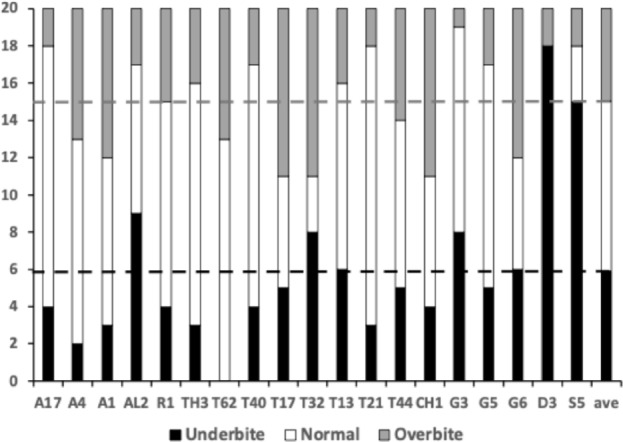
Number of individuals out of 20 whose cheliceral chela exhibits underbite (black columns) and overbite (grey columns) in bird nest astigmatans (with respect to their moveable digit). Dashed lines = relevant means

Figure 3 shows the degree of moveable digit underbite suitable for a leveraging material up (Bowman 2025b) or for a ploughing action (Bowman 2025b). Overall, there is an $\approx 50:50$ split between normal occlusion chelate-dentate chelae and ‘abnormally’ occluding chelae. There is also within the derived occluding chelae an $\approx 50:50$ split between those showing underbite and those showing overbite. It is therefore interpreted that the bulk of the observed differentiation between species could be due to microscope slide artefacts (i.e., deformation of a 3D form on lateral compression to 2D). However, *Dermatophagoides pteronyssinus* D3 and *Suidasia pontifica* S5 noticeably show most chelae with underbite and *Tyrolichus casei* T62 shows no individual with any underbite at all. This does suggest a different feeding action in these three taxa. The two former taxa being able to lever up or prise open food material (see Bowman 2025b), the latter not at all.

No bird nest astigmatan taxa had a moveable digit tip velocity ratio value as extreme as that in wild-collected *Carpoglyphus lactis* from beehives at 0.310, Bowman 2023a, nor as extreme as that for *Carpoglyphus lactis* Ca4 at 0.355 Bowman (2021), nor as extreme as that for *Carpoglyphus lactis* Ca4 at 0.358 in Bowman (2024a). Rather the range of per-taxon averages were $0.402-0.583$ (for *Acarus farris* A17 to *Chortoglyphus arcuatus* CH1). Velocity ratio values for *Glycyphagus domesticus* G5 at 0.458 and *Tyrophagus putrescentiae* T13 at 0.409, were in strong agreement to those of wild-collected *Glycyphagus domesticus* at 0.459 and wild-collected *Tyrophagus putrescentiae* at 0.409 from beehives, Bowman (2023a). The value for *Glycyphagus domesticus* G5 also agrees with the velocity ratio value of 0.456 in Bowman (2021) and 0.455 in Bowman (2024a). The value for *Tyrophagus putrescentiae* T13 also agrees with the velocity ratio value of 0.411 in Bowman (2021). However, note that *Tyrophagus putrescentiae* T13 and the Museum specimens of *Tyrophagus putrescentiae* in Bowman (2024a) *VR* values are much higher at 0.430 and 0.461 respectively.

No bird nest astigmatan taxa has a moveable digit tooth row ($x_{i_{e}}$) as long as that in wild-collected *Carpoglyphus lactis* from beehives at $20.1\mu$m (Bowman 2023a) nor *Carpoglyphus lactis* Ca4 at $17.3\mu$m in Bowman (2024a). Rather the range of per-taxon averages were $10.4-19.4\mu$m (for *Suidasia pontifica* S5 to *Tyrolichus casei* T62). Tooth row length values for *Glycyphagus domesticus* G5 at $16.6\mu$m and *Tyrophagus putrescentiae* T13 at $15.1\mu$m, were somewhat larger than those of wild-collected *Glycyphagus domesticus* at $14.8\mu$m and wild-collected *Tyrophagus putrescentiae* at $14.2\mu$m from beehives, Bowman (2023a). The average for *Glycyphagus domesticus* G5 herein is slightly shorter than the $14.0\mu$m for *Glycyphagus domesticus* G5 in Bowman (2024a). The average for *Tyrophagus putrescentiae* T13 herein is markedly larger than *Tyrophagus putrescentiae* T13 at $13.1\mu$m or the Museum specimens at $10.8\mu$m in Bowman (2024a).

**Fig. 4:**
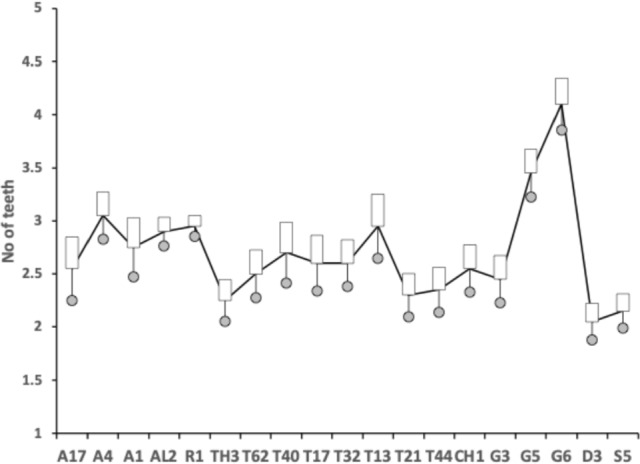
Observed data for number of teeth ($\eta _{p}$, between tip and ascending ramus) on a bird nest astigmatan moveable digit. Note that number of gullets = number of teeth + 1 axiomatically. Bar = upper $95\%$ confidence interval. Dot and line lower $95\%$ confidence interval. Species ordered in families and then alphabetically by genus and species (with line through species means (over individuals) to highlight differences. Multiply mean value by 2 to give the approximate matching socket (wrench) or tube cutter design (*see text*)

The number, pattern (and size) of moveable digit asperities is visibly different amongst these astigmatans (Fig. 4). Variation in tooth number has been used as a strategy by many mammals to develop specialized dentitions and has been an important factor for species diversification. (Line 2003). The minimum number of teeth (ignoring the digit tip) was 1 (e.g., *Dermatophagoides pteronyssinus* D3), the maximum 5 (e.g., *Glycyphagus domesticus* G5, *Lepidoglyphus destructor* G6) resulting in the corresponding range in the number of gullets of 2–6. A typical bird nest astigmatan moveable digit has three teeth (and therefore four gullets between the tip and the ascending ramus) - just like *Carpoglyphus lactis* (Bowman 2023a). Fig. 4 shows that the two widely occurring glycyphagids have markedly more teeth and gullets compared to the other species studied i.e., the moveable digits of *Glycyphagus domesticus* G5 and *Lepidoglyphus destructor* G6 are more saw-like. Scoring individual teeth rather than deriving the count from whole-profile fluctuations (as in Bowman 2024a) gives a more accurate value for the multi-dentate *Glycyphagus domesticus* G5.

If the typical bird nest astigmatan mastication surface was repeated (in an inverse matching sense) along the fixed digit, then the whole chelal assembly encompassing any food morsel approximates an exchangeable ‘socket’ used with torque wrenches (see https://en.wikipedia.org/wiki/Socket_wrench). Indeed different chelate-dentate mites might be mapped to square 4-point, specialised 5-point, hexagonal 6-point, specialised 7-point, 8-point, 9-point, 10-point, 11-point or even bi-hexagonal 12-point sockets (where $point=1+(2*(no\ of\ teeth+1)))$ for an open chela and common ‘ascending ramus tooth’ region where the fixed and moveable digits join.

Matching the facets against the flanks of foodstuff moieties of the right size and shape would allow more tearing torque to be applied (as the chelicerae are moved around) - just like wrenches for car wheel-lock bolts. This is because if such sockets overly rely upon their tips which do not match the bolt’s topology to solely grip, then there is always the tendency for the material to deform and slip unless forces are moderated. Of course a wider variety of food material sizes and shapes could be grasped by say a 12-point socket-analogue chela versus a 6-point socket-analogue, so evolutionarily this versatility may be of use to a mite. However, as old motor engineers say "...In the world of sockets, there is no one-size fits all.".

### Modelling actual asperities—rake and back angle

If one simply scores the actual individual asperities on the chelal moveable digit then the intrinsic reference axis of the output moment arm *L*2*M* can help.

Figure 5 shows that

- in general bird nest astigmatans have broadly similar proximal rake angles around $-48^{\circ }$
- with the exception of *Chortoglyphus arcuatus* CH1, *Dermatophagoides pteronyssinus* D3, *Glycometrus hugheseae* G3 and *Lepidoglyphus destructor* G6 (at around $-56^{\circ }$), distal rake angles are around $-70^{\circ }$
- back angles in bird nest habitat astigmatans are usually $\approx 43^{\circ }$ distally but $\approx 33^{\circ }$ proximally
- the back angle for *Glycyphagus domesticus* G5 and *Tyrophagus putrescentiae* T13 are markedly polymorphic proximally.

**Fig. 5:**
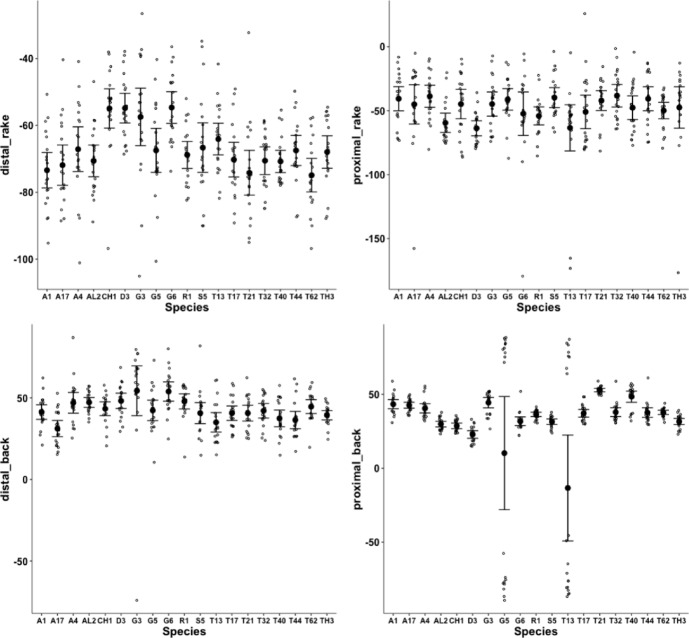
Observed data (small open dots) with mean (large black dot) and $95\%$ confidence intervals (as ‘T’ bars) by species (in alphanumeric order) for moveable digit angles in $^{\circ }$. Top row = rake angle (left = distal i.e., digit tip, right = proximal i.e., last tooth before end of mastication surface and rise of ascending ramus. Bottom row = back angle (left = distal i.e. after first gullet post-tip, right = after last gullet to end of mastication surface

These rake angles are markedly different than the leading edge (on digit retraction) $12-15^{\circ }$ values used for cross-cut saws or the $8^{\circ }$ used for rip saws (http://www.vintagesaws.com/library/primer/sharp.html). Such saws with back angles at $\approx 38-45^{\circ }$ (http://www.vintagesaws.com/library/primer/sharp.html) however do match the trailing edges of the astigmatan mastication surface (on digit retraction). This all implies that the mites have an ‘easy to start moving non-tearing’ well-controlled cut of food material with very gently shaped attack surface teeth. Of course, an element of this apparent gentleness is the continuous linear approximation method used herein (Fig. 1). Scimitar shaped cutting facets (like in *Archaeopteryx*, Wellnhofer 1990) would have initial rake angles much nearer zero. A follow-up SEM study could model the mites’ moveable digit profiles in more detail.

### Modelling asperities - zenith and nadir angles

Rake and back angle combine to determine a tooth’s zenith angle and a gullet’s nadir angle irrespective of the actual location of such. The sharpness of teeth is observably correlated with diet in lizards (see http://www.savalli.us/BIO370/Anatomy/5.LizardSkullLabel.html). Moreover, tooth shape is routinely used to infer carnivory versus herbivory versus durophagy in fossil lizards (e.g., illustrative Fig. 2 in Gere et al. 2021).

**Fig. 6:**
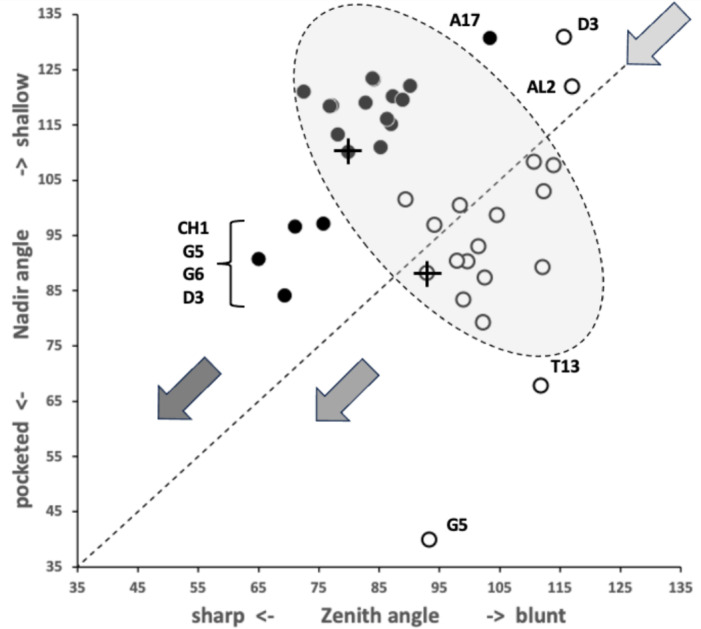
Plot of between species mean angles over individuals for: the first distal asperities i.e., a gullet nadir behind moveable digit tip versus subsequent tooth zenith (= black dots), and; the last proximal asperities i.e., a tooth zenith before the end of the mastication surface at $x_{i_{e}}$ versus the subsequent gullet nadir before the rise of the ascending ramus (= open dots). Grey zone (top left) are bird nest astigmatan species with a Type A like (see Bowman 2024a) arrangement of a typical distal approximately $115^{\circ }$ shallow gullet followed by sharp approximately $80^{\circ }$ first tooth. Note that the surface of a standard rip or cross-cut saw would plot bottom left at [$60^{\circ },60^{\circ }$] (http://www.vintagesaws.com/library/primer/sharp.htm). Grey zone (bottom right) are bird nest astigmatan species with a standard saw-like arrangement proximally i.e., showing typically an approximately $100^{\circ }$ blunter final tooth zenith followed by a final sharper approximately $90^{\circ }$ gullet nadir leading to the end of the mastication surface. Dashed line is unity reference line of slope 1 through zero. Graded grey arrows show ever increasing evolutionary differentiation from bar like moveable digit at $[x=180,y=180]$. Cross = *Acarus gracilis* A4, closest to [$90^{\circ },90^{\circ }$], which are the analogous values of a mosaic-tile cutter. Axes in $^{\circ }$

A marginal between-species plot (Fig. 6) of the species average first (i.e., distal post-tip) tooth and the last proximal tooth zenith angle versus first nadir gullet (i.e., distally post the moveable digit tip) angle and last gullet nadir angle (proximally before rise of the ascending ramus) shows the following.

- Zenith angles are less than the analogous shallow $\approx 170^{\circ }$ angle of the cutting surface of double-sided curved pruning saws (see p 156 Disston & Sons 1918). They overlap with the range of angles for the lateral edges of brick and pointing trowels ($\approx 93-136^{\circ },mean=120^{\circ }$ see Disston & Sons 1918), the tip of a breaking spade (at $\approx 128^{\circ }$, https://www.blackburntools.com/articles/rose-tools-catalog-archives/pdfs/woodings-verona-catalogue-16.pdf), and the edges of tamping bars (at $\approx 108-134^{\circ }$, https://www.blackburntools.com/articles/rose-tools-catalog-archives/pdfs/woodings-verona-catalogue-16.pdf) suggesting that they are adapted to slice through stiff semi-wet granular material (of mortar-like consistency), or clay soil-like material or ‘loose aggregates’. The larger peaks, approximate some wedges (at $116-141^{\circ }$, https://www.blackburntools.com/articles/rose-tools-catalog-archives/pdfs/woodings-verona-catalogue-16.pdf) which function in part like bronze age axes (i.e., chopping and splitting material). Indeed the larger zenith angle species match the classic single bladed Horseman’s battle axe or double bladed Labrys both at $120^{\circ }$ (see https://en.wikipedia.org/wiki/Battle_axe and https://en.wikipedia.org/wiki/Labrys). If indeed bird nest astigmatan teeth act like axes, then their thickness (not just their sharpness Mathieu and Meyer 1997) and how they are ‘hefted’ will determine their efficiencies (Dolfini et al. 2023), i.e., their positioning along and the strengthening of the moveable digit ‘saw-plate’ (see Bowman 2024b). Transverse thickening to strengthen under primate ‘molar’ teeth is an adaptation against torsional stresses Hylander (1979).
- *Lepidoglyphus destructor* G6 and *Glycometrus hugheseae* G3 have the sharpest teeth overall (at $77^{\circ }$ and $80^{\circ }$ overall). The mastication surface of these species may therefore act as graters, surforms or coarse rasps in rapidly removing tough material. These are close to the tooth zenith angles in Table 2 for *Neomys fodiens* the Eurasian Water Shrew (measured from Tiberti and Mori 2016). This is a generalist predator known to favour feeding upon small chitinous aquatic invertebrates but whose weak bite is not strong enough to puncture human skin (Tiberti and Mori 2016).
- Gullet nadir angles are less than the analogous shallow $\approx 160^{\circ }$ angle of the cutting surface of curved pruning saws (see p 154–159 Disston & Sons 1918). They overlap with the range of angles for the lateral edges of brick and pointing trowels ($\approx 93-136^{\circ },mean=120^{\circ }$ see Disston & Sons 1918) and the edges of tamping bars (at $\approx 108-134^{\circ }$, https://www.blackburntools.com/articles/rose-tools-catalog-archives/pdfs/woodings-verona-catalogue-16.pdf) again suggesting that they are adapted to catch upon or ‘scoop’ aggregate granular material. Oiled-skin scrapers (strigils) used for body cleaning are only a little more obtuse (Table 2).
- *Lepidoglyphus destructor* G6, *Glycometrus hugheseae* G3 and *Tyrophagus putrescentiae* T13 have the most pocketed gullets (at $84^{\circ },88^{\circ },93^{\circ }$ overall respectively). These are close to the gullet nadir angles in Table 2 for *Neomys fodiens* the Eurasian Water Shrew (measured from Tiberti and Mori 2016). Again there is a possible commonality for attacking chitinous-like material.
- Distally, typically after the chelal tip there is a shallow ($\approx 115^{\circ }$) gullet in these free-living species followed by a sharp ($\approx 80^{\circ }$) tooth (i.e., they have a Type ‘A’ claw-like ‘hook’ here for tearing foodstuff, Bowman 2024a). Notably *Acarus farris* A17 has a shallower distal gullet and a blunter distal tooth (i.e., it is the most plesiomorphic species - c.f. *Carpoglyphus lactis* in Bowman 2024a, b). By virtue of similar zenith and nadir angle values, *Chortoglyphus arcuatus* CH1, *Dermatophagoides pteronyssinus* D3, *Glycyphagus domesticus* G5 and *Lepidoglyphus destructor* G6 have a sharper more saw-like (‘nibbling’ Type B like) profile distally.
- Proximally, most free-living bird nest astigmatan species have a blunter standard saw-like profile i.e., typically a $\approx 100^{\circ }$ last tooth zenith followed by an $\approx 90^{\circ }$ last gullet nadir (before the rise of the ascending ramus). Notably *Tyrophagus putrescentiae* T13 has a blunt last tooth but with a strongly pocketed proximal gullet confirming the ’herb-stripping scissor’ form (Bowman 2024b). *Glycyphagus domesticus* G5 similarly has an even more sharply formed gullet posteriorly (like a latch, see Bowman 2025b) while *Aleuroglyphus ovatus* AL2 shows an opposite degree of proximal ‘feebleness’. The combination of blunt pre-molars followed by a sharp molar latch-like tooth and final ‘pocket’ just before the ascending ramus is found in the common opossum mandible (Mohamed 2018). Whether mites show the same one-side of the mouth active chewing action (Crompton and Hiiemae 1970) remains to be clarified.
- If the bladed areas of the mastication surface along with the opposable fixed digit acted like 3-point tube cutters (see Anon 1964 p47) then nadir and zenith angles approximating $120^{\circ }$ would be expected. A 4-point tube cutter would match $90^{\circ }$ for both. In either case, tubular fungal hyphae or nematodes could be cut from multiple directions using this composite surface. Such a design is also shown by a tile-cutter (Fig. 6).
- If, rather than cutting and slicing, the mastication surface asperities (along with the fixed digit) are for opposable gripping, then (as explained above) they approximately match the angular geometries of the internal surfaces of hand-held torque wrench socket sets (Table 2).

Taking all this into consideration, across the free-living astigmatan species one could reasonably consider that the first tooth moving posteriorly from the digit tip is indeed ‘the first tooth’ axiomatically, the last ‘gullet’ before the ascending ramus (which must ascend axiomatically posterior of $x_{i_{e}}$) is indeed axiomatically ‘the last gullet’, even if how these features are formed over evolutionary time is unknown. So, given that a moveable digit tip must exist (defined at $[x,y]=[0,0]$) and the end of the mastication surface (at the rise of the ascending ramus, i.e., $x_{i_{e}}$) is measured for all these astigmatans, if there is more than one tooth, the moveable digits can *always* be compared by the zenith angle of their first and last peaks and by comparing the nadir angle of their first and last valleys (no matter what their exact locations along the *L*2*M* axis are). Indeed, given an assumption that extra teeth (and therefore gullets) appear evolutionarily intercalated within the middle region of the mastication surface (like in vertebrate pre-molars), the location of these four features could even be considered as ‘pukka’ geometric morphometric landmarks.

The bird’s nest inhabiting species can then be functionally categorised in several ways:

- by their between and (pooled) within individual variation for their tooth zenith angles,
- by their between and (pooled) within individual variation for their gullet nadir angles,
- by the significance of the difference between their first zenith angle and their last zenith angle within a moveable digit, and
- by the significance of the difference between their first nadir angle and their last nadir angle within a moveable digit
- and by the typical zenith and nadir angle values for any interdigitating asperities, and
- by the detailed features of their intercalated asperities if present

where ‘first’ indicates ‘distal’ (i.e., nearest to the moveable digit tip) and ‘last’ indicates ‘proximal’ (i.e., nearest to the condyle). This is essentially assuming that the earliest derived ‘ancestral’ *apomorphic* form was of a moveable digit tip followed by a gullet, then a tooth distally, plus a final (proximal) tooth and a gullet just before the ascending ramus at the end of the mastication surface i.e., the chela was bidentate (not simply unidentate medially). In other words, mastication surface differentiation over time occurs simultaneously from each end. After all you cannot crush material you have not cut, nor cut material only without crushing it to swallow it via a narrow acarine oesophagus. Further trophic evolutionary changes occurring between this ‘incisor+canine’ module and the final ’molar’ module (using a vertebrate jaw analogy) within the flat or gently gulleted medial region. As such, the appearance of these features might be related to overall digit lengthening or to ‘compression’ of the distal and proximal asperities longitudinally along the *L*2*M* axis (to make ‘free space’) medially? Embryological investigation (or at least comparison across nymphal stages) of digit features may help here in determining if each module is such an integrated functional complex yet is independent of each other (as in shrew mandibles Badyaev and Foresman 2000), and so show mosaic ‘jaw’ evolution.

Fitting regression lines to these angles versus the numbers of teeth or gullets (as appropriate) within each species yields Table 3.

**Table 3:** Linear regression fits (through zero) of total asperity angle (in $^{\circ }$) versus number of asperities per species (genus and species order within family)

Family	Species	Tooth zenith coefficient	*R*^*2*^	Gullet nadir	*R*^*2*^
Acaridae	*Acarus farris* A17	98.654	0.9380	103.460	0.9751
	*Acarus gracilis* A4	86.700	0.9797	93.820	0.9869
	*Acarus immobilis* A1	98.285	0.9917	103.810	0.9956
	*Aleuroglyphus ovatus* AL2	92.983	0.9903	102.910	0.9933
	*Rhizoglyphus robini* R1	97.739	0.9839	104.000	0.9907
	*Thyreophagus entomophagus* TH3	86.694	0.9813	98.572	0.9912
	*Tyrolichus casei* T62	98.900	0.9942	106.820	0.9962
	*Tyrophagus longior* T40	96.308	0.9839	100.460	0.9925
	*Tyrophagus palmarum* T17	100.02	0.9820	105.560	0.9951
	*Tyrophagus palmarum* T32	89.159	0.9841	98.238	0.9913
	*Tyrophagus putrescentiae* T13	97.251	0.9770	93.588	0.9814
	*Tyrophagus similis* T21	94.207	0.9888	99.028	0.9936
	*Tyrophagus similis* T44	88.075	0.9811	96.970	0.9917
Chortoglyphidae	*Chortoglyphus arcuatus* CH1	85.439	0.9827	93.713	0.9912
Glycyphagidae	*Glycometrus hugheseae* G3	82.862	0.9790	88.088	0.9844
	*Glycyphagus domesticus* G5	88.986	0.9727	84.815	0.9751
	*Lepidoglyphus destructor* G6	76.442	0.9900	83.42	0.9916
Pyroglyphidae	*Dermatophagoides pteronyssinus* D3	95.316	0.9853	103.800	0.9923
Suidasidae	*Suidasia pontifica* S5	95.770	0.9777	104.19	0.9865

Coefficients represent typical angle values allowing for within species heterogeneity over all $n=20$ specimens. Low $R^2$ values would indicate a non-linear fit

This confirms that typically both zenith and nadir angles approximate $90^{\circ }$, nadir angles are usually more than zenith angles, and angles in glycyphagids appear smaller than those of acarids. *Tyrophagus* spp. may not form a homogeneous group, this heterogeneity being driven by marked variation within *Tyrophagus palmarum* samples (i.e., between *Tyrophagus palmarum* T17 and *Tyrophagus palmarum* T32). A standard gauge circular saw used for mitreing, crosscutting or ripping timber is not a good design model for the free-living bird nest astigmatan mastication surface (see Table 2). Hand saw design is a better (but not a perfect) match.

Calculating a linear regression line for average tooth zenith angle versus average number of teeth per species suggests (with mild evidence, $R^2=0.1489$) that, as one moves from bird nest habitat astigmatan species to species and as more teeth are effectively intercalated (by evolution) into the mastication tooth row, these teeth have an overall smaller and smaller zenith angle (i.e., as a set they are getting ‘sharper’ by $\approx 5^{\circ }$ less overall per extra tooth, *plot not shown*). However this trend is mainly driven by the multi-dentate *Lepidoglyphus destructor* G6.

**Fig. 7:**
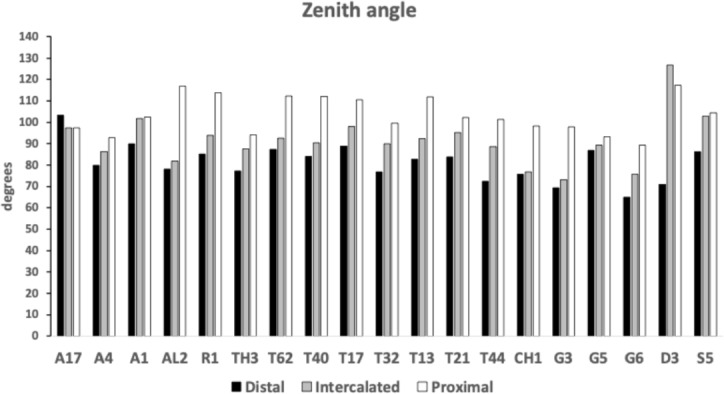
Average zenith angle for distal, proximal and intercalated teeth in bird nest astigmatans. Note progressive blunting distal $\rightarrow$ proximal

Figure 7 shows the species specific averages for tooth zenith angles indicating that intercalated teeth are usually blunter compared to those distally (and sharper than those proximally), suggesting a developmental gradient.

Many bird nest astigmatan individuals sampled have at least one intercalated tooth between the distal and proximal landmark teeth. However, they are rare in *Dermatophagoides pteronyssinus* D3, *Suidasia pontifica* S5 and not very common in *Glycometrus hugheseae* G3, *Tyrophagus similis* T21, *Tyrophagus similis* T44 and *Thyreophagus entomophagus* TH3. Furthermore, *Acarus gracilis* A4 and *Tyrophagus putrescentiae* T13 also appear to have more intercalated teeth than other taxa except the multi-dentate *Glycyphagus domesticus* G5 and *Lepidoglyphus destructor* G6. Extra teeth in general follow progressive blunting (by about an average a $13^{\circ }$ change) posteriorly except in *Glycyphagus domesticus* G5, *Lepidoglyphus destructor* G6 and possibly *Acarus farris* A17 where there is evidence of alternating tooth sharpening (up to a $54^{\circ }$ change) and blunting (up to a $25^{\circ }$ change) as one moves posteriorly. This would fit with the glycyphagids’ mastication surface being distinctly saw-like (especially so in *Lepidoglyphus destructor* G6). Tooth polymorphisms between adult same-sex individuals within species may be useful to avoid competition and exploit different food sources.

Calculating a linear regression line for average gullet nadir angle versus average number of gullets per species gives only poor evidence ($R^2=0.3737$) that as one moves from bird nest habitat astigmatan species to species and more gullets are effectively intercalated (by evolution) into the mastication surface, the gullets have an overall smaller and smaller nadir angle i.e., as a set they are getting more ‘pocketed’ (by $\approx 9^{\circ }$ less overall per extra gullet, *plot not shown*). However, any positive gullet sharpening trend is mainly driven by the multi-gulleted *Acarus gracilis* A4 and *Glycyphagus domesticus* G5.

**Fig. 8:**
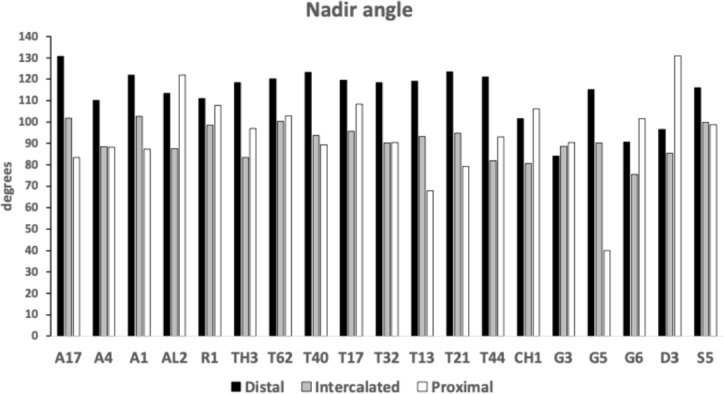
Average nadir angle for distal, proximal and intercalated gullets in bird nest astigmatans. Note sharpening (i.e., more ‘pocketed’) then sometimes blunting distal $\rightarrow$ proximal

Figure 8 shows the species specific averages for gullet nadir angles indicating that intercalated gullets are usually more pocketed than the (first) distal gullet behind the moveable digit tip (*Glycometrus hugheseae* G3 is the only exception). Their relative comparison to the last (proximal) gullet before the rise of the ascending ramus varies across the taxa suggesting that differential evolution may be focused in the proximal moveable digit region, where adductive forces are high. Digit depth (*W*) should accordingly be larger here.

Most bird nest astigmatan species have extra intercalated gullets between the distal and proximal ‘landmark’ gullets. Recall that the number of extra intercalated gullets on a moveable digit must be one more than the number of intercalated teeth, so those species with an extra tooth (see above) will *per force* have an extra gullet ‘automatically’. However, it is not clear what this occasional between individual polymorphism in gullets within species may be useful for. The same unusualness of repeated extra gullets in *Glycyphagus domesticus* G5 and in particular many repeated extra gullets in *Lepidoglyphus destructor* G6 applies amongst the bird nest astigmatan moveable digit profiles similar to that pointed out above for teeth.

Invariably, bar the equivocal *Dermatophagoides pteronyssinus* D3 and *Rhizoglyphus robini* R1, the first (most anterior) extra gullet is confirmed as sharper (i.e., more pocketed compared to the gullet posterior of the moveable digit tip, by $20^{\circ }$ on average across species). The second, third and fourth extra intercalated gullets (if present) paint a more complicated picture of variable nadir angle changes across the species (covering a $\approx \pm 18^{\circ }$ change in general). For individuals from multi-gulleted species (like *Acarus immobilis* A1, *Acarus farris* A17, *Acarus gracilis* A4, *Tyrophagus putrescentiae* T13, *Tyrophagus longior* T40 and especially *Glycyphagus domesticus* G5 and the markedly multi-dentate *Lepidoglyphus destructor* G6), there is evidence of alternating sharpening (up to a $66^{\circ }$ change) and blunting (up to a $49^{\circ }$ change) of gullets as one moves posteriorly. This is a classic saw design motif.

Strong progressive sharpening (i.e., becoming more and more pocketed posteriorly) in the extra intercalated gullets within the mastication surface, after an initial mild comparative blunting at the third gullet (by a $7-11^{\circ }$ change) occurs for *Glycyphagus domesticus* G5 (with a $17-66^{\circ }$ change) and in *Tyrophagus putrescentiae* T13 (with a $6-32^{\circ }$ change). This similar motif to the posterior latch-like pocketing (reported above) must have a common function for these two mites trophically. Could it have a ‘snapping’ advantage?

**Fig. 9:**
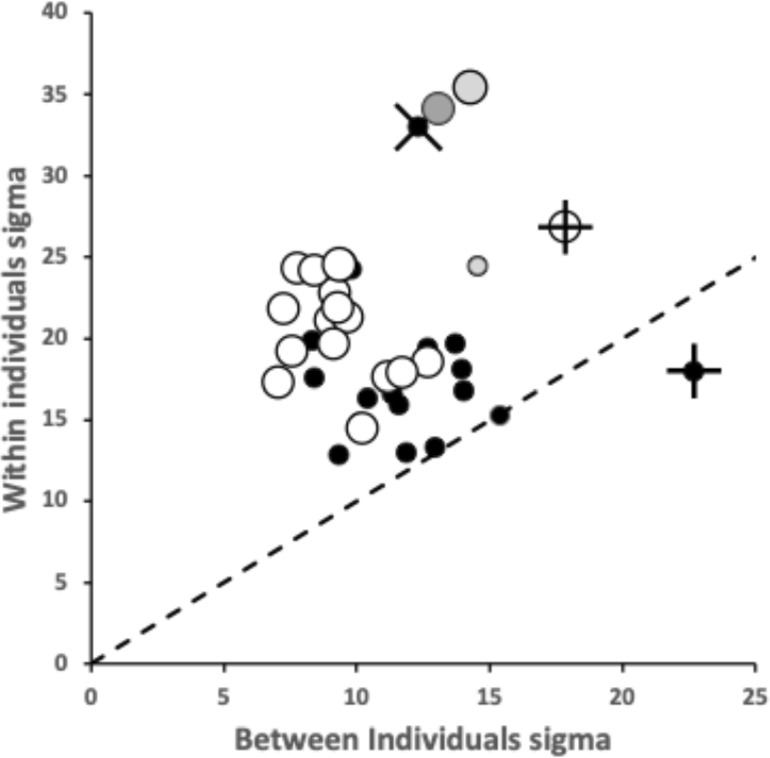
Within individual (= between location) standard deviation ($\sigma$) plots for tooth zenith angles (= small black dots) and gullet nadir angles (= large open dots) for bird nest habitat species. Overlay of X = *Dermatophagoides pteronyssinus* *Dermatophagoides pteronyssinus* D3 zenith angle. Overlay of + = *Acarus farris* A17. Dark grey large open dot is gullet nadir angle for *Glycyphagus domesticus* G5. Pale grey dots = *Tyrophagus putrescentiae*. Dashed line is unity. Axes in $^{\circ }$

Within and between individual variation across the species of (all) tooth zenith and gullet nadir angles is shown in Fig. 9. In general, variation for tooth zenith angles and gullet nadir angles are broadly similar, although gullet nadirs are about twice more variable within species than tooth zeniths usually are, and about a third less variable between species. Moveable digit gullets thus offer the opportunity for polymorphisms in feeding style within and between mite taxa, just as found for different composite saw types. ‘Debris’ or ‘detritus’ handling is an evolutionary focus for free-living bird nest astigmatans.

This between individual tooth zenith angle variation being of the same order as the within individual (= between location) tooth zenith angle variation suggests that teeth are all similar in zenith angle whenever present but their actual height and location of course can change. However, in general the gullet nadir angles varying more along the mastication surface suggest that in that respect the species exhibit a degree of convergent diversity i.e., they are are like multiple competing jewellery shops in the same town each with a wider variety of jewellery items within them (of possible different diversity) than their average stock type suggests. In other words, trophic evolution may be more focusing on gullet variation once teeth are present. Efficient ‘detritus’ handling matters!

For each ‘cloud’ of points, a decrease in *y*-axis values (in Fig. 9) indicates a more homogeneously designed saw-like profile within a species. *Acarus farris* A17 is a noticeably heterogeneous species for both angles. *Dermatophagoides pteronyssinus* D3 has noticeably high within species variation for tooth zenith angles ($\equiv$ heterodonty). *Glycyphagus domesticus* G5 and *Tyrophagus putrescentiae* T13 have noticeably highly variable within individual gullet nadirs. The latter *Tyrophagus putrescentiae* T13 taxon also being a possible heterodontous outlier for tooth zenith variation.

### Analysing saw-types

Formally restricting the data to just the first (most distal) and last (most proximal) angles so as to carry out a split plot analysis of the data gives Table 4. For this set of data, peaks overall are characterised as having zenith angles on average of $92.45^{\circ }$. The first tooth (i.e. posterior of the moveable digit tip) and the last tooth (i.e., that before the rise of the ascending ramus) are characterised as having zenith angles on average of $81.29^{\circ }$ and $103.61^{\circ }$ respectively. For this set of data, gullets overall are characterised as having nadir angles on average of $103.41^{\circ }$. The first gullet (i.e. that posterior of the moveable digit tip) and the last gullet (i.e., that before the rise of the ascending ramus) are characterised as having nadir angles on average of $113.19^{\circ }$ and $93.63^{\circ }$respectively.

**Table 4:** Observed mean angle (in $^{\circ }$) of first (distal) and last (proximal) mastication surface teeth and gullet scored by species (alphabetically within family and genus)

Family	Species	Tooth zenith distal	Tooth zenith proximal	Gullet nadir distal	Gullet nadir proximal
Acaridae	*Acarus farris* A17	103.34	98.95	130.66	83.43
	*Acarus gracilis* A4	79.90	92.88	110.09	88.18
	*Acarus immobilis* A1	90.08	102.55	122.07	87.35
	*Aleuroglyphus ovatus* AL2	78.12	116.96	113.32	121.99
	*Rhizoglyphus robini* R1	85.25	113.94	110.95	107.71
	*Thyreophagus entomophagus* TH3	77.20	94.17	118.54	96.91
	*Tyrolichus casei* T62	87.27	112.29	120.23	103.00
	*Tyrophagus longior* T40	84.05	112.05	123.30	89.29
	*Tyrophagus palmarum* T17	88.91	110.66	119.59	108.34
	*Tyrophagus palmarum* T32	76.80	99.61	118.42	90.34
	*Tyrophagus putrescentiae* T13	82.75	111.79	119.04	67.81
	*Tyrophagus similis* T21	83.91	102.19	123.51	79.33
	*Tyrophagus similis* T44	72.43	101.41	121.04	93.01
Chortoglyphidae	*Chortoglyphus arcuatus* CH1	75.78	98.37	97.12	100.51
Glycyphagidae	*Glycometrus hugheseae* G3	69.34	97.89	84.10	90.47
	*Glycyphagus domesticus* G5	86.97	93.28	115.15	39.97
	*Lepidoglyphus destructor* G6	65.03	89.39	90.72	101.59
Pyroglyphidae	*Dermatophagoides pteronyssinus* D3	71.00	115.69	96.64	130.96
Suidasidae	*Suidasia pontifica* S5	86.29	104.48	116.15	98.75

**Fig. 10:**
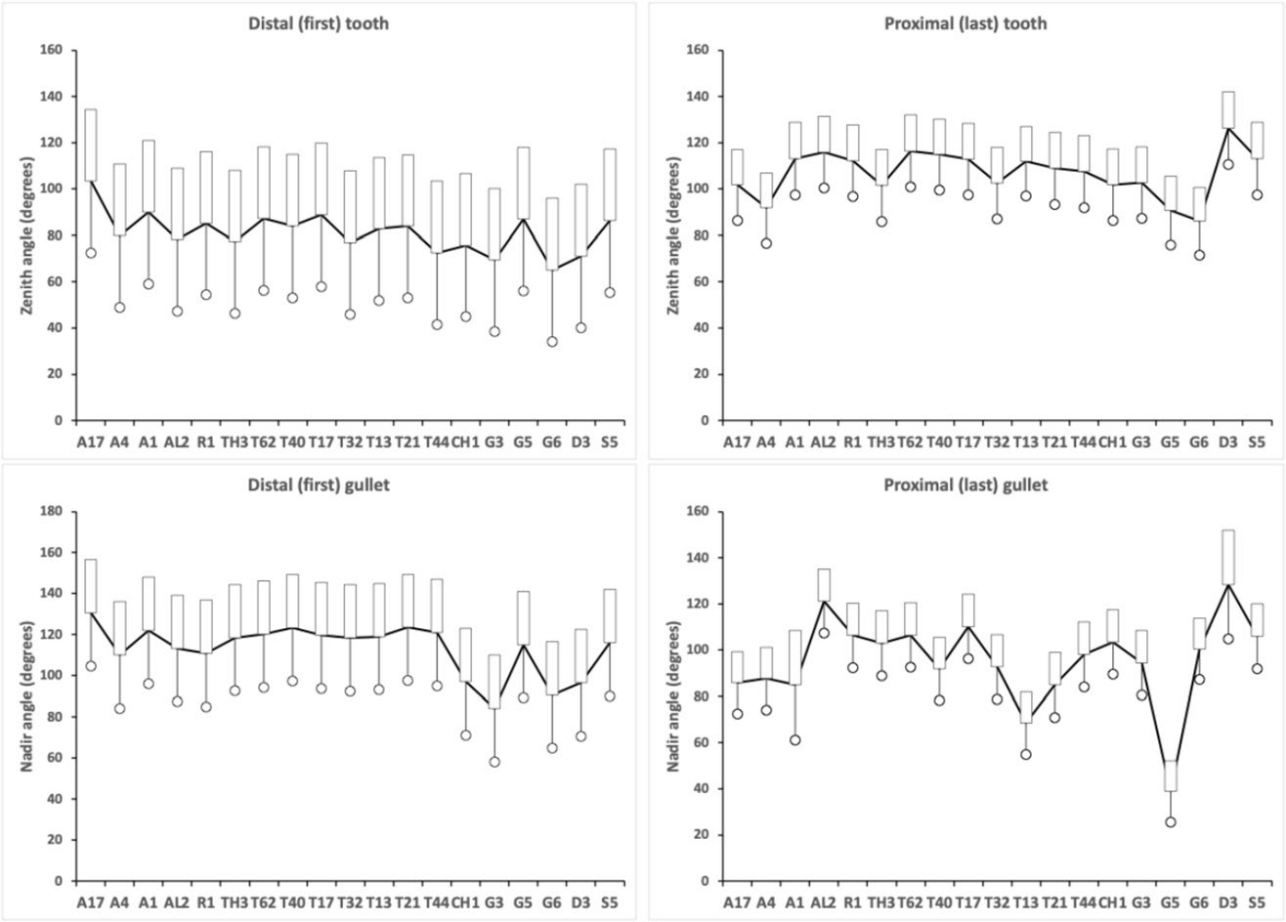
Fitted means (in $^{\circ }$) and $95\%$ confidence intervals from split-plot analysis of asperity zenith and nadir angles. Bar = upper confidence interval. Dot and line lower confidence interval. Top row = tooth zenith angle. Bottom row = gullet nadir angle. Left sub-panel = distal (first) asperity. Right sub-panel = proximal (last) asperity. Species ordered in families and then alphabetically by genus and species (with line through means to highlight differences)

Statistically, this shows dramatic differences within and between species (although now the two samples of *Tyrophagus palmarum* show similar trends, showing that their earlier between sample heterogeneity was driven by ‘extra’ intercalated teeth and gullets). Performing an overall split-plot linear mixed effects analysis confirms different species approximate different tools. For tooth zenith angles the difference between the distal and proximal angles varies across species (or equivalently the difference between species varies across the two locations) significantly $F_{(18,719)}=3.9994, p<0.0001$. For gullet nadir angles the difference between the distal and proximal angles varies across species (or equivalently the difference between species varies across the two locations) significantly $F_{(18,719)}=16.5910, p<0.0001$. The estimates for each effect are shown in Fig. 10.

A clearly different pattern of gullet differentiation between species is confirmed. Mapping this to carpentry - a gullet on a wood saw is there to clear debris and to stop the blade getting clogged and getting stuck in material. One has to cut through individual wood fibres when using a crosscut saw so the ‘chopping’ blades are close together $\Rightarrow$ small gullets. Debris *per force* is fine (i.e., detrital) dust therefore. When cutting with the grain, one is trying to split the wood like an axe or chisel (which requires less work and generates less friction). So a composite blade has a series of chisel blades arranged not too close together $\Rightarrow$ large gullets. This allows the wood to be ripped before the next cutting surface is applied (especially if the wood is green, see diagram in http://www.vintagesaws.com/library/primer/sharp.html). Alternating gullet sizes gives a general purpose saw. So, regions of an astigmatan moveable digit with small gullets suggest a fine cross-cutting action is deployed there by the cheliceral chela, regions with large gullets a ripping action (irrespective of the teeth geometry). Different bird nest astigmatans thus handle different foodstuff detritus differently.

### Analysing ‘hook’ types

Gullet nadir angles in Fig. 10 generally approximate the $100^{\circ }$ mean values seen in the claw angles of predatory, climbing or perching birds (Pike and Maitland 2004). However, that for the proximal (last) gullet in *Glycyphagus domesticus* G5 is low like the claw angle of ground-dwelling birds, suggesting a very different function. The general pattern in astigmatans sits between the minimum to maximum inner surface ‘hook ratios’ of bat claws (Waring and Essah 2016, which map to $\approx 80-113^{\circ }$ respectively). The gullet nadir angles also are enclosed by the minimum ($82^{\circ }$) to maximum ($173^{\circ }$) values for turtle claw ‘curvature angle’ over all the listed values in the supplementary information for Tulli et al. (2022). A common grasping role is thus indicated. This is consilient with the ’perching’ role for the matching design of fossil drepanosaurid reptile claws (Spielmann et al. 2006).

Indeed Thomson and Motani (2021) (their Table 1) give a useful sub-categorisation of vertebrate claws into: Amplectorial (Grasping); Cursorial (Running/hopping); Gryporial (Hook-and-pull digging); Scalporial (Scratch-digging); Scansorial (Climbing); Suspensorial (Hanging); Tenasorial (Grappling); and Generalist (Multipurpose) forms. Mean ventral claw curvature angles were quoted for Scalporial, Cursorial and Gryporial of around $40^{\circ }$. Mean ventral claw curvature angles of $\approx 60-80^{\circ }$ were quoted for Generalist and Suspensorial forms. Values $\approx 100^{\circ }$ were given for Scansorial and Amplectorial functioning claws. Finally, Tenasorial claws showed $\approx 140^{\circ }$.

This would infer that the proximal gullet of *Glycyphagus domesticus* G5 is adapted to facilitate at least Scratch-digging and Hook-and-pull digging into foodstuff. This is like the claw digging action of pangolins, meerkats, aardvarks, most anteaters and armadillos (Thomson and Motani 2021). While the majority of bird nest astigmatans have multipurpose moveable digit gullets seemingly adapted for Hanging onto, ‘Climbing over’ and Grasping foodstuff asperities (or prey, secton 4.4 in Thomson and Motani 2021). This action is like the claws of sloths, squirrels, climbing birds, perching birds, and most predatory birds (Thomson and Motani 2021). Only for *Dermatophagoides pteronyssinus* D3 proximally do moveable digit gullets appear to favour a (prey) active Grappling function like the claws of felids, the osprey and the Seriema (see Thomson and Motani 2021).

### ATB versus FT teeth

Both ‘likely to be alternate top bevel’ (ATB) faceted teeth and ‘likely to be flat-top’ (FT) faceted teeth types were scored. Even if not deployed in a sawing action, such asperities on chela occlusion would act like hammers.

Would a follow-up SEM study show any astigmatan tooth upper surfaces resembling the “... distinctive leaf shape with a primary ridge running down the middle...” found on ceratopsid teeth (see Fig 20 in Loewen et al. 2024) “... adapted to processing high-fibre plant material...” (https://en.wikipedia.org/wiki/Ceratopsidae.

ATB teeth would indent material (like multiple small chisel blades) as well as simply crush it. Such impressions in food material could be looked for in follow-up SEM work. Also one could examine the facets of such teeth to see if they approximate the $25^{\circ }$ sharpening angle of wood chisels (and plane blades, https://robcosman.com/pages/secrets-of-hand-plane-sharpening), or if two-sided that they possess an $\approx 50^{\circ }$ zenith angle. Indeed, if micro-peaks were set at $12-30^{\circ }$ with respect to foodstuffs moving laterally over them so as to act like rasps or graters, then that would infer micro-gullets in between them of $120-156^{\circ }$. Matching astigmatan species asperities to abrading file types (known to have different uses, https://en.wikipedia.org/wiki/File_(tool), and consequent micro-feature orientation) using SEM could be useful.

All species studied carried both tooth forms on their moveable digits in variable numbers within and between species. Across all taxa, ATB are characterised as having zenith angles on average of $84.2^{\circ }$ (range: $73.2-95.4^{\circ }$). Across all taxa, FT teeth are characterised as having flatter zenith angles on average of $102.9^{\circ }$ with an almost non-overlapping range: $92.7-116.8^{\circ }$). *Tyrophagus putrescentiae* T13 has the flattest zenith angle on average for its ATB teeth ($95.4^{\circ }$), *Glycometrus hugheseae* G3 and *Lepidoglyphus destructor* G6 the sharpest (ATB teeth zenith = $73.2^{\circ }$ and $73.9^{\circ }$on average respectively). For FT teeth *Acarus farris* A17, *Glycometrus hugheseae* G3 and *Dermatophagoides pteronyssinus* D3 have noticeably flatter zenith angles on average at 116.8°, 114.6° and $114.1^{\circ }$ respectively. Follow-up scanning electron microscope work is indicated to clarify the exact detail of species-specific patterns detected. Even though validated and accepted characterisations of what makes a teeth ATB or FT need to be developed, taxonomic acarologists could perhaps begin to include in their descriptions a subjective assessment of the apparent sharpness of cheliceral teeth (or even try to measure zenith angles) on type specimens.

### What about peak and valley spacing?

**Fig. 11:**
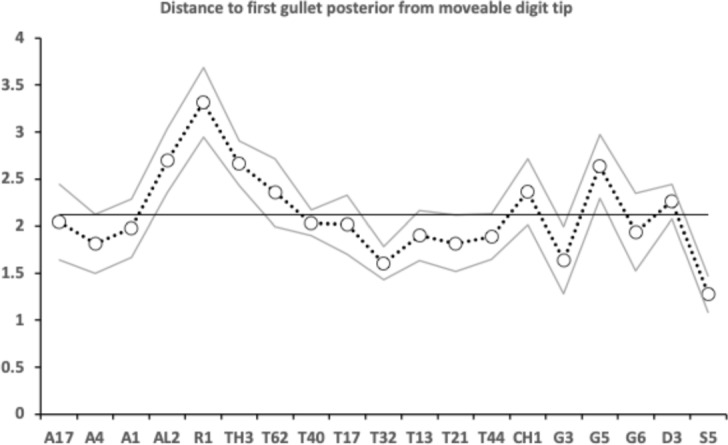
Observed means (open dots) for each taxon (average over individuals) for distance of first gullet (g1) posterior of moveable digit tip in $\mu$m. Grey lines upper and lower 95% confidence interval for that taxon. Solid horizontal line = overall mean over species. Means joined by dashed line for clarity. *Rhizoglyphus robini* R1 has a noticeably elevated value. *Suidasia pontifica* S5 a diminished value

Inter-peak distances were referenced from the previous peak with the moveable digit tip (zeroth peak) being at the origin (Fig. 1, and Technical Appendix). Inter-valley distances were similarly measured from the previous valley. However, that leaves knowing where along *L*2*M* the first gullet is. This is displayed in Fig. 11. *Rhizoglyphus robini* R1 has a comparatively particularly long distance to the first gullet posterior of the digit tip, while *Suidasia pontifica* S5 has the shortest. However this is proportionate to body size (regression through zero with $IL, R^{2}=0.9587$, *plot not shown*), suggesting a common intrinsic basis.

**Fig. 12:**
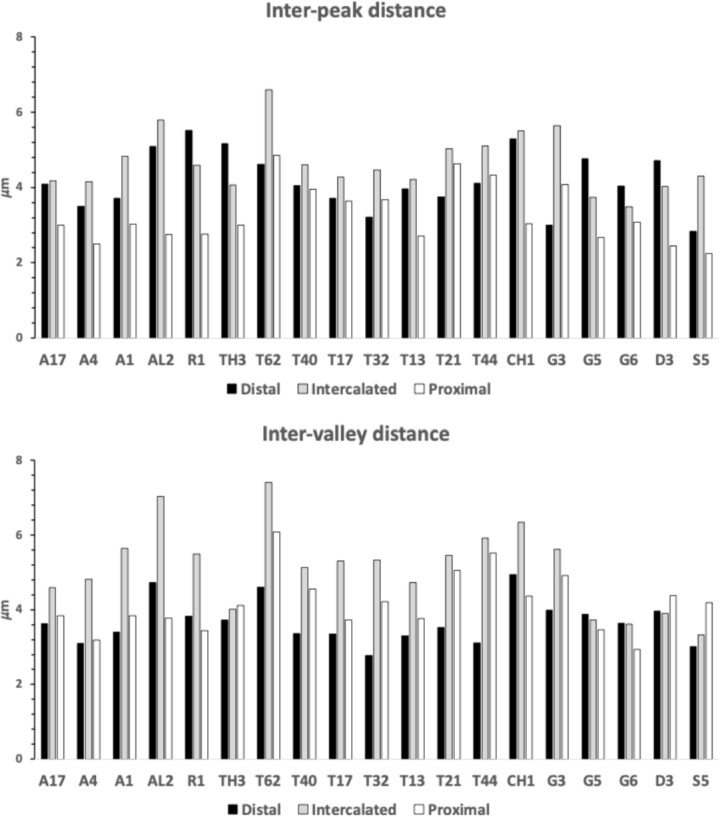
Observed means for each taxon (average over individuals) for distance between subsequent peaks (inter-tooth) and distances between subsequent valleys (inter-gullet) counting from the tip posteriorly. Digit tip is zeroth peak. Back bars = distal, grey bars = intercalated asperities, white bars = proximal

Figure 12 shows the averages for each taxa for inter-peak and inter-gullet distances in $\mu$m distal to the condyle, proximal to the condyle and for any asperities intercalated between. The overall average inter-tooth distance was $4.1\mu$m, the overall average inter-gullet distance was $4.2\mu$m. Average inter-tooth distances were distally $4.2\mu$m, proximally $4.6\mu$m, and for intercalated asperities $=3.3\mu$m. Average inter-gullet distances were distally $3.7\mu$m, $4.2\mu$m proximally, and for intercalated asperities $=5.0\mu$m. Indeed, although the detailed pattern varies within each species inter-tooth and inter-gullet distances are highly correlated ($R^2=0.9820$ distal, $R^2=0.9913$ intercalated, $R^2=0.9801$ proximal, across species *plots not shown*) suggesting that both are generated in concert by the same developmental mechanism. There is mild evidence that species with longer distal inter-tooth distances (i.e., where the initial gap anteriorly behind the digit tip is big) have proportionately lower inter-gullet distances distally. In other words a long ‘hook’ distally is promptly followed by multiple teeth (= an ‘enhanced Type A-like’ design, Bowman 2024a). Examples here are *Rhizoglyphus robini* R1 and *Thyreophagus entomophagus* TH3. The opposite occurs proximally, i.e., an extended inter-tooth distance before the end of the mastication surface (at $x_{i_{e}}$) in a species is matched by an even longer inter-gullet distance anterior of it thus showing its enhanced stretched-out ‘Type B like’ nature (Bowman 2024a) even allowing for any proximal asperities. Examples here are *Dermatophagoides pteronyssinus* D3 and *Suidasia pontifica* S5.

For each taxon, extra teeth are inserted on average at an ever-diminishing inter-peak distance as one moves posteriorly along the moveable digit away from the tip (with the exception of the third intercalated posterior tooth in *Acarus farris* A17 and *Acarus gracilis* A4 when present). Similarly, for each taxon, extra gullets are inserted on average at an ever-diminishing inter-valley distance as one moves posteriorly along the moveable digit away from the tip (with the exception of the second intercalated posterior gullet in *Acarus farris* A17 and *Glycyphagus domesticus* G5). The mastication surface in bird nest free-living astigmatans has an asymmetrically design axis orientation anterior$\rightarrow$posterior (rather than posterior$\rightarrow$anterior or symmetrically from both ends towards its centre).

### Rising and falling $R_{t}$

**Fig. 13:**
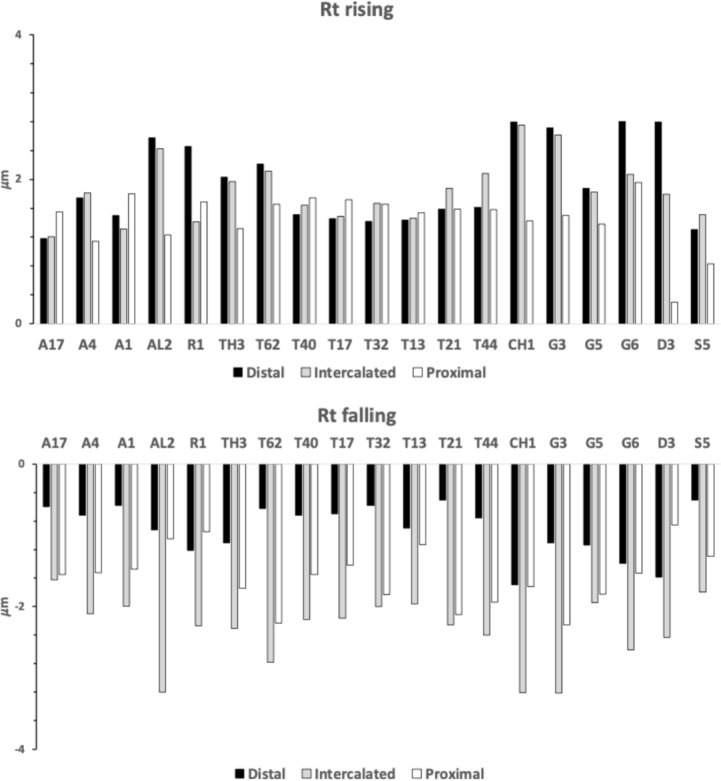
Observed means for each taxon (average over individuals) for total vertical distance between gullet to subsequent tooth (rising $R_{t}$) Upper, or between tooth and subsequent gullet (falling $R_{t}$) Lower, counting from the tip ($i=0$) posteriorly. Black bars = distal ($i=1$), grey bars = intercalated asperities ($i\ne (1,last)$), white bars = proximal ($i=last$)

Working anterior-posteriorly along the moveable digit, the average total mastication surface deformation rising from the *i*th (current) gullet to the (subsequent) *i*th tooth (i.e., $R_{t}$ rising) is shown in Fig. 13 Upper for each region (distal $\Rightarrow i=1$, intercalated $\Rightarrow i\ne (1,last)$, proximal $\Rightarrow i=last$). Similarly working anterior-posteriorly along the moveable digit, the average total mastication surface deformation falling from the *i*th (current) tooth to the (subsequent) *i*th gullet (i.e., $R_{t}$ falling) is shown in Fig. 13 Lower for each region (distal $\Rightarrow i=1$, intercalated $\Rightarrow i\ne (1,last)$, proximal $\Rightarrow i=last$). Average effective penetration into foodstuff is $1.8\mu$m. Depending upon location, species and where food is placed along the digit, effective penetration of food-stuff could be on average as low as $0.3\mu$m (*Dermatophagoides pteronyssinus* *D*3) or as high as $3.2\mu$m (in *Aleuroglyphus ovatus* AL2). This penetration scales well with body size ($R^{2}=0.9683$ for regression through zero with *IL*). Tiny asperities are typical of kitchen, bone and dehorning saws (see Disston & Sons 1918). They also are found in metal-working hacksaws. Large asperities are found on coarse rip-saws. $R_{t}$ values are in the region of half the average particle size (above the mean, $S_{a}$) values of ‘ultra fine’ 2000-grit sandpapers (used for final sanding and polishing of thick wood and metal finishes and bare metal).

Species fall into different patterns of the relative magnitudes of each $R_{t}$ rising and $R_{t}$ falling. A large initial (distal) tooth rise is present in *Aleuroglyphus ovatus* AL2, *Rhizoglyphus robini* R1, *Chortoglyphus arcuatus* CH1, *Glycometrus hugheseae* G3, *Lepidoglyphus destructor* G6 and *Dermatophagoides pteronyssinus* D3 giving a strong leading edge on cheliceral protrusion. This is preceded by a large post-tip drop in *Chortoglyphus arcuatus* CH1 giving a strong hook. Intercalated teeth with large rises are found in *Aleuroglyphus ovatus* AL2, *Chortoglyphus arcuatus* CH1 and *Glycometrus hugheseae* G3 which all features large falls too like a coarse rasp. Only *Dermatophagoides pteronyssinus* D3 has unusually rising proximal teeth (being minute). Proximally falling $fR_{t}$ values are variable across taxa.

**Fig. 14:**
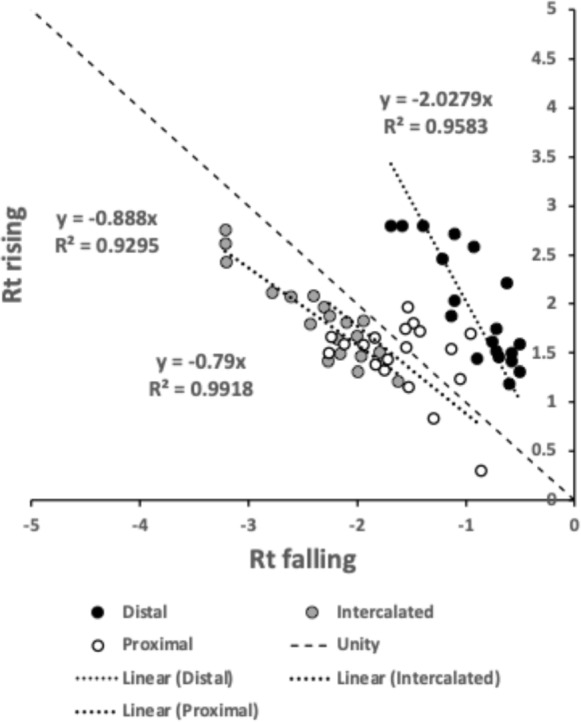
Relationship of rising and falling $R_{t}$ values across taxa and moveable digit regions

Rising ($rR_{t}$) values from the depth of a preceding gullet to the peak of a subsequent tooth is well correlated across taxa with the falling ($fR_{t}$) values from a preceding tooth peak to a subsequent following gullet depth for bird nest astigmatans overall (Fig. 14). Only in the distal region do rising values noticeably exceed falling ones, pointing to the unusualness of the first post tip gullet with respect to the first distal tooth in all taxa (i.e., a design matching a Type A distal claw-like hook, see Bowman 2024a). As one moves posteriorly along the mastication surface, intercalated and proximal gullets appear to be drifting ventrally (*plot not shown*).

**Fig. 15:**
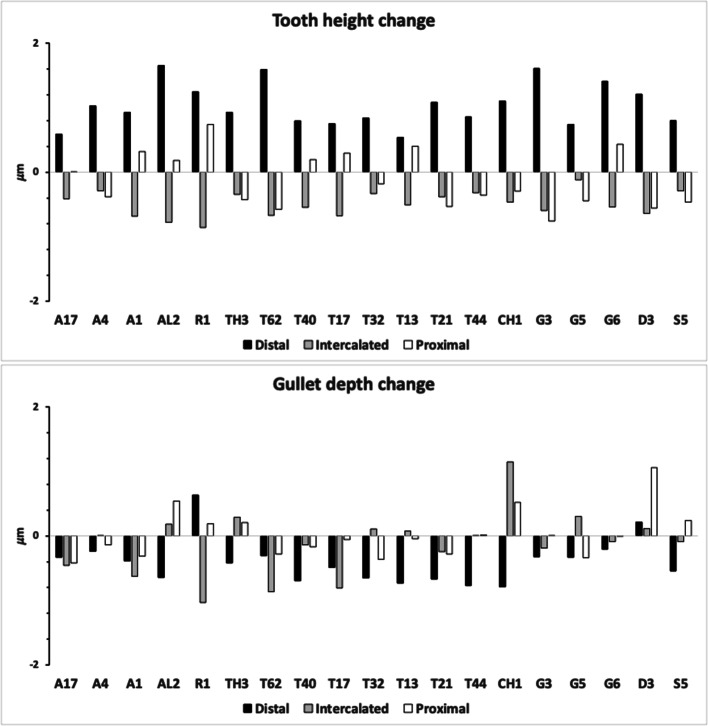
Observed means for each taxon (average over individuals) for change in tooth height Upper, or change in gullet depth Lower, counting from the tip ($j=0$) posteriorly. Back bars = distal ($j=1$), grey bars = intercalated asperities ($j\ne (1,last)$), white bars = proximal ($j=last$)

Adding $rR_{t}$ rising and $fR_{t}$ falling values per *j*th location and averaging per species over individuals gives ‘tooth height change’ (for $j=1$ previous tooth = moveable digit tip; for $j=last$ subsequent tooth being at $x_{i{e}}$) as one moves anterior-posteriorly along the digit. If lagged, then $rR_{t}(j)$ rising $+fR_{t}(j+1)$ falling values and averaging per species over individuals gives ‘gullet depth change’ as one moves anterior-posteriorly along the digit. Both of which can be regionalised for distal, intercalated and proximal regions of the asperities. Figure 15 shows this data. Generally intercalated and proximal teeth decline in height from the previous tooth while distal ones increase (noticeably for *Aleuroglyphus ovatus* AL2, *Tyrolichus casei* T62, *Glycometrus hugheseae* G3 and probably *Lepidoglyphus destructor* G6). In other words, teeth after the distal first one (=‘incisor/canine’) invariably (bar *Rhizoglyphus robini* R1 proximally) decline in height (with respect to the *L*2*M* reference axis). Whichever region that they are in, gullets deepen from the previous one except occasionally not in *Aleuroglyphus ovatus* AL2, *Rhizoglyphus robini* R1, *Chortoglyphus arcuatus* CH1, *Dermatophagoides pteronyssinus* D3 and possibly not in *Thyreophagus entomophagus* TH3. In other words gullets usually deepen anterior-posteriorly in bird nest astigmatans. Overall *Rhizoglyphus robini* R1 looks like a distinctive design from all other bird nest astigmatans.

**Fig. 16:**
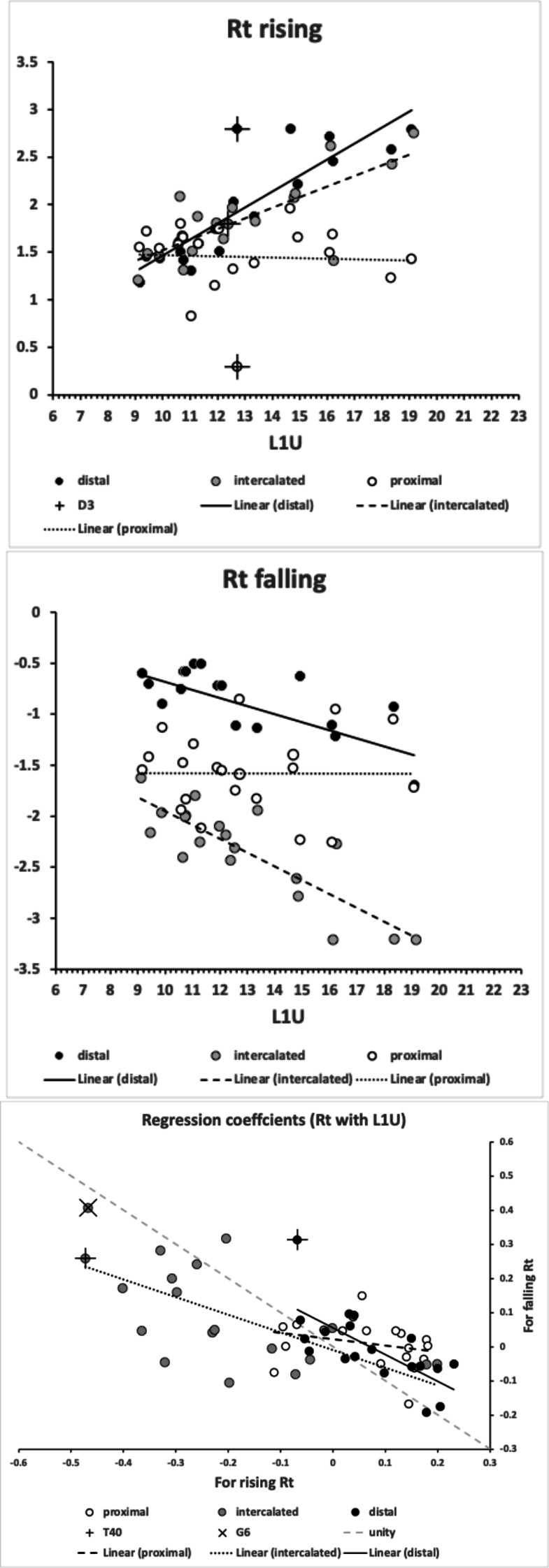
Average $R_{t}$ values per bird nest astigmatan taxon by moveable digit region. Upper, ‘rising’ - from preceding gullet to following tooth. Middle, ‘falling’ - from preceding tooth to following gullet. Lower, within species regression coefficients for $R_{t}$ falling versus $R_{t}$ rising with adductive input lever arm *L*1*U* for each moveable digit region

Do $R_{t}$ values scale (not just with *IL* but) with the adductive input lever arm *L*1*U* i.e, a more powerfully designed chela has a bigger mastication surface rise and fall? Note that the output adductive force (*F*2*AV* from Bowman 2021) is highly positively correlated with the *L*1*U* values from this study herein ($r=0.950$) so *L*1*U* as a height is also a reasonable measure of ‘chelicerisation’ or ‘robustification’ in bird nest astigmatans (just as head height is associated with greater bite force in lizards Herrel and Holanova 2008). Figure 16 (upper and middle) shows across taxa that as expected, $R_{t}$ values (both rising and falling) increase in absolute size with chelal input force lever arm *L*1*U* length ($R^2$ range from 0.4052 to 0.7363) except for the proximal moveable digit surface region. Here asperities remain similar in absolute size i.e., they get diminished relative to vertically orientated cheliceral growth. Just like the moveable digit tip region is distinct with respect to adaptive lengthening in bee-hive astigmatans (see *Carpoglyphus lactis* in Bowman 2024b), the proximal ‘pre-molar/molar’ region near the ascending ramus is also particular with respect to vertically orientated chelal evolutionary changes in bird nest astigmatans. *Dermatophagoides pteronyssinus* D3 proximally is especially unusual. Indeed within species regressions for rising and falling $R_{t}$ values scale similarly with *L*1*U* irrespective of region and species (Fig. 16 lower) although the various scales reflect the differences between species in their response to chelicerisation. *Tyrophagus longior* T40 for sure proximally (at least) is rather unusual with a marked difference in scaling for the final drop (i.e., the proximal ‘pocket’ gets much bigger on increased cheliceration) versus the final rise in posterior features. *Lepidoglyphus destructor* G6 is the most extreme taxon for intercalated scaling. Indeed intercalated teeth are where evolutionary chelicerisation appears to have the biggest impact in increasing or decreasing the size of asperities.

**Fig. 17:**
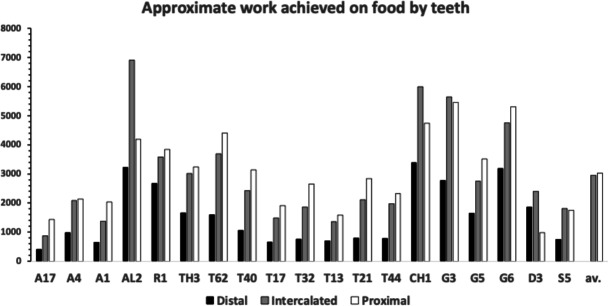
Approximate work achieved by teeth penetrating into foodstuff (by taxon and moveable digit region, based upon mean of rising $rR_{t}$ and absolute value of falling $fR_{t}$ with *F*2*AV* from Bowman 2021, *see text*). av = mean over all taxa

$R_{t}$ is an indicator of penetration into foodstuff. Therefore the distance the tip of a typical moveable digit tooth travels before a typical moveable digit gullet (in that region) prevents the digit moving further into the foodstuff by force applied (i.e., $R_{t}$ multiplied by occlusive force *F*2) could be considered as the ‘work achieved’ by that region of the moveable digit. The location of the tooth along the reference *L*2*M* axis determines the exact force at that point (in a hyperbolic fashion Bowman 2024a). However, if one assumes that the relationship of the proximal, intercalated and distal regions‘ positions are broadly similar across the taxa at $\approx 0.5,0.75,1$ respectively times *L*2*M* (and thus the adductive force is $2, \frac{4}{3},1$ times respectively the tip occlusal force *F*2*AV* values from Bowman 2021), then comparisons of the approximate work achieved by typical teeth across taxa and regions can be made using the mean of the rising and falling $R_{t}$ values (Fig. 17). This shows that as expected, work achieved increases posteriorly. Also that *Aleuroglyphus ovatus* AL2, *Chortoglyphus arcuatus* CH1, *Glycometrus hugheseae* G3, *Lepidoglyphus destructor* G6 and possibly *Rhizoglyphus robini* R1 and *Tyrolichus casei* T62 have much higher values compared to other taxa in line with both a larger body size ($R^2=0.1086$ for intercalated teeth, *plot versus IL not shown*) and increased cheliceration ($R^2=0.7189$ for intercalated teeth, *plot versus CHI not shown*).

**Fig. 18:**
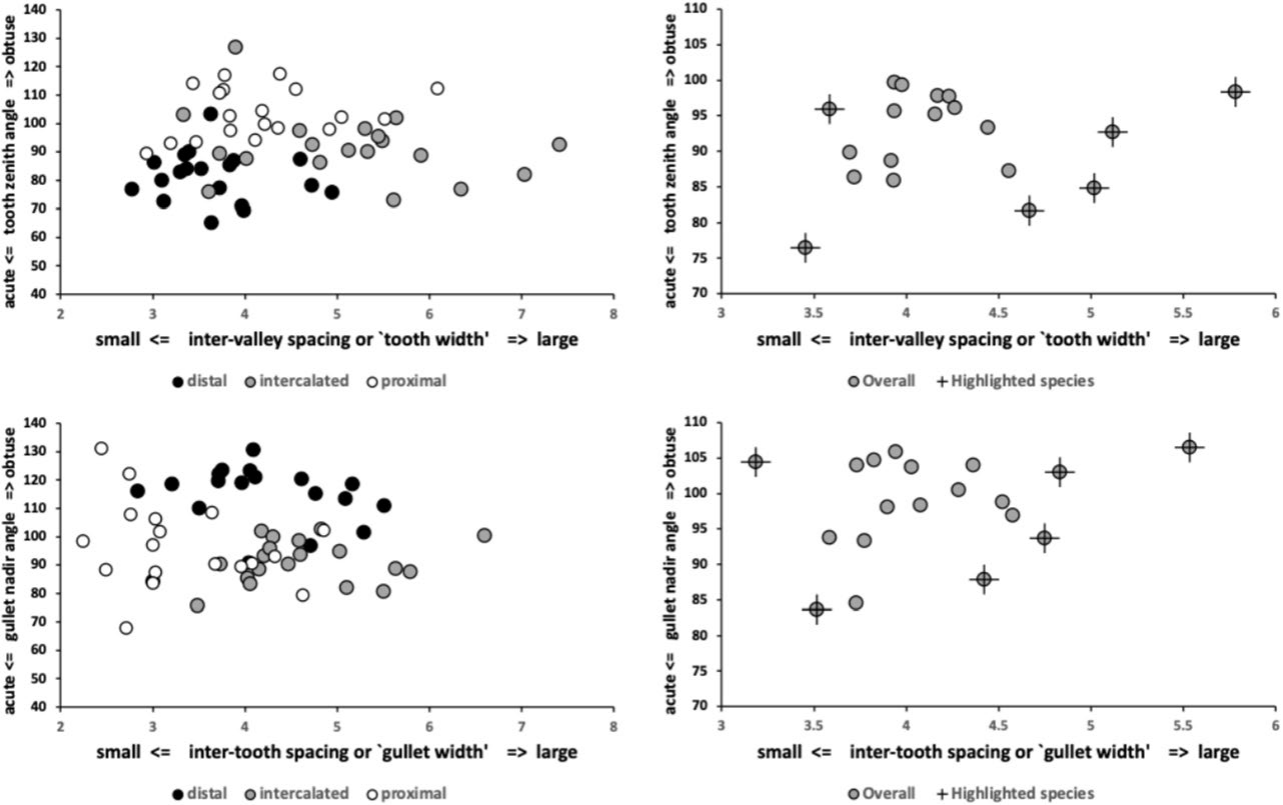
Sharper teeth and more pocketed gullets tend to be closer together in bird nest astigmatan moveable digits. Dots = data. Plus = highlighted species (*see text*). Upper: Relationship of average tooth zenith angle versus estimated ‘tooth width’. Left categorised by mastication surface region. Right overall, where highlighted dots right to left are *Tyrolichus casei* T62, *Aleuroglyphus ovatus* AL2, *Chortoglyphus arcuatus* CH1, *Glycometrus hugheseae* G3 and the most extremum bottom left dot is *Lepidoglyphus destructor* G6 with *Suidasia pontifica* S5 as the most top left extremum. Lower: Relationship of average gullet nadir angle versus estimated ‘gullet width’. Left categorised by mastication surface region. Right overall, where highlighted dots right to left are *Tyrolichus casei* T62, *Aleuroglyphus ovatus* AL2, *Chortoglyphus arcuatus* CH1, *Glycometrus hugheseae* G3 and the most extremum bottom left dot is *Lepidoglyphus destructor* G6 with *Suidasia pontifica* S5 as the most top left extremum. *Glycyphagus domesticus* G5 has now joined the bottom left set

Another way to look at this is to examine the relationship of inter-valley spacing with tooth zenith angle categorised by the mastication surface region being distal, intercalated or proximal (Fig. 18 Upper). Inter-valley (= inter-gullet) spacing estimates the tooth width in that region. Most taxa have modest size teeth overall of $\approx 90^{\circ }$. The moveable digit mastication surface of *Lepidoglyphus destructor* G6 is like a shrew mandible enabled to grip slippery foodstuffs. The relative vertical ordering in the left hand upper sub-panel confirms the general flattening of teeth zenith angles as one moves anterior-posteriorly. The axis from *Lepidoglyphus destructor* G6 (bottom left) to *Tyrolichus casei* T62 (top right) in the upper right hand panel of Fig. 18 matches the same shift from narrow slender snake teeth in species that need to maintain a grip on their active prey to short stout teeth associated with species that undergo high or repeated loads (Segall et al. 2023). *Suidasia pontifica* S5 is unusual at top left in the upper right hand panel of Fig 18 in having blunt teeth.

Similar results are found when examining the relationship of inter-tooth spacing with gullet nadir angle categorised by the mastication surface region being distal, intercalated or proximal (Fig. 18 Lower). Inter-tooth spacing estimates the gullet width in that region. Most taxa have modest size gullets overall of $\approx 100^{\circ }$. The lower left hand sub-panel shows the shallow distal gullet nadir angles and a general move to more pocketed gullets thereafter posteriorly. The overall pattern from *Lepidoglyphus destructor* G6 (bottom left) to *Tyrolichus casei* T62 (top right) and the unusual position of *Suidasia pontifica* S5 in the lower right hand panel of Fig. 18 remains. *Glycyphagus domesticus* G5 now sits near *Lepidoglyphus destructor* G6 at the lower right sub-panel bottom left. These general patterns are congruent with the rising and falling $R_{t}$ results.

### Do angles flatten as mastication surfaces lengthen?

Reduced dental topography (with increased strengthening) is associated with specialisation of molar form in mustelids (Selig 2023) in order to process hard material like bivalves and bones. Strengthening for durophagy (i.e., being able to sustain large occlusive loads has already been shown in some astigmatans by Bowman (2021, 2024b). In monkeys, hard fruit and seed eating species are called sclerocarpic harvesters. However, care is needed when assigning hard food adaptations Marcé-Nogué et al. (2020). Table 2 gives data on some known ‘crushing action’ durophages (see https://en.wikipedia.org/wiki/Durophagy) to favourably compare the bird nest astigmatan results to. However, what happens as moveable digits lengthen in bird nest astigmatans?

Across species, linear regression $R^2$ values in the range $0.006-0.0615$ show that average nadir angles or zenith angles whether for the first (distal) or last (proximal) or intercalated asperities are not related to moveable digit length (*L*2*M*, *plots not shown*). Similarly within species, with the exception of the intercalated tooth zenith angle for *Suidasia pontifica* S5 ($R^2=0.8259$), generally the distal, intercalated and proximal teeth zenith angles do not vary majorly in their own right with tooth row length ($x_{i_{e}}$), $R^2$ values vary from $10^{-5}$ (*Acarus farris* A17 distal tooth) to 0.3764 (*Tyrophagus palmarum* T17 proximal tooth flattening on elongation) over the taxa (*plots not shown*). Within species, distal, intercalated and proximal gullet nadir angles also do not vary majorly in their own right with tooth row length ($x_{i_{e}}$), $R^2$ values vary from $10^{-6}$ (*Chortoglyphus arcuatus* CH1 intercalated gullet) to 0.4509 (*Tyrophagus palmarum* T17 proximal gullet flattening on elongation) over the taxa (*plots not shown*). The surface of digits neither apparently ‘crinkles up’ on shortening nor smooths/flattens out on lengthening of the mastication surface within species (rather, asperity angles appear conserved).

**Fig. 19:**
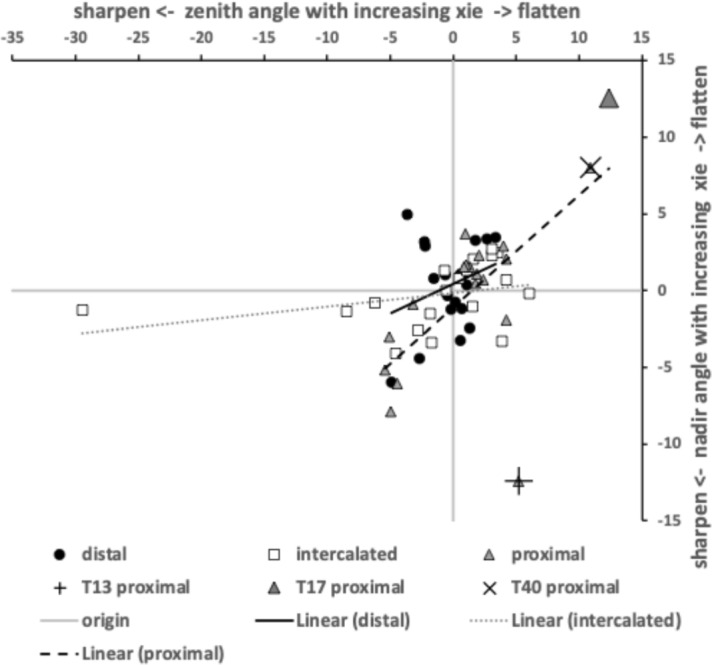
Plot for bird nest astigmatan moveable digits of within-taxa regression coefficients for each taxa and tooth row region of tooth zenith angle (*x*-axis) or within taxa regression coefficients of gullet nadir angle (*y*-axis) with increasing tooth row ($x_{i_{e}}$). Black dots = proximal asperities. White squares = intercalated asperities. Grey triangles = proximal asperities. Outliers from respective regressions highlighted as in legend. The top left black proximal dot is *Tyrophagus palmarum* T32. The proximal and distal regression lines have slopes through zero of 0.665 and 0.357 respectively so angles do not scale 1 : 1 with tooth row length changes overall

However, if the regression coefficient (i.e. the slope) of tooth zenith angle or gullet nadir angle with increasing tooth row length ($x_{_{e}}$) are plotted for each taxon and tooth type one obtains Fig. 19. This shows that the intercalated asperities do not behave congruently i.e., if tooth zenith angles sharpen on tooth row extension there is little if any change in intercalated gullet nadir angles for bird nest astigmatans in general. Changes are not concerted (even for the outlier *Suidasia pontifica* S5). However, for both distal and proximal asperities there is some evidence that for an evolutionary increase in tooth row size, if tooth zenith angles flatten, the corresponding gullet nadir angles also flatten within a region. That is, the ‘modules’ are internally coherent but independent of each other. Similarly, for both distal and proximal asperities there is some evidence that for an evolutionary increase in tooth row size, if tooth zenith angles sharpen, the corresponding gullet nadir angles sharpen i.e., they behave congruently. For bird nest astigmatans in general within-module changes are concerted. This is even over such noteworthy taxa as *Tyrophagus longior* T40 and *Tyrophagus palmarum* T17 where (in both), any tooth row extension causes a noticeable general flattening of the digit’s proximal asperities (cf. top right in Fig. 19). Again *Tyrophagus putrescentiae* T13 is confirmed as unusual, as with any extension of the tooth row, whilst the proximal tooth’s zenith angle gently flattens, the proximal gullet nadir angle markedly steepens (i.e., it becomes more ‘pocketed’ here near the ascending ramus). Larger and larger *Tyrophagus putrescentiae* T13 chelae therefore could catch onto, grab and strip larger and larger hyphal stalks. Their feeding tactic is coherently scalable.

**Fig. 20:**
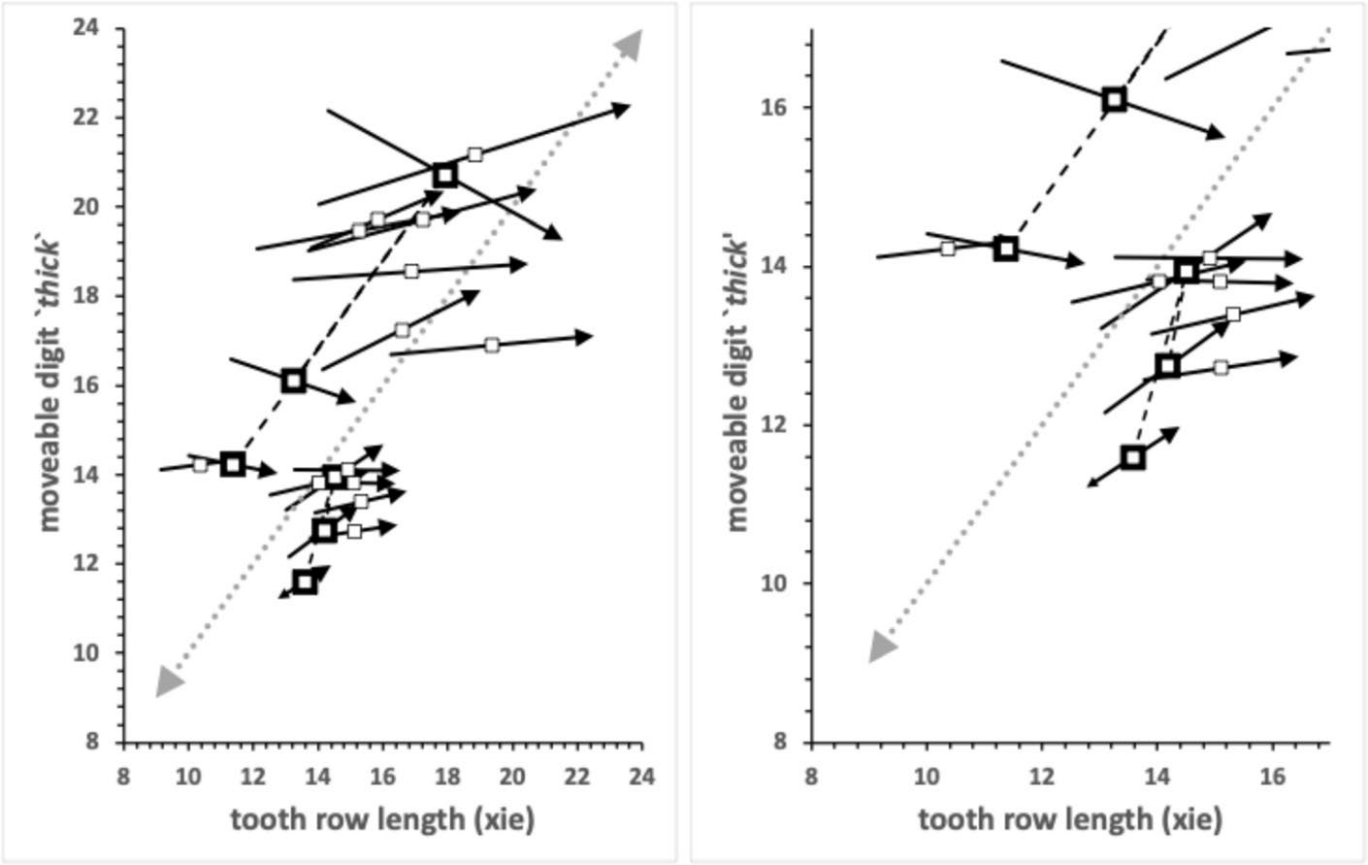
Plot of moveable digit *thick* versus tooth row length ($x_{i_{e}}$) for bird nest astigmatans. Small open circles mean for each taxon. Black squares joined by dashed lines highlighted species (see text). Arrows are within species regression slopes of *thick* by $x_{i_{e}}$ length scaled by adductive output force *F*2*AV* (from Bowman 2021). Grey double headed dotted arrow is line of equivalence (i.e., slope of one through zero). On left: all data. On right: zoomed-in lower area

Tooth row lengthening (indeed an increased output lever *L*2*M*) by size change or trophic adaptation may have other consequences like a fattening of the moveable digit or even a narrowing to a gracile form. If teeth thicken similarly as $x_{i_{e}}$ grows then (given the same output adductive force *F*2) the teeth may percussively ‘thump’ foodstuff to an extent rather than cleanly ‘chop’ it on chelal occlusion just like stone axes do compared to metal axes on tree felling (Mathieu and Meyer 1997). Figure 20 shows that between taxa, the tooth row length and moveable digit thickness generally scale together and the thickening of the digit follows as expected the increased output adductive force (in order to prevent chelal breakages). However, within taxa (despite low $R^2$ values) three groups are clear. Figure 20 shows from bottom left to top right:

- one group (upper black squares in this rising order) *Dermatophagoides pteronyssinus* D3, *Thyreophagus entomophagus* TH3, *Lepidoglyphus destructor* G6 which have strongly negative slopes in the left sub-panel;
- a second group (lower black squares in this rising order) *Acarus farris* A17, *Tyrophagus palmarum* T17, *Acarus gracilis* A4 which have strong positive slopes in the right sub-panel;
- and, the remainder with little if any slope (beware the illusionary visual exaggeration of large *F*2 values on judging slopes).

What does this mean? Firstly within each of the first two groups, with respect to tooth row length and digit thickness the taxa are generally scaled versions of each other (dashed lines). That is, they are commonly adapted. Secondly, a negative slope means that as the tooth row lengthens for a species, the moveable digit disproportionately thins becoming more slender like *Carpoglyphus lactis* does distally or like fast snapping veigaids i.e., they become more cutting style adapted. Thin flexible vertebrate mandibles are associated with ‘lunge feeding’ (Motani et al. 2015). Thirdly, a positive slope means that as the tooth row lengthens for a species, the moveable digit disproportionately thickens becoming more strengthened and robust (to better resist crushing/gripping loads). Finally for most bird nest astigmatans, tooth row lengthening drives no change in digit thickness suggesting that they are not designed extra-specially for snapping, crushing or gripping but rather perhaps to better facilitate protractive stabbing/slicing or retractive general tearing/cutting and squashing of foodstuffs.

### Tribological characterisations

**Fig. 21:**
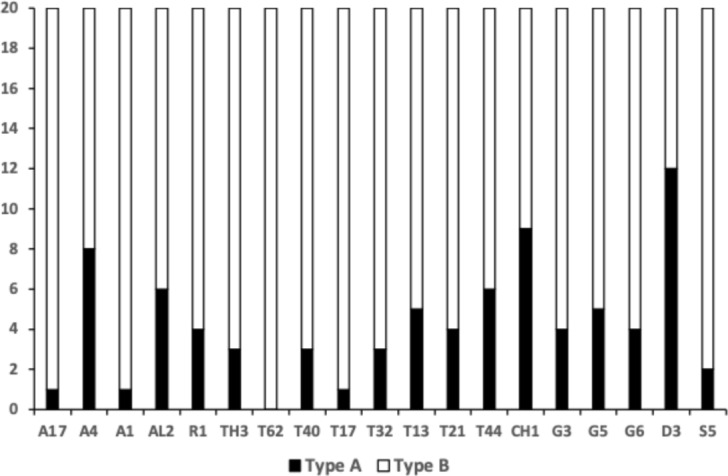
Number of individuals (out of 20) for each bird nest astigmatan taxon scored for moveable digit gross design type based upon maximum observed asperities. ‘Type A’-like = distal tearing/chewing, ‘Type B’-like = proximal nibbling (see Bowman 2024a). Overall proportion (A:B) = 0.21:0.79

Using the approaches of Fig. 24 in Bowman (2024a) but this time on the maximum observed asperities, one can grossly characterise individual bird nest astigmatans‘ whole moveable digit surfaces into a Type A-like (distal tearing/chewing) and Type B-like (proximal nibbling) designs. This is ignoring the subtleties of the regional asperities (in Fig. 6). Apart from in *Tyrolichus casei* T62, different individuals of those 20 assayed for each taxon showed such different ‘gross types’ in a rough 80:20 rule (B:A, Fig. 21). Of course this gross within taxon characterisation may be subject in part to drawing and measurement errors of digit orientation on microscope slides. However, from the point of view of any overall ‘hooking’ onto foodstuffs this apparent variation would allow within species trophic differentiation (i.e., "I do not exactly chew my food like my brother does at the same table"). This can be useful for a population (see Begon and Mortimer 1981).

Over all the taxa, bird nest astigmatans generally have a gross moveable digit design of a distally positioned maximum height tooth followed posteriorly by a proximally positioned maximum depth gullet (i.e., grossly like a nibbling Type B design). *Dermatophagoides pteronyssinus* D3, *Chortoglyphus arcuatus* CH1 and *Acarus gracilis* A4 notably diverge from this (in an opposite sense), having a tearing/chewing distally positioned maximum depth gullet followed by a proximally positioned maximum height tooth (effectively reinforcing not contradicting the default distal ‘hook’ architecture). Recall from Fig. 3 and Table 20 in Bowman (2024a) that, on balance, Type A designs are optimised for ‘catching upon’ foodstuffs on cheliceral protrusion and Type B designs are optimal on cheliceral retraction. It is likely therefore that individuals of *Dermatophagoides pteronyssinus* D3, *Chortoglyphus arcuatus* CH1 and *Acarus gracilis* A4 have the ability to grossly use their chelicerae overall in a distinctly different (spearing, knifing or stabbing) manner than most other bird nest astigmatans.

**Fig. 22:**
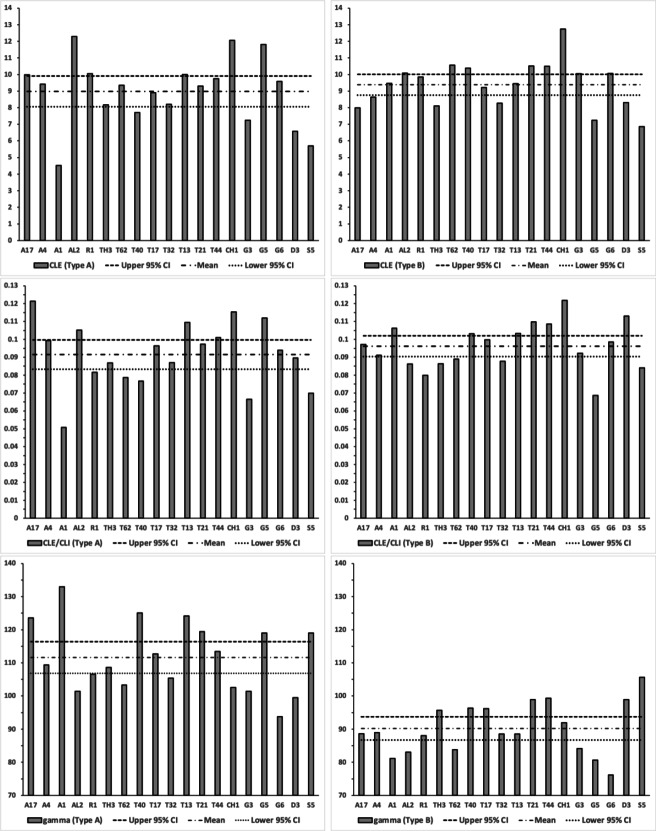
Claw length equivalents (*CLE*) and hook angles ($\gamma$) derived from observed asperities for bird nest astigmatans. On left for gross Type A moveable digit. On right for gross Type B moveable digit. First row: CLE values. Middle row: CLE values scaled by cheliceral length index (*CLI* from Bowman 2021). Bottom row: $\gamma$ hook angle. In each subfigure, upper line dashed line = upper unweighted 95% confidence interval, middle dashed and dotted line = overall mean, lower dotted line = lower 95% unweighted confidence interval, for comparison across taxa

Long thin claws in bats are associated with fishing (Wimsatt 1970). Examining the claw length equivalents (*CLE*) and hook angles ($\gamma$) for each gross Type following Bowman (2024a), shows various gryporial differentiations in bird nest astigmatans (Fig. 22).

- Irrespective of digit gross type, overall claw length equivalents are similar (Type A mean = $8.98\mu$m, Type B mean = $9.39\mu$m), indicating a common origin.
- Most bird nest astigmatan species for both Type A and B show little differentiation from an average *CLE* value for that Type.
- For the rarer Type A digits, *Acarus immobilis* A1, *Glycometrus hugheseae* G3 and *Suidasia pontifica* S5 have low *CLE* values which persist even after scaling for cheliceral size. This indicates trophic specialisation to grip (tear and chew) smaller objects distally. The very low Type A design $\frac{CLE}{CLI}$ value for *Acarus immobilis* A1 (at 0.05) is close to that for body scaled actual claw diameters calculated for terrestrial and aquatic oribatids (of 0.04 and 0.05 respectively from Pfingstl et al. 2020). Such oribatids may not need to ‘catch onto’ material to resist buffeting like intertidal species do? Perhaps they effectively stab and dig into small material with their claws? In that case, perhaps distally the mastication surface in *Acarus immobilis* A1 is used for scalporial ‘scratch-digging’ into foodstuff rather than a tearing/chewing style of ‘attachment’ with food? The low *CLE* value for *Dermatophagoides pteronyssinus* D3 is eliminated after rescaling showing it to be an overall mite magnitude effect.
- For the rarer Type A digits, high *CLE* values are shown by *Aleuroglyphus ovatus* AL2, *Chortoglyphus arcuatus* CH1 and *Glycyphagus domesticus* G5 which persist on rescaling. This indicates trophic specialisation to grip (tear and chew) larger objects. After rescaling, *Acarus farris* A17 and *Tyrophagus putrescentiae* T13 exhibit disproportionately high *CLE* values suggesting a degree of proximal relative differentiation compared to the basal astigmatan form.
- For the common Type B design digits, *Glycyphagus domesticus* G5 has noticeably low *CLE* values that persist after cheliceral length rescaling suggesting a distinct micro-form of ‘hooking’ and being effectively ‘attached’ to foodstuff. *Suidasia pontifica* S5 shows possibly low persistent values too (but this may be related to its small body size).
- For the common proximal nibbling Type B form, only *Chortoglyphus arcuatus* CH1 shows noticeably large claw length equivalent values compared to the other bird nest astigmatans, which in persisting after scaling suggest a different distinct trophic specialisation for ‘hooking’ larger objects. *Dermatophagoides pteronyssinus* D3 may exhibit some proximal relative disproportionality compared to the basal bird nest astigmatan form.
- Claw length equivalent values (across gross Types within taxa) are only mildly positively correlated between themselves (*plot not shown*, $R^2=0.1155$).
- $\gamma$ values differ between digit gross Types (mean for Type A = $112^{\circ }$, mean for Type B = $90^{\circ }$ as expected) and over many of the studied taxa.
- For the rarer distal tearing/chewing Type A digit design, lower more acute $\gamma$ angles were shown for *Aleuroglyphus ovatus* AL2, *Chortoglyphus arcuatus* CH1, *Glycometrus hugheseae* G3, *Lepidoglyphus destructor* G6, *Dermatophagoides pteronyssinus* D3 and perhaps *Tyrolichus casei* T62 indicating a more ‘pinched face’ style of trophic specialisation.
- For the rarer distal tearing/chewing Type A design, *Acarus farris* A17, *Acarus immobilis* A1 (particularly so), *Tyrophagus longior* T40 and *Tyrophagus putrescentiae* T13 show elevated more obtuse $\gamma$ angles indicating a more ‘open faced’ design for the moveable digit.
- CLE and $\gamma$ values are poorly correlated over gross Type A designs (*plot not shown*, $R^2=0.086$) although larger distal ‘hook’ designs have more acute angles. This latter hint might suggest that a larger overall surface fluctuation (of a maximum peak height following a maximum gullet depth) needs to be further apart i.e., such requires more evolutionary space distally along the digit in order to occur. However, this is not supported by a regression against mastication surface length ($x_{i_{e}}$, $R^2=0.0862$, *plot not shown*).
- For the common Type B digit design, lower more acute $\gamma$ angles were shown for *Acarus immobilis* A1, *Aleuroglyphus ovatus* AL2, *Tyrolichus casei* T62, *Glycometrus hugheseae* G3, *Glycyphagus domesticus* G5, and *Lepidoglyphus destructor* G6 indicating a more ‘pinched face’ style of trophic specialisation in proximal nibbling.
- For the common proximal nibbling Type B design, *Tyrophagus similis* T21, *Tyrophagus similis* T44, *Dermatophagoides pteronyssinus* D3 and particularly so *Suidasia pontifica* S5 show elevated more obtuse $\gamma$ angles indicating a more ‘open faced’ design for the moveable digit.
- CLE and $\gamma$ values are poorly correlated over gross Type B designs (*plot not shown*, $R^2=0.086$) with larger ‘hook’ designs having more acute angles. This again might suggest that a larger overall surface fluctuation (now of a maximum peak height preceding a maximum gullet depth) needs to be further apart i.e., such requires more evolutionary space along the digit to occur. Indeed a regression of $\gamma$ for proximal nibbling design Type B versus the length of mastication surface $x_{i_{e}}$ shows a sizeable negative relationship ($R^2=0.4767$, *plot not shown*). This is consilient with an argument that mites were small (and undifferentiated) plesiomorphically (Sidorchuk 2018).
- $\gamma$ values between the two design Types are essentially independent (*plot not shown*, $R^2=0.0436$), with $\gamma$ values for the rarer distally tearing/chewing Type A design varying widely across taxa with little consequent change in corresponding Type B $\gamma$ value.
- Values for *Glycyphagus domesticus* G5 and *Tyrophagus putrescentiae* T13 of *CLE* and $\gamma$ are in general agreement with those in Bowman (2024a) estimated in a different way from whole profile traces (Table 5) suggesting that this design differentiation is real. Note that all claw length equivalents are shorter and the $\gamma$ hook angles are more acute than the bee-hive inhabiting plesiomorphic *Carpoglyphus lactis*. Although the *CLE* values are about half that of real claw diameters for instance in oribatids (Pfingstl et al. 2020), the gross Type A (distal tearing/chewing design) $\gamma$ values overlap overall well with the claw angles of intertidal oribatids giving an external validation. Indeed the gross Type B (proximal nibbling design) $\gamma$ values correspond to the more acute claw angles of some rocky substrate inhabiting species (Pfingstl et al. 2020) validating the holdfast abilities of the bird nest astigmatan proximal mastication surface onto food micro-asperities. Furthermore scaling *CLE* values by reach (*CLI*) for all the bird nest astigmatan taxa gives a $min-max$ range of values for $\frac{CLE}{CLI}$ of: Type A $= 0.05-0.12$, Type B $=0.07-0.12$, encompassing the averages of body size scaled actual claw diameters in intertidal oribatids calculated from Pfingstl et al. (2020) (where: mangrove inhabiting = 0.09, rocky substrate inhabiting = 0.08, and mixed substrate inhabiting = 0.08).

**Table 5:** Comparison of moveable digit surface ‘hooking’ characteristics with those of bee-hive astigmatans shows the repeatability between studies despite using different methodology for *Glycyphagus domesticus* G5 and *Tyrophagus putrescentiae* T13 (Bowman 2024a)

Species	Taxon	*CLE* ($\mu$m) Type A	$\gamma$ in $^{\circ }$ Type A	*CLE* ($\mu$m) Type B	$\gamma$ in $^{\circ }$ Type B	$\frac{CLE}{CLI}$ Type A	$\frac{CLE}{CLI}$ Type B	References
*Carpoglyphus lactis*	bee-hive	13.7	150	10.5	92	0.14	0.11	Bowman (2024a)
*Glycyphagus domesticus*	bee-hive	11.2	135	10.3	71	0.10	0.09	Bowman (2024a)
	*Glycyphagus domesticus* G5	11.8	119	7.2	81	0.11	0.07	*this study*
*Tyrophagus putrescentiae*	bee-hive	10.9	139	6.2	65	0.11	0.07	Bowman (2024a)
	*Tyrophagus putrescentiae* T13	10.0	124	9.5	89	0.11	0.10	*this study*

What does this all mean? Within and between population variation in dentition is already known in humans, and is best explained by a polygenic model of inheritance (Bailit 1975).There is a theory that marsupial and placental mammal teeth are repeated organs that occupy different positions in a continuous morphogenetic field (Butler 1939). In other words, they are independent modules along a reference axis. A new synthesis of the aetiology of such morphological fields varying human dentition is available (Townsend et al. 2009). Indeed in insects, ‘appendage patterning genes’ are involved in mandible form (Coulcher 2011,Gotoh et al. 2017). There also is evidence that primate jaws with complex occlusal patterns are less variable than those mandibles with simple occlusion in order to function equally well (Gingerich and Schoeninger 1979). Functional integration is under different selective pressures in vertebrate carnivores (Meiri et al. 2005). Developmental processes and life-style strategy are also important (Reuter et al. 2021,Benowitz et al. 2018). Indeed, size is an important determinant of apparent variability in dentition (Polly 1998). Mites should be no different.

The gross Type A (distal tearing/chewing) design as a ‘module’ (or *composite* tool) concentrates feeding action at relatively low velocity ratio (*VR*) values. Output adductive force *F*2 for similar input force on the adductive tendon (*F*1) is thus lower. ‘Hooked’ foodstuff thus is likely to be softer. The maximum depth gullet fits around material and fragments that are stripped by the following-on maximum height tooth are likely to be swallowed whole. The moveable digit tip is acting like a vertebrate incisor and the maximum height tooth like a canine or premolar. This feeding action resembles a browsing or gleaning dinosaur (e.g., *Diplodocus*, Bowman 2025b). This style should match consuming fleshy food like nematodes too. Gut contents are likely to be ‘chunky’ but indistinct.

The commoner gross Type B (proximal nibbling design) as a ‘module’ or *composite* tool focuses feeding action at relatively high *VR* and *F*2 values suitable for ‘hooking’ and masticating drier harder foodstuff. The maximum depth gullet (after the maximum height tooth) fitting around material that is now trapped within the chela bracketed by effectively a premolar and the ascending ramus ‘molar’ for ruminant style trituration. Gut contents accordingly are less likely to be ‘chunky’ but should be distinct.

Claw length equivalent (*CLE*) values may be useful estimates in determining actual pre-ingestion triturated morsel size (see Discussion in Bowman 2023a). More research on the physical characterisation of the gut contents of different free-living astigmatans is needed.

**Fig. 23:**
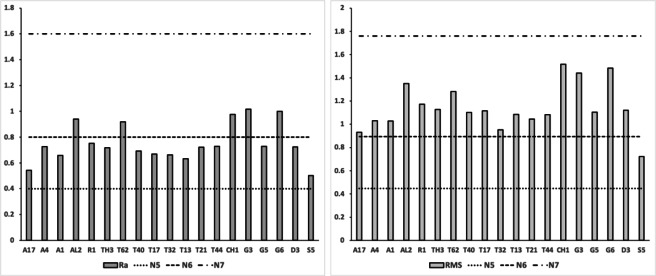
Tribological characteristics of mastication surface in bird nest astigmatans. On left $R_{a}$. On right *RMS*. Wild-collected *Carpoglyphus lactis* and laboratory sample Ca4 both have mean $R_{a}=0.39\mu$m and mean $RMS=0.68\mu$m (for comparison see Bowman 2024a)

Figure 23 shows that stochastically the mastication surface of the moveable digits of bird nest astigmatans map between International Roughness Grade Numbers N5 to N7. The two measures used are very highly correlated ($RMS \approx 1.5*R_{a}, R^2=0.9967$). This range of characteristics are the type of surfaces that have been ground (or do the grinding). Comparability with previous studies of *Glycyphagus domesticus* G5 and *Tyrophagus putrescentiae* T13 is high (despite the different estimation methods, Table 6). On balance *Aleuroglyphus ovatus* AL2, *Tyrolichus casei* T62, *Chortoglyphus arcuatus* CH1, *Glycometrus hugheseae* G3 and *Lepidoglyphus destructor* G6 have coarser (more ‘rasping’) surface properties than the remaining taxa. *Acarus farris* A17 and *Suidasia pontifica* S5 have the smoothest digits suggesting a fine grinding (or filing) action. Mite size has an impact here (e.g., $R^{2}=0.9817$ for a regression through zero of *RMS* versus reach, *plot not shown*).

**Table 6:** Comparison of moveable digit surface stochastic characteristics with those of bee-hive astigmatans (Bowman 2024a) shows apparent repeatability between studies despite using different methodology for *Glycyphagus domesticus* G5 and *Tyrophagus putrescentiae* T13 (see text)

Species	Taxon	*RMS*	$R_{a}$	*tel*	References
		($\mu$m)	($\mu$m)	($\mu$m)	
*Carpoglyphus lactis*	bee-hive	0.68	0.39	6.4	Bowman (2024a)
	Ca4	0.68	0.39	6.5	Bowman (2024a)
*Glycyphagus domesticus*	bee-hive	1.03	0.74	7.9	Bowman (2024a)
	*Glycyphagus domesticus* G5	1.02	0.69	7.6	Bowman (2024a)
	*Glycyphagus domesticus* G5	1.10	0.73	8.8	*this study*
*Tyrophagus putrescentiae*	bee-hive	0.85	0.67	6.4	Bowman (2024a)
	*Tyrophagus putrescentiae* T13	0.94	0.59	7.8	Bowman (2024a)
	Museum	0.73	0.52	5.5	Bowman (2024a)
	*Tyrophagus putrescentiae* T13	1.08	0.63	7.5	*this study*

**Fig. 24:**
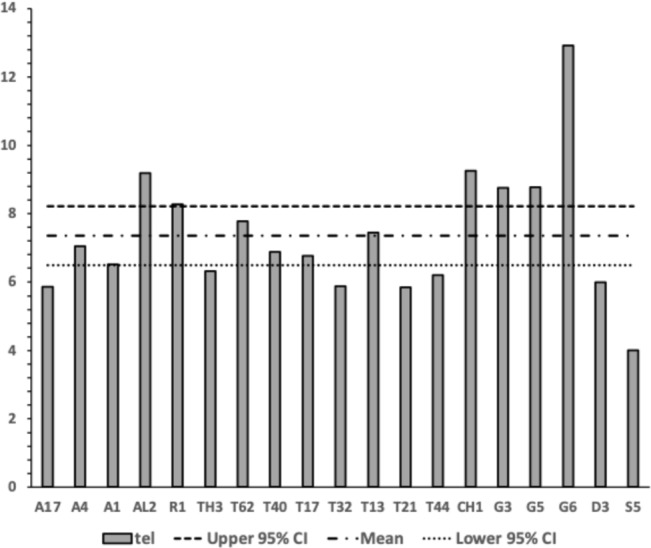
Developmental point process stretch measure (*tel* in $\mu$m, Bowman 2024a) for moveable digit mastication surface in bird nest astigmatans with mean between taxa and unweighted two-sided $95\%$ confidence interval

**Fig. 25:**
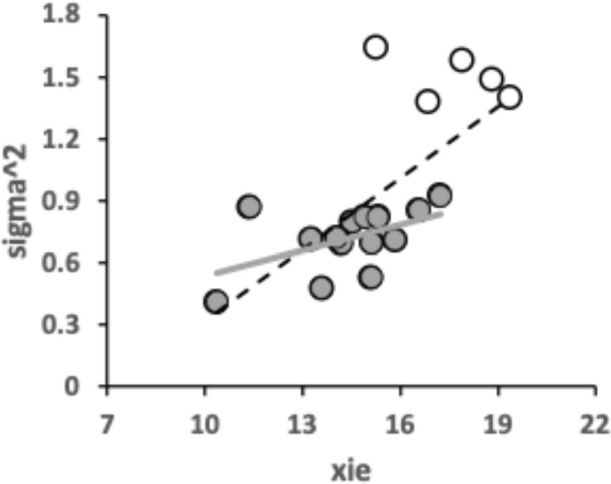
Mastication surface $\sigma ^{2}$ values versus tooth row length ($x_{i_{e}}$ in $\mu$m) for bird nest astigmatans (note overall dashed regression line $r=0.693$). Boundary for the $\frac{\sigma }{R_{a}}$ test of Seewig (2013) varies per taxon but is $\approx 1.2$ on the *y*-axis. Open dots = *Aleuroglyphus ovatus* AL2, *Tyrolichus casei* T62, *Chortoglyphus arcuatus* CH1, *Glycometrus hugheseae* G3 and *Lepidoglyphus destructor* G6 (showing differentiated surfaces i.e., the null hypothesis is rejected). Grey dots = remaining taxa whose moveable digits mimic a Gaussian fluctuating surface (grey solid regression line, $r=0.510$)

Figure 24 displays the developmental stretch measure (*tel*, see Bowman 2024a) for the mastication surface of the bird nest astigmatans studied. Table 6 seemingly shows acceptable repeatability for this point process measure between studies using the different methodologies, at least for *Glycyphagus domesticus* G5 and *Tyrophagus putrescentiae* T13. However in this study the moveable digit surface of many taxa would appear to be less stretched than the very smooth, micro-dentate *Carpoglyphus lactis* studied by Bowman (2024a) which cannot be true. The *tel* measure depends upon the number of elements in its summation and without normalisation cannot be used across studies (only within them). The mastication surface of *Aleuroglyphus ovatus* AL2, *Chortoglyphus arcuatus* CH1, *Glycometrus hugheseae* G3, and *Glycyphagus domesticus* G5 are more deformed developmentally than most other bird nest astigmatans (*Suidasia pontifica* S5 markedly less). However, only *Aleuroglyphus ovatus* AL2, *Tyrolichus casei* T62, *Chortoglyphus arcuatus* CH1, *Glycometrus hugheseae* G3 and *Lepidoglyphus destructor* G6 of the taxa studied fail the Seewig (2013) test for a Gaussian surface (*results not shown*). The dentition of these glycyphagids, the chortoglyphid and these two larger acarids are designed specially, not representing simple independent fluctuations along the adductive output lever arm *L*2*M* (like that for *Carpoglyphus lactis* from beehives or Ca4 in Bowman 2024a). These taxa are highlighted as open dots in Fig. 25. Of the remaining Gaussian surface group (highlighted as grey dots in Fig. 25), the top right pair are *Glycyphagus domesticus* G5 and *Rhizoglyphus robini* R1, the top left outlier is *Dermatophagoides pteronyssinus* D3 and the bottom left extremum is *Suidasia pontifica* S5. Size matters - bird nest astigmatan mites with larger tooth rows have more fluctuating or more differentiated mastication surfaces. That the mastication surface of *Glycyphagus domesticus* G5 is (comparatively deformed but still) Gaussian in nature agrees with that of *Glycyphagus domesticus* from beehives and *Glycyphagus domesticus* G5 previously in Bowman (2024a). That the mastication surface of *Tyrophagus putrescentiae* T13 is (comparatively less deformed but still) Gaussian in nature agrees with that of *Tyrophagus putrescentiae* from beehives, *Tyrophagus putrescentiae* T13 previously and Museum specimens in Bowman (2024a).

## Discussion

Aspects of this discussion will draw heavily upon publicly available detailed arguments by Peter Saveliev about what shape of sword is best for cutting (originally the Appendix for volume 4 in Saviliev 2020), which is formally acknowledged as a great source of insight. For more details on the physics of hand-held impact weapons, the 2022 treatise by George Turner and web pages starting at https://www.thearma.org/spotlight/GTA/motions_and_impacts.htm are also strongly recommended. Their detailed arguments, insights and conclusions in the above sources are used in the context of mite chelicerae under the aegis of ’Fair use’ (see https://guides.osu.edu/copyright/copyright-exceptions) for this review.

Size, shape and shape-within a size class is expected to be correlated with diet in astigmatans as in vertebrate marsupials Warburton et al. (2024). After the previous biomechanical analysis (Bowman (2021), and the dissective use of the whole dorsal surface of the chelal moveable digit (Bowman (2025b), four conundra (labeled (i)-(iv) below) around free-living bird nest astigmatan coexistence remained:

(i) Is *Tyrophagus putrescentiae* special? Indeed, what is it about the different whole profile forms of *Acarus gracilis* A4 and *Tyrophagus putrescentiae* T13 that facilitates them being able to live together in bird nests?
(ii) How can one functionally differentiate *Aleuroglyphus ovatus* AL2, *Chortoglyphus arcuatus* CH1, *Glycometrus hugheseae* G3 and *Glycyphagus domesticus* G5 in a practical way from each other?
(iii) How can *Acarus immobilis* A1, the ‘basal forms’ *Tyrophagus palmarum* T17 and *Tyrophagus similis* T21 all live together in bird nests despite being in the same overall trophic design class? Could nematophagy be the distinction?
(iv) How can the ‘basal forms’ *Tyrophagus palmarum* T32 and *Tyrophagus similis* T44 live together sharing the same overall trophic design class?

These conundra are considered below.

### Conundrum (i)—Is *Tyrophagus putrescentiae* special? and, How could digit tips matter?

This first question can be nicely answered ’yes" by examining where *Tyrophagus putrescentiae* T13 sits in Fig 6. The zenith angle for the asperity before the end of the mastication surface at $x_{i_{e}}$ versus the subsequent gullet nadir angle before the rise of the ascending ramus is unusual. Could this seemingly unique adaptation be a morphological reason for this species’ high trophic adaptation reported by Michalczyk-Wetula et al. (2021)? Indeed, one could consider *Tyrophagus putrescentiae* as ‘special’ as it appears ubiquitously: "...You can wipe it off a lab bench, find it in a field, get it in nests etc etc - all over the world..." (Owen Seeman *pers.comm.*).

Note that also, distally *Acarus farris* A17 has a fairly blunt first tooth and very shallow first gullet anterior of it commensurate with its almost plesiomorphic form. *Dermatophagoides pteronyssinus* D3, *Chortoglyphus arcuatus* CH1, *Glycyphagus domesticus* G5, and *Lepidoglyphus destructor* G6 are highly derived distally showing a sharp toothed saw-like Type B (see Bowman 2024a) moveable digit surface.

As expected (from its postulated fungal stripping function, Bowman 2024b), *Tyrophagus putrescentiae* T13 does indeed have a proximal blunt tooth and is strongly pocketed near the end of its mastication surface. Proximally a similarly blunt final tooth is shown by *Dermatophagoides pteronyssinus* D3 (and possibly *Aleuroglyphus ovatus* AL2) but it is now associated with a shallow final gullet. *Glycyphagus domesticus* G5 has the most derived form proximally with an almost right angle zenith final tooth and a subsequent very strong pocket before the end of the mastication surface. The latter species must use this pocket in a special way.

*Tyrophagus putrescentiae* is also not just a fungal feeder, being implicated in damaging seedlings (e.g. Murillo et al 2021) and eating nematodes (Walter et al 1986, Walia and Mathur 1995). As Owen Seeman (*pers.comm.* says "...It‘s an amazing ‘I’ll eat what I please, thank you very much’ mite...".

**Fig. 26:**
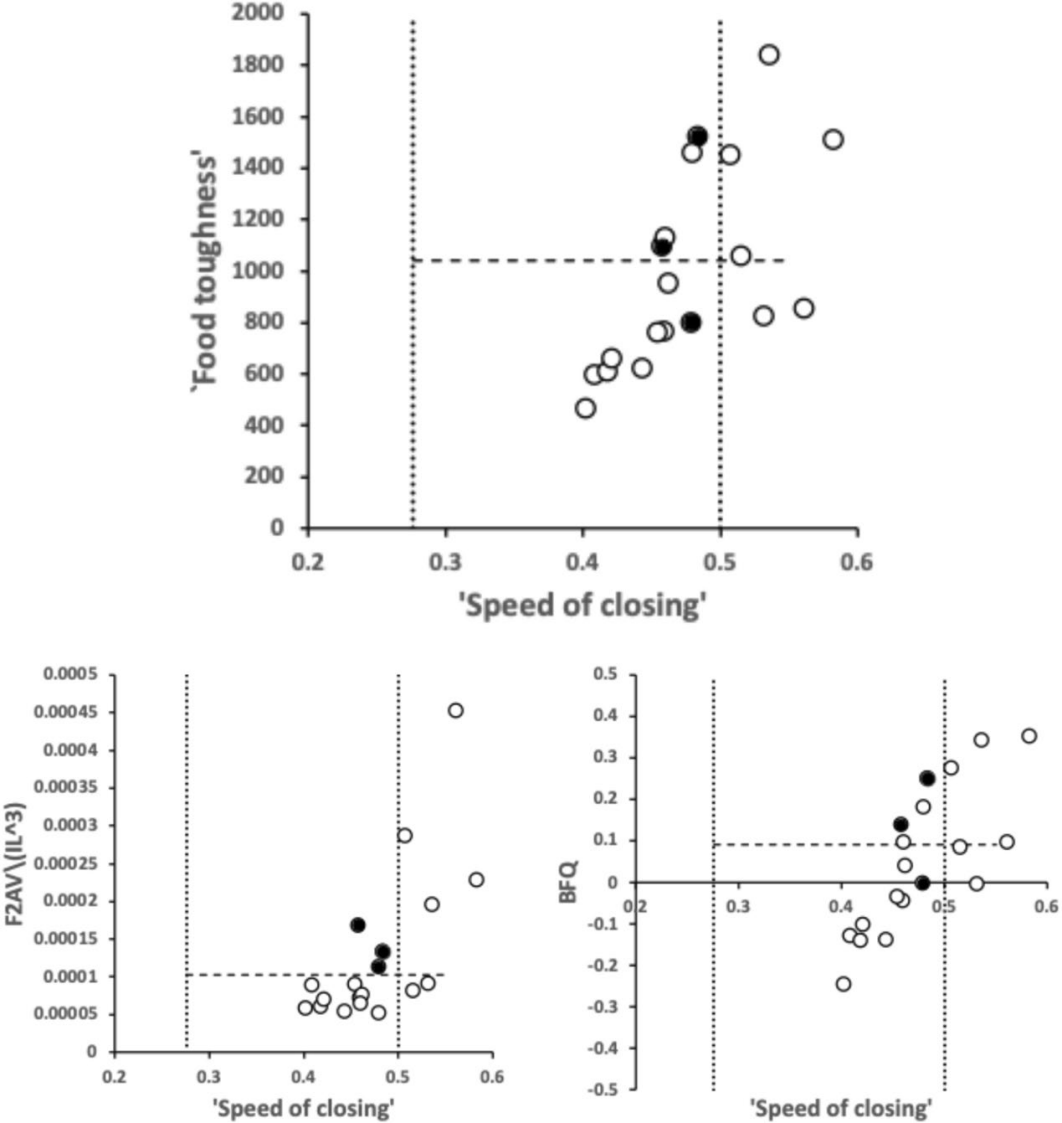
Upper: Plot of food toughness (*F*2*AV* ‘bite force’ extracted from Bowman 2021) versus moveable digit speed of closing (= tip velocity ratio from this study). Strengthened moveable digit tip taxa (from Bowman 2025b) highlighted in black. The latter form a particular saprophagous cheliceral design group amongst the largest typical acarid style astigmatans that feed upon nematodes (i.e., $VR\in (0.276...0.5)$), before progressive robustification yields a secondary decomposer design (on the right of the dotted line). These three taxa with potential scalporial (scratch-digging) adaptations cross the food toughness boundary (dashed horizontal line) from that typical of equivalent fragmentary and microphytophagous oribatids (rising from *Acarus gracilis* A4 at the bottom to *Glycyphagus domesticus* G5 in the middle) into a true typical microphytophagy design (*Lepidoglyphus destructor* G6 at top. Lower left: Scaling bite force by body mass ($IL^3$) shows tight specialised scalporial morphology group. Lower right: Bite force quotient (*BFQ*) estimated as residual from *loglog* regression of bite force (*F*2*AV*) by body mass (see Wroe et al. 2005) showing progressive over-investment in crushing to the right. *Acarus farris* A17 is at the most extreme bottom left. *Aleuroglyphus ovatus* AL2 and *Chortoglyphus arcuatus* CH1 to the top right

Figure 6 nicely shows that *Acarus gracilis* A4 does not have such special teeth and gullets. It can trophically co-exist with *Tyrophagus putrescentiae* - conundrum (i) is solved. Furthermore, the A4 former’s more than expected strengthened digit tips compared to most other bird nest taxa (Fig. 26) indicates a particular scalporial (scratch-digging) habit probably into nutritiously rich proteinaceous deposits (including dead bodies Bowman 2021). Chisel tooth digging (using incisors) into hard soil in order to consume hard subterranean parts of plants is known in fossorial rodents Rodrigues et al. (2023). If this was a majorly occurring habit in mites, mineral reinforcement of the chitin would be expected as in other arthropod mandibles (Cribb et al. 2008, Nalini and Polidori 2024). Such needs looking for in astigmatans.

By virtue of the fact that the astigmatan chela is often like a pair of crushing pliers (Bowman 2025b), this scratch-digging using moveable digit tips is not assumed to be a major function across the group in general except if the chela was opened wide and the chelicera protruded (or the gnathosoma moved forward by locomotion) much like the ‘excavation-bucket’ model for how uropodoid chelae with a Rollplatte might work (Bowman 2020, 2025b). *Tyrophagus putrescentiae* certainly is a geophage (Robaux et al. 1977) - see Bowman (2023a) for a comparison of mite cheliceral digging abilities. However, what about gryporial ‘hook-and-pull’ (on the backwards movement of digits) and what about piercing (needed for tensorial gripping, see Thomson and Motani 2021)?

Bowman (2024a) likens moveable digits to claw-like hooks when chelae are pulled (i.e., drawn) backwards, even if they work as piercing ‘stabbers’ when the chelicerae are protruded. Accordingly a digit tip angle can be estimated for astigmatan moveable digits (Bowman 2025b). This correlates well with the distal angle of the moveable digit as a triangle ($R^2=0.9793$, *plot not shown*). Indeed, Bowman (2023a) shows how the moveable digit vertical form (i.e., its depth) is in proportion to the opposable forces it experiences on crushing food. This is explicitly modelled in Bowman (2024b). So, in terms of just function (and not necessarily to avoid breakage or wear), accordingly

- Astigmatans with large velocity ratios (‘stubbier’ designs) should have blunter tip digits (i.e., larger tip angles) - they do for bird nest astigmatans ($R^{2}=0.9854$, *plot not shown*).
- Saprophages with large input moment lever arm forces (*F*1) should have blunter digit tips (i.e., larger tip angles) - they do for bird nest astigmatans ($R^{2}=0.9177$, *plot not shown*).
- Bluntness (i.e., larger tip angles) may be increased for bird nest astigmatan mites needing strong ‘saw base-plates’ (to resist bending and vibration). In fact it is not ($R^2<0.1285$ for *W*, *plot not shown*). Although *per force* this is true for distal digit angle.
- Facultative ‘stabbing’ should be facilitated by slender digits (i.e., small tip angles) - it is at least of fluids as in *Carpoglyphus lactis*.

This is consilient with the oribatid claw results discussed below.

Clearly the more tapered a claw or moveable digit tip is, the more it approximates a thin shaft (like the Japanese melee weapon, the Sai). Such a tool is suitable for poking or end-on piercing of substrates (or prey) much like Medieval pole-arms (e.g., staves, pikes, poleaxes etc., see https://en.wikipedia.org/wiki/Polearm and references therein). Even some specialist (i.e., compass) saws are very pointed in overall form ($\equiv$ small distal angles). For instance those illustrated in Disston & Sons (1918) give a range of values $\approx 4-6^{\circ }$. Keyhole saws are even finer.

Kerschbaumer and Pfignstl (2024) introduces the tip angle (indicating the ‘angle of attack’ of the tip) as a possible ecomorphological indicator in oribatid claws. A biomechanical trade-off was clear - while sharper claws confer superior interlocking capabilities with small surface asperities (a crucial factor for effective attachment), their structural delicacy makes them vulnerable to fracture and wear. So littoral oribatid mites generally tended to have blunter claws in line with the mechanical stress due to strong wave exposure. The bluntest claw tips were shown by rock dwelling species, apparently to reduce the risk of fracture and still provide the ability to grip (or grasp) onto the rough and coarse texture of the rocky shore habitat. Species mainly associated with mangrove habitats, on the other hand, had sharper claws, which might be a consequence of being less exposed to strong wave action and having to cope with smoother substrates like mangrove leaves (perhaps they pierce the plant tissues?). Claw bluntness in terrestrial animals also increases with body mass in order to cope with increased loads. Unusually claw sharpness in oribatids is inversely correlated with claw curvature (unlike in vertebrates).

Track, foundry and Blacksmith’s chisels approximate a pounding or cutting tool of equivalent angle $\approx 11-31^{\circ }$ (https://www.blackburntools.com/articles/rose-tools-catalog-archives/pdfs/woodings-verona-catalogue-16.pdf). These (like cold chisels and ‘picks’) are all much sharper than either the mite distal digit angle or the tip angle values reported by Bowman (2025b). The somewhat elevated tip angle values in *Glycometrus hugheseae* G3, *Glycyphagus domesticus* G5, *Lepidoglyphus destructor* G6 and *Rhizoglyphus robini* R1 compared to the acarids suggest a weaker piercing action in those species (given the same cheliceral protrusive force). Indeed these are blunter than the $\approx 36^{\circ }$ tip of a pattern makers’ saw (see page 151 in Disston & Sons 1918 or the $\approx 42^{\circ }$ tip of a rounded nosed saw like a flooring saw (see D19 page 141 in Disston & Sons 1918). They better match the rounder tip of some pruning saws (see p153 Disston & Sons 1918) and particularly the $\approx 56^{\circ }$ tip of a half back bench saw (see No 8 page 144 in Disston & Sons 1918). If they were not rasping tools but percussive hammers, they approximate some Blacksmith’s sledges (at $\approx 55^{\circ }$, see No 28 in https://www.blackburntools.com/articles/rose-tools-catalog-archives/pdfs/woodings-verona-catalogue-16.pdf).

Digit tip angles in bird nest astigmatans are $\approx 50^{\circ }$Bowman (2025b). Thomson and Motani (2021) gives angles of attack for vertebrate claws matching this value to Suspensorial sub-types (i.e., hanging claws in sloths). However, variation in this angle was not very informative between vertebrate claw sub-types - only at the extremes would Scalporial (scratch-digging), Cursorial, (running/hopping) or Tenasorial (grappling) claws be considered distinct. The angle of attack in vertebrate claws was found to positively correlate with their (ventral) curvature angle, To check this for mites, using the aspect ratio $ogcar=\frac{falling\ R_{t}}{x_{i_{e}}}$ as a surrogate of overall gullet curvature in mites, there was mild evidence ($r=0.314$, *plot not shown*) that the bigger the digit tip angle the sharper the relative overall gullet depth is i.e., the greater the moveable digit overall curvature. This shared phenomenon was interpreted mechanically by Thomson and Motani (2021) in their section 4.4. Thin (therefore sharp-tipped) dentaries are found in the earliest ceratopsids (Nabavizadeh 2023) and it is accepted by acarologists that the plesiomorphic form of a moveable digit is that of an unornamented bar (i.e., that it is descended from a tibiotarsal setal ambulacrum/claw), so one would expect digit sharpness (i.e., small tip angles) to be associated with least curvature. Again consilient with the (unusual) oribatid results. Indeed the same result is found in the bird nest astigmatans using the *ogcar* aspect ratio.

Furthermore, Turnbull et al. (2023) give tip angles for varanid lizards contrasting mean values of $46.9^{\circ }$ for an arboreal (‘climbing’) species with $20.6^{\circ }$ and $33.6^{\circ }$ for two burrowing species. This again points to a ’foodstuff’ burrowing function for most moveable digit tips in bird nest astigmatans. The latter authors also define a ‘profile rigidity ratio’ equivalent to $\approx \frac{ogcar}{tip\ angle}$ herein. This "... profile-view indication of claw rigidity..." suggests that the moveable digits of *Chortoglyphus arcuatus* CH1 and *Dermatophagoides pteronyssinus* D3 in particular are more suitable for a burrowing action into foodstuff than other taxa (*plot not shown*). As Turnbull et al. (2023) says "... This is because (a) structures with greater curvatures have to carry bending moments, shear forces and torsional moments, and as the curvature is decreased the torsional moment reduces until it reaches a zero-value (for flat structures where there is only shear and bending), and (b) a larger tip angle means there is more material at the tip in the direction of claw loading....".

One argument for large blunt flat claws (or chelal digits) in mites would be to not penetrate soft substrates (such as mangrove-like material when considering oribatid legs) but simply rely upon surface tension to hold the claw surface ‘stuck’ against a wet surface in micro-cavities. Surface tension is a measure of work per unit area or force per wetted length. Recall that mites, by virtue of their size, do not walk on a substrate but through (i.e., within) any non-compacted substrate. A liminal environment should provide many such semi-submerged crevices. The physics of gripping deformable objects in wet environments is complicated (see Nguyen et al. 2020). For sure, counterfactually, the potentially skimming chelal digit of *Carpoglyphus lactis* has an observed sharp tip angle to ensure it actually does penetrate material and not just rest on liquid meniscal surfaces for grip.

*For the ‘expert predatory weapon designer’*, why is this so?

The following justification assumes that homogeneous material is being attacked. Experience with weaponry (for instance https://www.thearma.org/spotlight/GTA/motions_and_impacts.htm and links therein) indicates that the best geometry for a straight thrusting blade is the design closest to a sharp needle i.e., “...the narrower the blade the better...”. Moreover, the point should be centred to the axis of thrust (and the weapon thus should be straight in form - the curvier the worse it will perform in a straight thrust). Indeed, if it was curvy then the thrust needs to be a matching curve-shaped trajectory for efficiency. Similarly removal of a curved blade would require a (reversed) circular action too. By virtue of chelal moveable digit tips rotating (at a distance) around the condyle this curved movement is ensured of course.

Suggested tip flexibility (Bowman 2024b) may allow small-scale fine angle optimisation of moveable digit entry into rough food material as well as a ‘spring-action’ bending to compensate for high impact loads on collisions. For a discussion on blade flexure and vibration see George Turner, Part 3 https://www.thearma.org/spotlight/GTA/motions_and_impacts3.htm. Note that an oblique cut not only tends to dissipate energy into side-to-side oscillations, but will also cause the apparent blade cross-section to be "...side-warded...". This means that the moveable digit is trying to cut on a slant, which builds up much higher foodstuff target resistance. This all puts higher deceleration forces on the blade, which will increase the flexure forces in both the edge-to-edge and side-to-side planes.

Now as experience with weapons shows, a circular thrust with a long sword-like tool is not easy - curvier stabbing weapons are used therefore for close-quarters fighting. For example, the curved shape of the chopping and slashing Nepalese Gurkha kukri creates a ‘wedge’ effect which effectively cuts deeper. So cheliceral reach will therefore come into play here in determining mite digit designs. However, there is only weak evidence for low reach engendering increased digit curvature and vice versa in bird nest astigmatans (*plot not shown of surrogate aspect ratio* $\frac{falling\ R_{t}}{x_{i_{e}}}$ *versus CLI*).

If the tool’s point is not centred then the thrusting power is exactly the same, but the point of such blades are geometrically weaker than those with a centred point. The reason is that a centred point maximises the angle of the point (tangentially to the target at $90^{\circ }$) focusing the force. That angle would be less in any other position so the blade would be weaker. As has been pointed out by others, Galileo knew as long ago as in 1598 that the impact of a cannon ball was maximal when the target surface was perpendicular to the projectile’s trajectory at impact. Further, the smaller the surface area that the inertia of a cut is concentrated upon, the greater the impact effect will be (J Clement, What Makes an Effective Sword Cut? https://www.thearma.org/essays/howacutworks.htm). One concludes therefore that oribatid claws may not stab but solely ‘hook’ (with mangrove inhabiting species perhaps ‘standing off’ further from their substrate than other species?).

Indeed regardless of the shape of the blade and the initial volume of food or prey material first hit, it is the *whole* volume of material that will oppose (i.e., resist) the digit blade. Following Francisco Urbano Garci who explained in 2008 (The best geometry for blades - https://www.thearma.org):

The *Resistance* of a target given a *t* thrusting distance for a triangle with a base *b*, a height *H* and a point placed at a distance *d* from one of the sides of the stabbing blade edge is

$$= t.cos(\frac{\varPi }{2}-tan^{-1}(\frac{(b-d)}{H}).\sqrt{H^{2}+(b-d){^2}} + t.cos(\frac{\varPi }{2}-tan^{-1}(\frac{d}{H})).\sqrt{H^{2}+d^{2})}$$

He simplifies this (over several steps, since *H* is always positive) to give

$$\Rightarrow Resistance = b.t$$

meaning that the only important thing when thrusting a weapon (like a mite’s spike-like moveable digit into potential food) is how wide (*b*) it is and how deep (*t*) one wishes to thrust it in. Thus the exact geometry of the (astigmatan moveable digit or oribatid claw) tip *in terms of resistance* does not matter. Indeed, experimental work with hefted prehistoric stone projectiles (Sitton et al 2020) shows that tip cross-sectional area (TCSA) and tip cross-sectional perimeter (TCSP), each of which depend upon *b*, both exhibit a strong, significant inverse relationship with target penetration depth.

However, if one accepts that the wider the tip the stronger and the more durable it is, one would like to know which design (within all those possible geometries with the same penetration power) offers a wider angle in the tip. Again, following Francisco Urbano Garci (The best geometry for blades - https://www.thearma.org), the angle for every possible triangle geometry in a blade is expressed by:

$$Angle = tan^{-1}\left( \frac{d}{H}\right) +tan^{-1}\left( \frac{(b-d)}{H}\right)$$

So, since one wants to maximise this, Garci solves the equation of its $derivative=0$ for *d* which (after simplification) gives him

$$d=\frac{b}{2}$$

This means that the distance of the point *d* must be just in the middle of the blade (i.e., $\frac{b}{2}$ to have the maximum angle, and thus, the maximum strength and durability). This appears to be approximately true for the terminal part of oribatid claws (e.g., see the figures in Kerschbaumer and Pfignstl 2024) but this is not unequivocally clear for the astigmatan moveable digits. For sure due to the strengthened shape of the moveable digit distally, the location of the distal tip angle is not bisecting the central part of the distal angle of the moveable digit (compare Fig. 4 with Fig. 5 in Bowman 2025b). However, it is the case for the edges of conventional blades in food processing machines (Bremer and Matthiesen 2020) where the ‘blade angle’ is symmetric to the blade’s centre. More research of astigmatans is needed.

### Conundrum (ii)- Do some species have a moveable digit mastication surface like a saw?

Figure 32 in Bowman (2025b) highlights the moveable digits of *Rhizoglyphus robini* R1, *Glycometrus hugheseae* G3, *Glycyphagus domesticus* G5 and *Lepidoglyphus destructor* G6 as being designed for cutting rather than the more typical astigmatan crushing action, in that the four species’ designs approximate class 3 lever systems. The latter form has a disadvantage for a herbivore (see Maiorino et al. 2018 for detailed references). Tooth set and (base-plate) thickness are discussed in Bowman (2024a) and as scanning electron microscopy is not used in this review the former is not elaborated upon for the bird nest habitat. Although baseplate thickness should act like increased claw depth in lizards to facilitate strength and attachment to rough surfaces (see Zani 2000).

Putting the results of Figs. 6 and 9 together suggests that distally the typical free-living astigmatan dentition design is a Type ‘A’ claw-like ‘hook’ (*Acarus farris* A17 being the least derived) followed proximally, by a blunter standard saw-like profile. Five clearly different derived designs are present (in alphabetic order of code): *Aleuroglyphus ovatus* AL2 versus [*Chortoglyphus arcuatus* CH1 $+$ *Lepidoglyphus destructor* G6] versus *Dermatophagoides pteronyssinus* D3 versus *Glycyphagus domesticus* G5 versus *Tyrophagus putrescentiae* T13.

Bowman (2025b) reports that *Aleuroglyphus ovatus* AL2 and *Chortoglyphus arcuatus* CH1 are very high kerf, average characteristic angle $\alpha <90^{\circ }$ species, while *Glycometrus hugheseae* G3 and *Glycyphagus domesticus* G5 are high kerf, average characteristic angle $\alpha>90^{\circ }$ species. Bowman (2025b) reports that *Acarus farris* A17 is a particularly low kerf, average characteristic angle $\alpha \approx 90^{\circ }$ species. This means that the high angle species have a saw like push/pull Class 1 lever cutting action mastication surface, while the lower angle taxa have a rotating gripping and crushing Class 3 lever action chela. These two latter points resolve conundrum (iv).

**Table 7:** Markov transition probabilities of tooth type (ATB$\rightarrow$FT or FT$\rightarrow$ATB) from distal tip to proximal end of moveable digit mastication surface in free-living astigmatans reported as common in bird nests with proposed interpretation as a tool type pattern. ATB = alternate top bevel tooth-like. FT = flat top tooth-like. Taxon ordered in genus and species within family. ‘Persistent’ indicates a tendency for ‘runs’ of a particular style teeth repeatedly one after another. ‘Toothed’ indicates a predisposition not to be FT blade-like. * = fails test for a Gaussian surface (*see text*)

Taxon						
	Tooth $\Rightarrow$	ATB		FT		Tool type
	followed by	same	‘switches’	same	‘switches’	pattern
*Acarus farris* A17		0.47	0.53	0.43	0.57	random
*Acarus gracilis* A4		0.59	0.41	0.50	0.50	random
*Acarus immobilis* A1		0.41	0.59	0.36	0.64	random
*Aleuroglyphus ovatus* AL2		0.42	0.58	0.71	0.29	bladed *
*Rhizoglyphus robini* R1		0.25	0.75	0.29	0.71	alternates
*Thyreophagus entomophagus* TH3		0.63	0.38	0.89	0.11	persistent
*Tyrolichus casei* T62		0.26	0.74	0.29	0.71	alternates *
*Tyrophagus longior* T40		0.39	0.61	0.69	0.31	bladed
*Tyrophagus palmarum* T17		0.19	0.81	0.60	0.40	bladed
*Tyrophagus palmarum* T32		0.52	0.48	0.56	0.44	random
*Tyrophagus putrescentiae* T13		0.71	0.29	0.78	0.22	persistent
*Tyrophagus similis* T21		0.15	0.85	0.92	0.08	bladed
*Tyrophagus similis* T44		0.41	0.59	0.70	0.30	bladed
*Chortoglyphus arcuatus* CH1		0.48	0.52	0.75	0.25	blade-like *
*Glycometrus hugheseae* G3		0.70	0.30	0.00	1.00	toothed *
*Glycyphagus domesticus* G5		0.83	0.17	0.25	0.75	saw-like
*Lepidoglyphus destructor* G6		0.86	0.14	0.40	0.60	toothed saw-like *
*Dermatophagoides pteronyssinus* D3		0.28	0.72	1.00	0.00	strong blade-like
*Suidasia pontifica* S5		0.25	0.75	0.82	0.18	bladed

Indeed one can go further. An analysis along the mastication surface of how each tooth type is (posteriorly) followed by another type is given in Table 7). Those mastication surfaces labeled as ‘random’ e.g., all three *Acarus* spp.), ergodically fluctuate like a wavy plesiomorphic bar-like digit along their length. Of the remainder, approximately half are blade-like. The mastication surface of *Rhizoglyphus robini* R1 alternates between a ‘tooth region’ versus a ‘blade region’. *Thyreophagus entomophagus* TH3 and *Tyrophagus putrescentiae* T13 both characterised as ‘persistent’ have coherent runs of teeth and runs of blades. One could think of such teeth here as ‘(pre-)molars’ and think of the blades as ‘incisors’ in function. The glycyphagids show saw-like moveable digit surfaces.

A tool with a geometry of horizontally aligned gullet nadirs and horizontally aligned tooth zeniths makes a ‘Simple saw’. Figure 15 lower, has minimum gullet depth changes for *Acarus gracilis* A4, *Glycometrus hugheseae* G3, *Glycyphagus domesticus* G5 and *Lepidoglyphus destructor* G6 anterior$\rightarrow$posteriorly. Figure 15 upper shows that ignoring the distal region, tooth height changes (anterior$\rightarrow$posteriorly) are smallest in *Acarus gracilis* A4, *Thyreophagus entomophagus* TH3, *Tyrophagus palmarum* T32, *Glycyphagus domesticus* G5 and *Suidasia pontifica* S5. This suggests that the moveable digits of *Acarus gracilis* A4 and *Glycyphagus domesticus* G5 (at least) might be considered as approximating a ‘Simple saw’.

The shape of gullets (although introduced above in the context of rake angle and back angle) are trivially matched to the shape of teeth by considering their back angle and rake. An aggressive rake angle and slightly larger gullets indicates a general purpose saw, since rake angle can be thought of as the angle at which the tooth tip enters the material. If a saw tip enters material at an angle it will be more efficient than if it slaps down flat (thus halberds are more damaging than axes in close combat). So in the context of this review, as expected, the more negative the overall rake angle the flatter the surface that hits the food is and the shallower the relative depth of the overall gullet *ogcar* measure is (*plot not shown*).

**Fig. 27:**
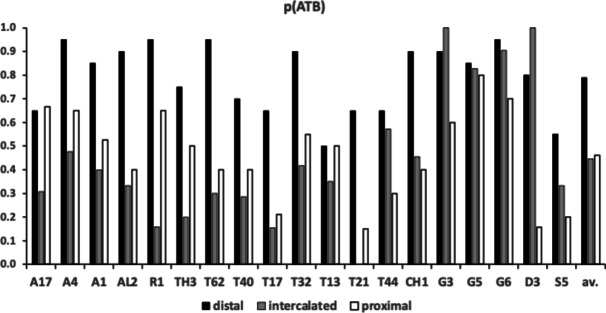
Probability tooth was scored as ‘likely to be ATB’ by region of the bird nest astigmatan moveable digit. av. = mean over taxa. Note probability of being flat-topped (‘FT’) and thus potentially ‘blade-like’ (if a long tooth) is $=1-p(ATB)$

Moreover, any high ATB teeth on a general purpose saw will yield ultra smooth cuts for cross-cutting use. Figure 27 shows that the distal teeth of bird nest astigmatan moveable digits in general have a $\approx `3:1$ on’ chance of being scored as ’likely to be ATB’. In the intercalated and proximal regions this is more like 50 : 50. Furthermore, *Glycometrus hugheseae* G3, *Glycyphagus domesticus* G5, *Lepidoglyphus destructor* G6 and *Dermatophagoides pteronyssinus* D3 rarely have (blade-like) flat-topped intercalated teeth unlike some of the acarids (e.g., *Rhizoglyphus robini* R1, *Thyreophagus entomophagus* TH3, *Tyrophagus palmarum* T17 and in particular *Tyrophagus similis* T21). So the former glycyphagid group’s moveable digits really are like cross-cutting saws, the latter group’s are more like ‘slicers’. The glycyphagids (again) and also *Acarus farris* A17, *Acarus gracilis* A4 and *Rhizoglyphus robini* R1 have a higher p(ATB) than on average proximally suitable for smooth cut sawing, with *Dermatophagoides pteronyssinus* D3, *Tyrophagus palmarum* T17 and *Tyrophagus similis* T21 being invariably flat-topped (blade-like) in this region near the condyle. Plotting average inter-peak (or inter-valley) spacing over the three moveable digit regions (from Fig. 12) scaled by tooth row length ($x_{i_{e}}$) shows *Glycyphagus domesticus* G5 and *Lepidoglyphus destructor* G6 have markedly lower values than the across taxa average (*plots not shown*). These taxa have particularly ‘grouped-together’ asperities like a ‘pukka’ saw.

Also, it should be pointed out that:

- The tips of high quality saw blade teeth often have hardened thick carbide tips to stay sharp and cut cleanly. These are brazed (i.e., fused) to the saw base-plate with sandwiches of special layers to provide extra flexibility and impact resistance. One would expect equivalent strengthening in mites. Follow-up work is needed to better understand the ultrastructure of acarine teeth.
- Examining if acarine predators with ATB teeth in general also produce ultra-smooth cuts under SEM would validate chitin being melamine-like in any follow-up work.

**Fig. 28:**
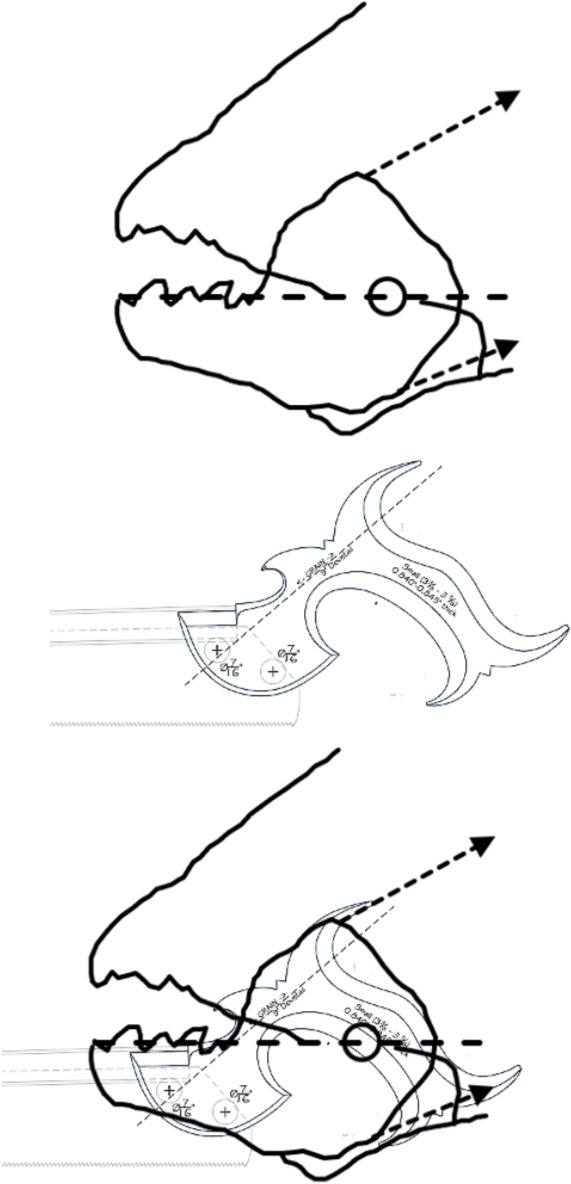
Slab-like basal rami are optimised for pushing. *Lepidoglyphus destructor* (G6) 20th individual (upper) showing overall design matches that of a 9 inch dovetail saw with open handle (middle, from https://www.blackburntools.com/articles/saw-handle-templates/index.html © 2011-2019, Isaac Smith with permission). Dashed line is reference axis (extended *l*2*M* through condyle for clarity). Lower, overlay showing: how tendon angle match horns on handle, mite cutting surface lines up with the saw upper reinforced plate, and handle attachment reinforcement (and leading profile) matches the moveable digit body under the horizontal ramus

Indeed considering the design posteriorly behind the end of the mastication surface shows that those species (e.g., *Lepidoglyphus destructor* G6) with a slab-like basal ramus (Bowman 2025b) match the design of a saw handle (Fig. 28). The condyle where any cheliceral protrusion force would act upon the moveable digit is where the human hand would grasp the equivalent saw. The horned ‘tangs’ on the handle are the leverage points for the edge of the human hand when rocking the direction of the blade during use. These consiliently are in the same place and point in the same direction as the adductive and abductive tendons (dotted lines) in mites.

Some such ‘back saws’ have the toothed surface uppermost (see Back Saw No 14, page 143 in Disston & Sons 1918) with their stiff strengthening lowermost exactly matching the astigmatan moveable digit. In practice, of course, any actual physical ‘tang’ for the abductive (opening tendon) would need to move (impossibly) outside of the cheliceral shaft on chelal opening, so in mites it is missing (just as in a Turkish saw, see page 1515 in Disston & Sons 1918).

Using Isaac Smith’s analysis and terminology (https://www.blackburntools.com/articles/hang-angles/backsaw-fbd.pdf, with his permission) of forces on a handsaw when sawing, leads one to the implications that:

- A plesiomorphic straight spike as a moveable digit could not act as a saw on chela protrusion or retraction (since it would be essentially smooth and "weightless" and "...glide over the..." material "...without cutting".
- As the handsaw’s ‘hang angle’ ($\varTheta$ *sensu* Isaac Smith) matches the design parameter $\alpha$ for a Class 1 lever (see Fig. 31 in Bowman 2025b), such moveable digits (in the common crushing feeding style astigmatans) are more likely to act rather like a saw when thrust forward by the chelicera advancing. Conversely the cutting feeding style Class 3 lever astigmatans (like *Glycometrus hugheseae* G3, *Glycyphagus domesticus G5*, *Lepidoglyphus destructor* G6 and *Rhizoglyphus robini* R1) more likely have a digit saw action on cheliceral retraction. The mythical ‘Pushmi-Pullyu’ creature from a children’s story (https://en.wikipedia.org/wiki/The_Story_of_Doctor_Dolittle) comes to mind here..
- Applying a chelal occlusive force will increase any sawing action on simultaneous cheliceral movement (as Isaac Smith’s $F_b$ is increased upwards).
- Forming an increasing ascending/basal ramus not only improves the leverage characteristics (see the synapsid jaw evolution argument around any *L*1*U* increase used in Bowman 2020 and the results in caviomorph rodent mandibles Álvarez et al. 2021) but will also facilitate a sawing action if asperities are present as the consequent hang angle alters.

However, there was no clear relationship of rake and back angle with the design parameter $\alpha$ (and thus the hang angle, *plots not shown*) in this review. Moreover, as in mites the mastication surface is on the top of the moveable digit (i.e., the opposite of most hand saws) increasing the weight of the digit by robustification against breakage (i.e., by thickening or sclerotisation or other structural strengthening, Bowman 2024b), or in proportion to gnathosomatisation (i.e., relative oral magnitude) or body size, effectively reduces Isaac Smith’s $F_{b}$ (upwards) and so the chelal occlusive force is more important in any saw action when the chelicera moves anterior$\rightarrow$posterior. Furthermore, increasing the hang angle for a moveable digit being pushed forward or backward decreases the upward force on the foodstuff so allowing the material to better resist being sliced by the asperities as they are dragged through it (i.e., Isaac Smith’s $F_{c}$ increases).

A note of caution is included here about subtle changes in ramus (especially coronoid) morphology. These are known to vary within and between human populations and throughout evolution. However, they are thought "...unlikely to be representative of differences in masticatory biomechanics and/or paramasticatory behaviours..." by Terhune et al. (2018).

### Conundrum (iii) - Which digit pattern might be good for feeding upon nematodes?

Birds suffer parasitic nematode infestations which depend upon the birds‘ diet and their detailed habitat (Leung and Koprivnikar 2016). Nematodes are also widespread in soils and their abundance and diversity are affected by bird activity (Pen-Mouratov and Dayan 2019). Ground birds’ nest astigmatans must be exposed to nematodes therefore as a potential food source. Even vertebrate herbivores like the hippopotamus eat carrion. Indeed *Tyrophagus* spp. are recorded as feeding upon nematodes (Bilgrami and Tahseen 1992, Walter et al 1986). Other acarids, like *Caloglyphus* spp. do too (Muraoka and Ishibashi 1976). Perhaps some astigmatans might therefore be better described as omnivores like pigs?

Feeding upon nematodes (like feeding on insects) is essentially eating ".... a hard covered package with soft insides..." Swartz et al. (2003). Predation requires two activities: prey restraint/immobilisation, versus prey dismemberment/consumption (Fowler et al 2009). The latter authors found that hypertrophied talons (claws) in birds have evolved primarily to restrain large struggling prey. Claw-like acarine jaws must function similarly. Indeed, carnivory in general (e.g., in bats, Santana et al. 2010) is associated with enhanced bite force (i.e., occlusive *F*2) at relatively large gape. However, for processing slippery prey (perhaps like nematodes for mites) piscivorous bats are morphologically distinct, with cranial shapes that enable high bite force at now narrow gapes.

Accordingly large strong chelae as in *Aleuroglyphus ovatus* AL2, *Chortoglyphus arcuatus* CH1, *Glycometrus hugheseae* G3, *Lepidoglyphus destructor* G6 and *Rhizoglyphus robini* R1 should be the best amongst the bird nest astigmatans studied for handling large nematodes on size alone. Trivially the force of a cut into a nematode is determined by accelerating the mass of the moveable digit to impact. Naturally, like for a sword (see J Clements, What Makes an Effective Sword Cut https://www.thearma.org/essays/howacutworks.htm), a stronger strike produces a better result. So *Aleuroglyphus ovatus* AL2, *Glycometrus hugheseae* G3, *Lepidoglyphus destructor* G6 and *Rhizoglyphus robini* R1 with a large input adductive force (*F*1) should cause the most damage (given similar *VR*). Of course, the process of muscular force production needs validation (as already done in bats, Santana et al. 2010).

Indeed amongst birds themselves, such as falcons (Tsang et al. 2019), it is the relative size of food material (i.e., prey in such raptors) that is related to talon form. Talons of species preying on relatively smaller prey were morphologically distinct from those of predators on relatively large prey, the former being characterised by a slender, less curved talon. Tool shape to prey size matching may occur. For comparative scaling therefore, *CLI* may be a better basis than body size, body mass (Pike and Maitland 2004) or idiosomal length *IL* (see Bowman 2024a) when comparing moveable digit abilities as tools in mites.

Mites with a digit asperity like the nosomial tooth in falcons (see Bowman 2025b), should be able to rely more strongly on the strike impact to subdue nematodes. This would apply to *Tyrophagus putrescentiae* (see Bowman 2024a, b). Indeed such features (like also strengthened digit tips) should, as Owen Seeman (*pers.comm.*) points out, be able "...to pull out, pick up and handle thread-like food..." such as also "...strands of fungal hyphae..." too, in that way possibly offering "...a good pathway to nematophagy for acarids...". As he further points out, pulling "...threads from a mass would have resistance..." but of course, fungi cannot ‘thrash’ to escape butchery!

Bird nest astigmatans with moveable digit peak zenith and gullet nadir angles that are small (i.e. narrow features, especially when $R_{t}$ is large, e.g., *Glycometrus hugheseae* G3 for sure and in part possibly *Glycyphagus domesticus* G5 and *Lepidoglyphus destructor* G6), are mappable to the capture design of ospreys whose highly recurved talons (like also in some eagles) are an adaptation for feeding on slippery prey e.g., fish. Highly curved claws in water-bugs catch proportionately larger prey than water-bug nymphs with less curved claws (Ohba and Tatsuta 2016). Short, strongly curved, ‘full-bodied’ claws in lizards are carried by climbing lizard species for grip (Baeckens et al. 2019). Curved claws are interpreted in the fossil record as evidence of scansoriality (Mann et al. 2021).

Large mites (e.g., *Rhizoglyphus robini* R1) should therefore be the best design amongst the bird nest astigmatans for gripping and dealing with large opportunistic (nematode) prey. In contrast, enlarged talons in birds with comparatively low curvature on each avian digit are part of a suite of adaptations to increase constriction efficiency by maximising grasp strength, indicative of specialisation on small prey. Small mites (e.g., *Dermatophagoides pteronyssinus* D3) amongst the bird nest inhabiting astigmatans may thus focus on small nematode consumption. How the mites’ legs may function to synergise these behaviours needs more observations in the wild.

Now, mites must ‘hit’ nematodes using their moveable digit lever arm (just like a sword) during capture, then suspensorially hang onto them and tenasorially grapple with them during any thrashing combat (especially if the nematode is large). The effectiveness of a sword thrust is determined by how resistant the target is at the point of impact (see J Clements, What Makes an Effective Sword Cut? https://www.thearma.org/essays/howacutworks.htm). So, using Bowman (2021), it makes just as much sense to talk about the mechanical advantage (= velocity ratio) dividing the prey load, instead of multiplying the adductive input force *F*1 (see George Turner, Part 2, https://www.thearma.org/spotlight/GTA/motions_and_impacts2.htm). This ‘resistance to acceleration’ inertia, is a measure of both the size of the load (or mass of the blade) and the dividing mechanical advantage of the lever.

Now, from mite to mite, moveable digit ‘swings’ can be assumed to be fairly similar in velocity profile, and digits optimised for the most common or most powerful swings, not for more obscure manoeuvres. So it is the target type that determines the optimal combination of inertia and edge velocity in any cut into the nematode at the mastication surface impact point. Just as baseball bats are not used to hit softballs, and softball bats are not used to hit baseballs, each chelal design will have been adapted to the impact dynamics of a particular nematode (here $\equiv$ the ball). Thus the choice of tip inertia of a moveable digit for any one astigmatan species would be adapted to optimise the impact dynamics of strikes against their preferred target nematode. Hitting ’hard nematodes’ needs a differently designed weapon than hitting ‘soft nematodes’. $\frac{F1}{VR}$ values suggest that *Aleuroglyphus ovatus* AL2, *Glycometrus hugheseae* G3, *Lepidoglyphus destructor* G6 and *Rhizoglyphus robini* R1 pack a hefty punch for their digit’s speed of closing, while *Acarus immobilis* A1 and *Acarus farris* A17 have the two weakest chelal designs (per speed of closing) for any potential nematophagy.

A plesiomorphic straight bar or rod-like moveable digit approximates a free-moving straight ‘fighting staff’ being propelled by the chelicera. Following the argument of George Turner, Part 2 https://www.thearma.org/spotlight/GTA/motions_and_impacts2.htm, the inertia at the tip of an untapered staff is always one quarter of the staff’s mass. The inertia at a point that is one third back from the tip is accordingly always three quarters of the mass, and as Turner explains - if you use the point $\approx 21\%$ back from the tip, where the impact point and pivot point are equidistant from the balance point, the inertia is exactly one half the mass of the staff. So, using his argument, if you have the impact point and pivot point equidistant from the balance point on any object, the inertia at the impact point is exactly one half of the mass of the object. Turner explains that this is because the impact naturally forms a lever with a two to one mechanical advantage, because the load is being positioned exactly in between the fulcrum and the effort. So, what mite features might be found if one divides the mastication surface by an 20 : 80 rule? This would be at approximately $x_{4} - x_{5}$ i.e., $\approx 21\%$ back from the moveable digit tip to the condyle in Bowman (2023a, 2024a, 2024b). This, for sure, is within the distal region where tooth height so dramatically increases over all bird nest astigmatan taxa (Fig. 15 upper). The staff analogy is a useful construct.

Furthermore, using his arguments in https://www.thearma.org/spotlight/GTA/motions_and_impacts2.htm, the amount of acarine digit mass that the target nematode reacts to would be just the blade’s inertia at the impact point, traveling with the given edge velocity of any sword-like blade. In deciding on the optimal impact location for the moveable digit, one is just choosing the location on the mastication surface that gives the best combination of inertia and velocity, since both vary all the way down the digit. The edge velocity obviously goes up linearly as the impact approaches the digit tip, since at the instant of impact the blade is actually rotating around the condyle (= the unique instantaneous centre of rotation). Indeed, George Turner, Part 2 https://www.thearma.org/spotlight/GTA/motions_and_impacts2.htm gives the basic shape of this curve which is the same for all objects. If you multiply the curve of the inertia by the edge velocity at each point you get the momentum, or quantity of motion, at each point along the cutting edge.

Using Turner’s argument, the latter is a straight line, sloping upward from the instantaneous centre of rotation. Then, the energy at each point along the edge is just one half of the inertia times the square of the edge velocity, with the maximum energy along the edge being always found at the impact point. However, as he explains, the point of maximum energy in a ‘normal swing’ (if it was a sword) is quite far back on the blade, often about one third from the ‘condyle’-equivalent, and is certainly not normally used as the striking point in practice. Against an extremely heavy target nematode, the moveable digit hitting on this point will completely stop the motion of the mastication surface, so the impact velocity is very low. Thus it is more likely to ‘bump’ the target (i.e., to repercuss), than to cut it. You would only strike prey with the point of maximum energy when you are using a digit to move heavy material, and even then, the mite’s gnathosoma would expect some tremendous equivalent to ‘hand shock’ (aka vibration). Indeed, consiliently one does not normally use your molar teeth to ‘strike’ foodstuff.

Using the inertia and velocity profile down the digit, it is therefore straightforward using Turner’s argument (in https://www.thearma.org/spotlight/GTA/motions_and_impacts2.htm), to visualise the effects of collisions (i.e., ‘damage’) with various target prey, which take into account their elasticity and impact velocity. Indeed, a sword-like moveable digit is not a very efficient tool. Most of the mass is in the basal ramus, which remains in motion. Heavy targets are best struck closer to the basal ramus, light targets closer to the tip. This is because a light target accelerates very easily, making it hard for the blade to build up cutting forces. To counteract this tendency, one needs all the edge speed one can get, and that is maximum at the digit tip. If the chela is approaching the nematode (like the target in a cavalry charge), the optimum damage is in fact done much further back on the blade (cf. the importance of the ascending ramus Bowman 2024a, b). Whereas if you are striking at a retreating target, as with a ‘backwards draw-cut’ from a retracting chelicera (i.e.,‘hook-and-pull’ like), it is the digit tip that will deliver the most damage.

From simple physics, the elasticity of a collision between an astigmatan moveable digit and a target nematode goes down with the ‘approach’, or ‘closing velocity’ of the advancing chelicera. A ‘slow crash’ just makes the digit and prey bounce off each other, being perfectly elastic. At higher speeds the collision will become very inelastic, and one would end up with a mangled mass of twisted nematode tissues. This behaviour tends to move the optimal impact point somewhat more towards the digit tip, where impact velocities are higher. So in high speed skimming of fluids (as probably in *Carpoglyphus lactis*, Bowman 2024a, b) the digit tip is where the ‘action’ is. In short, the determination of exactly the best point on a blade to strike with, is heavily dependent on external factors, not necessarily related to just the design of the blade. As Turner’s analysis shows, each collision has to be considered on a case-by-case basis, but one can draw some generalities from experience with Medieval, Renaissance, fencing and cavalry blades - "...Strike near the tip (in what is called the ‘Schwech’) to about a third back from the tip...". Moreover ‘thump’ the adversary (= nematode) with the sword and hilt near your hand (= condyle).

*For the ‘expert predatory mite’* - What chelal digit is best for chopping off completely the prey’s head or its arm or leg?

Following Francisco Urbano Garci (https://www.thearma.org), both the straight and curved moveable digit blades should be the same in this respect. The curved blade will go deeper into the nematode at the beginning since its shape allows part of the blade to ‘wait outside’ the prey’s body before starting cutting. One could say that the curved blade has a ‘thrusting’ power at the beginning of the cut which the straight blade has not. However, the straight blade will cut wider, and all the advantage that the curved blade had to ‘thrust in’, when the cut reaches the middle point, then becomes a disadvantage to keep cutting. Using Garci’s argument, by the middle point, the straight blade has done most of the job and past the middle point will cut the nematode easier than the curved digit blade. So matters equal out.

*For the ‘expert predatory mite’* - What chelal digit is best for deep cuts into prey?

Again, following Francisco Urbano Garci (https://www.thearma.org), since both straight and curved blades will cut the same area, although the straight blade will do wider cuts, due to the shape of the curved blade the curved digit will go deeper. As in humans the deeper a wound the deadlier it becomes, so a narrow deep cut is more life-threatening to a nematode than a wide shallow one. Therefore, for deep cuts the curvier the digit blade the better (c.f. bird nest astigmatans with high values of the *ogcar* measure: *Chortoglyphus arcuatus* CH1, *Dermatophagoides pteronyssinus* D3, *Glycometrus hugheseae* G3 and *Lepidoglyphus destructor* G6).

In fact, if one tries to cut a cylinder (like a nematode) deep to its centre, experience with weapons (explained below) shows that it will take approximately $28\%$ more energy to do so with a straight blade than that with a moveable digit itself as curved as that cylinder itself (i.e. very curved - or more than just like a scimitar or cutlass). The $28\%$ arises from considering how much resistance a cylindrical nematode will offer to a curved blade against a straight blade as follows:

Let us assume that both blades go as deep as each other to the centre of the cylinder. From the above argument by Francisco Urbano Garci (https://www.thearma.org), one only has to pay attention to the area cut by the blade in order to calculate the resistance it will get from the target tissue. Assume that the curved blade is as curved as the nematode’s body (i.e., matched and very curved to enhance grip). Then, if both blades go to the centre of the cylinder (with a radius, *rad* which is of one arbitrary unit) one has the following amounts of area removed according to Garci:

$$Straight\ digit = \frac{\varPi }{2}$$

$$Curved\ digit =\frac{\varPi }{2}-2.\left[ \int _{0}^{\frac{\sqrt{3}}{2}}\left( 1-\sqrt{1-x^{2}}\right) dx+\int _{\frac{\sqrt{3}}{2}}^{1}(\sqrt{(1-x^{2})}dx\right]$$

Or (for $rad=1$): $Straight = 1.5708$ units, $Curved = 1.2284$ units and therefore one will have to place $\approx 28\%$ more energy into a straight blade cut to go as deep as the curved blade (according to Garci). Clearly matching the curvature of the digit blade to the curvature of the preferred nematode body size would have a physical rationale as an evolutionary selection pressure. So, given the standard result that the curvature of a circle is $\frac{1}{radius}$, a linear segment along the *L*2*M* axis of size *z* for say astigmatan species Q maps (for a circular digit ‘blade’) to a curvature of $\frac{2}{z}$ which should match the curvature of the optimal nematode size for attack by species Q.

However, importantly, if one wants to cut *completely through* the cylindrical nematode, from the middle point on, the straight blade for the latter function has an "... easier life that the curved one..." (see the ‘chopping off’ argument above). So the values ‘even out’ resulting in having to use the exact (same) amount of energy with both blades to completely cut the worm (cf. the ‘cutting the head off argument’ above). Certainly curved blades would be preferable in fights against an essentially square-sided nematode but it does not matter much against more rounded prey (see argument by George Turner, Part 3, https://www.thearma.org/spotlight/GTA/motions_and_impacts3.htm). Once the edge has penetrated through any armoured skin, the muscle inside should be cut quite well by either type of mite blade.

Note that a non-circular curved blade like one matching a *log* spiral shape (defined generically as $x=q.cos(\theta ), y=q.sin(\theta ), q=a.e^{k.\theta }$) has the advantage of maintaining a constant angle of penetration such that it embeds itself further into the substrate or prey item during flexion and raking motions (as anchors do - which dig themselves into the sea floor when dragged, Thomson and Motani 2021).

*For the ‘expert predatory mite’* - What chelal digit is best for cutting nematodes with *just* its moveable digit ‘pointy’ tip?

In this case, the curved blade will follow easily the circular movement around the condyle and will not try much to go deeper into the body, making the slicing cut really easy. Of course it may ‘miss’ the tubular prey target for a part of that trajectory. With a straight blade, the point will try to go deep inside the nematode’s body which might cause the blade to stop at the middle of the cut (due to tissue resistance). However from the above arguments, the deeper the cut, the deadlier the stroke. Besides, if the digit blade stops it would be a great opportunity for the astigmatan to start a thrusting attack by the whole chelicera (or even the whole gnathosoma/idiosoma) being moved forward.

*For the ‘expert predatory weapon designer’* - What about ‘draw-cuts’?

As alluded to above, draw-cuts could be considered as a part of a ‘gryporial hook-and-pull’ tactic for digging into foodstuff by the chela. Cutting effectively with any edged weapon is not identical to hitting it with a blunt stick or club. As George Turner, Part 3 https://www.thearma.org/spotlight/GTA/motions_and_impacts3.htm recounts, there are some interesting things going on when slicing, part of which is the sawing or ‘dragging’ action of a blade. For a mite, this will primarily benefit cuts against soft and sticky food targets (or prey) that have strands of connective tissue, like meats. Turner explains, that draw-cuts are not quite so effective against things like wood or metal, where the stiffness of the material maintains firm resistance against the sides of the blade (in its kerf), instead of just the edge. Against foodstuffs behaving like any metal, an acarine digit draw-cut will work only by abrasion. Indeed, consiliently one does not try to cut metal by using knives as reciprocating saw blades, because it would take hours to make such a cut. Chitin (around prey) may mechanically behave just like this.

It is worth pointing out that sharp blades do not ‘stick’ nor do prey muscles ‘clamp down’ or contract tightly upon being cut (see J Clement, What Makes an Effective Sword Cut? https://www.thearma.org/essays/howacutworks.htm). A blade quickly chopping or cleaving through flesh moves too fast for this sort of thing. Flesh is very elastic and largely made up of water (as are nematode tissues) and while there is afterwards a ‘physiological shock’ reaction that helps prevent fluid loss, this would not prevent the forceful edge blow of a sharp bladed sword-like moveable digit from being withdrawn from nematode tissues (or any other such material).

Overall one assumes that unless the nematodes are really small when being hit by the moveable digit, no predator expects to cut the ‘victim’ directly in two but simply to create a damaging deep cut and then imbibe the emanating tissue fluids. To that end, therefore curved moveable digit blades (like a marine officer’s sabre, see https://upload.wikimedia.org/wikipedia/commons/0/0b/MuseeMarine-sabreOfficer-p1000451.jpg) are probably better for this. Moreover, the advice for the predator is to strike with the percussion (impact) point, which is always about a third of the weapon’s length back from the tip (i.e., at an oscillation node from ‘slapping’ the blade or around $x_{6}$ in Bowman 2023a, 2024a, b), just as fencing masters and cavalry manuals tell us (George Turner, Part 3, https://www.thearma.org/spotlight/GTA/motions_and_impacts3.htm). This is where the moveable digit blade should be the sharpest. Indeed, for the 18 semi-landmarks used, $x_{6}$ is in the intercalated teeth area (recall that the tooth row stops around $\approx x_{10}$ to $x_{12}$ Bowman 2025b). Figure 7 shows consiliently that for every taxon (bar *Dermatophagoides pteronyssinus* D3) the teeth here *are* sharper than those proximally (note that the most distal teeth are for ‘hooking’). Of course moving the centre of gravity back towards the condyle has almost no noticeable affect on the location of this ‘node of vibration’.

*For the ‘expert predatory mite’* - Do free-living bird nest astigmatan mites cut material uniformly?

That is, do they cut foodstuff into slices of predefined thickness ($\equiv$ weight) as in industrial food processing machines (Bremer and Matthiesen 2020). A clear theory here appears lacking. Such a process "...is usually adjusted by a trial and error method and based on experience..." and "...blade geometries have been defined pragmatically in industry so far...", although an involute sickle-like form has been used (Bremer and Matthiesen 2020). The involute function is the shape of phytoseiid digits (Bowman 2025b). As Bremer and Matthiesen (2020) says "...The increasing radius of the sickle also creates a slicing motion, which leads to reduced cutting forces compared to cutting by pushing only...". Indeed, they point out that, a blade with a geometry of changing curvature alters the ‘slice to push’ ratio and reduces cutting forces at the start and end phases. Note that Bowman (2025b) found that the moveable digit ventral surface intrinsic curvature appeared to be common across the free-living bird nest astigmatans (although the surface appears visibly variably curved).

Further, Bremer and Matthiesen (2020) continue "...Toothed blades, such as serrated blades, are also used for slicing. A variety of tooth forms is available for slicer blades. Reasons underlying the selection of the tooth form are unknown..", however "...toothed blades are used for less stiff foods to reduce deformation and improve positioning on the conveyor...." and toothing is used to change the orientation of the blade surface relative to the cutting plane in order to reduce the adhesive contact between blade and slices...". All this pertains to how astigmatan digits might work (e.g. discrete teeth for wetter material?). Indeed, how might food fragments be accelerated into the mite mouth and the digits cleaned (both sides) during feeding by virtue of their geometry? Bremer and Matthiesen (2020) introduces (their Fig 3) three new slicing blade angles (in a tangential direction to the blade curvature) to consider: pressure angle, free angle, and blade angle. How do these relate to mastication surface angles in mites?

Combining the asperity degree of slope and set (Bowman 2024a) determines the first of these two ‘new’ angles which not only relate to slicing efficiency but also to the induced lateral ‘peeling away’ of cut material (see Fig. 2 of Bremer and Matthiesen 2020). For mites this movement ought to be intrachelicerally towards the mouth, so such blades should be mirror images of each other on the left and right chela. This needs verifying in mites. For the third ‘new’ angle, the symmetric blade angle value leads to a deformation of food material on both sides of the blade resulting in symmetric cutting forces. A sharp blade angle of $20-33^{\circ }$ "... can reduce sticking of slices to the blade...". Note from Bowman (2025b) that moveable digit tip angles are typically approximately twice this. Bremer and Matthiesen (2020) continue that "...The pressure angle of the slicer blade influences its mechanical stiffness. From above, the less and less acute the blade angle the more and more percussive ‘thumping’ and tearing of material..." would occur. Bremer and Matthiesen (2020) also claim that an "...asymmetric cutting angle also causes a deformation of the slice only..." and facilitates "...cutting thin slices..." of material. Bremer and Matthiesen (2020) finally state that "...The free angle is to reduce contact between blade surface and the newly cut food surface...".." and "...On the other hand, acceleration of the slice towards the conveyor is influenced by the blade angle. For this reason, blades are designed with blade angles that vary over the blade curvature to achieve different accelerations of the slice during the cutting process...". All this fine detail pertains to how astigmatan digits might work. Much investigation via 3D micro-tomography of mite dentition working is needed.

### Conundrum (iv)—What might some asperity angles seemingly not to be very different between bird nest mites and other species be indicating?

For sure, canine morphology in felids varies across species reflecting differences in (prey) killing mode (Christiansen 2007). Similarities amongst such vertebrates appear related to their phylogeny. Herein for the studied astigmatans, the distal (first) tooth appears fairly uniform across the free-living mites pointing to a simple common ‘piercing’ function on chelal occlusion (or a common slicing or scalporial scratch-digging function on cheliceral retraction). Note that amongst them *Acarus farris* A17 is the most blade-like design distally. With respect to hook-and-pull, the distinctly different distal (first) gullet nadir angles in *Chortoglyphus arcuatus* CH1, *Glycometrus hugheseae* G3, *Lepidoglyphus destructor* G6, *Dermatophagoides pteronyssinus* D3 (and possibly *Acarus gracilis* A4) suggest these represent different more ‘pocketed’ designed gryporial tools. The variation in proximal (last) tooth zenith angle suggests that (using *Tyrophagus palmarum* T17, *Tyrophagus palmarum* T32, *Tyrophagus similis* T21 and *Tyrophagus similis* T44 as central undifferentiated exemplars), there is clear evidence of a different ‘blade for stripping’ design in *Dermatophagoides pteronyssinus* D3 (and perhaps a sharper ‘gripping for stripping’ design in *Acarus gracilis* A4, *Glycyphagus domesticus* G5, *Lepidoglyphus destructor* G6) at high effective velocity ratio positions. This ‘stripping’ differentiation for gleaning is confirmed by the shallow nadir angled proximal (last) gullet in *Dermatophagoides pteronyssinus* D3, the very steep angled proximal (last) gullet nadir angle in *Glycyphagus domesticus* G5 (and to a lesser extent in *Tyrophagus putrescentiae* T13 confirming the ‘stripping’ conclusion for this species in (Bowman 2024b). There is also mild evidence that *Acarus immobilis* A1 may have a specialised pocket here possibly too. Fine distinctions thus matter between bird nest astigmatans.

### General summary for free-living bird nest astigmatans

There is not an unambiguous one-to-one relationship between mastication structures and trophic functions in free-living bird nest astigmatans. Indeed resource partitioning arguments can be difficult to deploy when faced with ‘generalist’ species. Astigmata moveable digits approximate various different bladed tools (e.g., Duckbill lead snips, Tin snips, Monument lead snips etc., https://www.blackburntools.com/articles/rose-tools-catalog-archives/index.html) with varying degrees of underbite (see *Terrogynium weatherwaxae* chelicera in Bowman 2025b) or indeed overbite. Some glycyphagids have saw-like moveable digits.

**Fig. 29:**
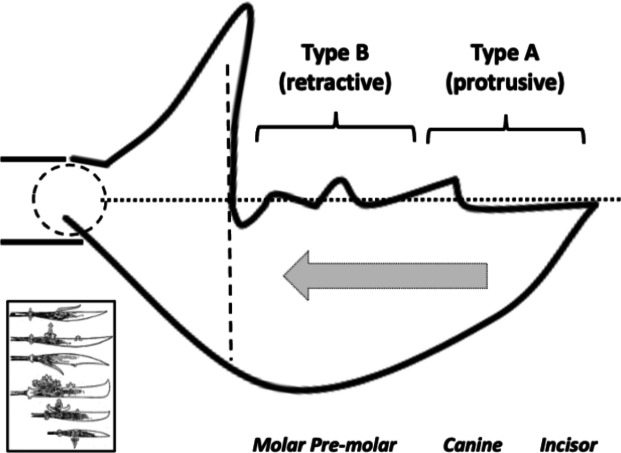
Schema of typical ‘glaive-like’ bird nest astigmatan composite moveable digit to scale (anterior to the right). Inset box = various glaives amended from Boeheim (1890) - public domain image from https://en.wikipedia.org/wiki/Glaive. Mite basal ramus (stylised dorsally like the weapon is), ascending ramus, three teeth, digit tip and ventral horizontal ramus all highlighted with black line. Pole-arm to the left (at approximate location of dashed line condyle). Dotted line = reference *L*2*M* adductive output lever arm. Teeth drawn with typical rake and back (so thus typical zenith and nadir) angles for that region. The three teeth (left to right) then the digit tip could be colloquially labeled with the vertebrate dental terms: ‘molar’, ‘pre-molar’, ‘canine’, ‘incisor’. They form half of a gripping ‘socket’. The distal (‘canine’) tooth on average is usually ATB-like (probability $\approx 80\%$), thereafter posteriorly for the intercalated ’pre-molar’ and proximal ‘molar’, there is an $\approx 50:50$ chance for the tooth to be FT (or blade-like) instead. Black vertical dashed line = end of tooth row ($x_{i_{e}}$) at approximately *L*1*U* units from the condyle along the *L*2*M* reference axis. Region ‘Type’ annotated with cutting/hooking action on cheliceral movement (note that the overall ‘gross’ Type will vary depending upon the actual maximum peak and minimum valley values over all *L*2*M*). Grey block arrow is anterior$\rightarrow$posterior gradient explained in the text

Typically there are $\approx 3$ (range $1-6$) teeth and therefore 4 (range $2-7$) gullets between the moveable digit tip and the ascending ramus, together approximating the design of a torque-wrench ‘socket’ (Fig. 29). Strong species differentiation is seen in both. The mastication region overall is an ‘easy-to-start-moving’, non-tearing, well-controlled cutting tool with gently shaped ‘attack surface’ teeth as: proximally (to the condyle) mean tooth rake angles $=-48^{\circ }$, distally $=-70^{\circ }$. and proximally (to the condyle) mean tooth back angles $=+33^{\circ }$, distally $=+43^{\circ }$. Blunt teeth in general are deemed to be "... important to avoid tooth breakage and increase the contact area with the food..." Herrel and Holanova (2008). Teeth become progressively blunter distal$\rightarrow$proximal (grey block arrow in Fig. 29). Average tooth zenith angles of $93^{\circ }$ ($81^{\circ }$ distal, $103^{\circ }$ proximal) suggest an adaptation to slice through stiff, clay-like semi-wet, loose aggregate granular material. Indeed blunt and shallow features (i.e., ‘pads’) may be suitable for contact with and the gripping of wetted material. Furthermore, such "....compliant materials have large coefficients of friction, making it possible to use lower gripping forces...." Cutkosky and Wright (1986). Larger zenith angles values in some bird nest astigmatan species approximate those of hand-axes to chop and split material (or even ‘scraping teeth’ in fish, Table 2). Gullets usually become more ‘pocketed’ (grey block arrow in Fig. 29) i.e., sharpened progressively distal$\rightarrow$proximal (with some blunting exceptions and a higher degree of variability within and between species than teeth). Gullet nadir angles at $103^{\circ }$ on average are greater than tooth zenith angles. They suggest an adaptation to catch upon or scoop aggregate granular material or scrape off detrital ‘debris’.

Free-living bird nest astigmatans typically have a default distal Type A (tearing) moveable digit design (cf. Bowman 2024a) with a distal claw-like hook comprising of a $\approx 115^{\circ }$ gullet posterior of the digit tip followed by a sharp ($\approx 80^{\circ }$) tooth. Proximally most species have a standard sized blunt saw blade-like profile in a gross Type B (nibbling) arrangement. The zenith and nadir angles encompass those of ‘tube-’ or ‘tile-cutters’ and known durophages (Table 2). An earliest apomorphic bidentate (i.e., tri-gulleted) form (comprised of incisor tip, canine and molar equivalents) is proposed arising from a plesiomorphic bar. Usually, the ($\ge 1$) intercalated (pre-molar) teeth are blunter than the distal ones but sharper than the proximal ones (grey block arrow in Fig. 29, i.e, there is a gradient posteriorly, teeth also getting blunter and blunter with more intercalation). Usually intercalated gullets are more ‘pocketed’ than distal gullets. However, thereafter posteriorly the picture is variable across species. Moveable digit gullet variation is where the focus of trophic differentiation design is once teeth are present. Gullet nadir angles $\approx 100^{\circ }$ indicate a grasping role to hang onto or ‘climb-over’ foodstuff asperities. A formal split-plot analysis confirms different patterns of gullet differentiation and thus ‘detritus’ handling between species.

ATB-like teeth have on average zenith angles of $84^{\circ }$. FT teeth have on average zenith angles of $102^{\circ }$. They form a potentially useful feature to score taxonomically. The distal tooth on average is usually ATB-like (probability $\approx 80\%$), thereafter posteriorly there is an $\approx 50:50$ chance for a tooth to be FT instead. The sequential ordering of ATB versus FT along the digit differs between species. Repeated FT teeth could be considered as blades. Tooth peaks are separated on average by $4.1\mu$m but this is variable with mastication surface region. Gullet to next gullet spacing is $\approx 4.2\mu$m but it is also regionally variable. The position of the first (‘hook’) gullet behind the moveable digit scales positively with body size (*IL*). The pattern of tooth and gullet spacing varies by species. Extra intercalated teeth and extra intercalated gullets are at ever diminishing distances posteriorly. An anterior$\rightarrow$posterior orientation to moveable digit design evolution is posed.

Stochastically the mastication surface of the moveable digits of bird nest astigmatans map between International Roughness Grade Numbers N5 to N7 suitable for fine grinding. Effective penetration by asperities into foodstuff is on average $1.8\mu$m and scales positively with body size. The maximum rising and falling of the mastication surface varies across species and moveable digit regions. Gullets drift ventrally downwards as one moves posteriorly (grey block arrow in Fig. 29) accentuating a shift to a more ‘nibbling’ Type B-like overall design. Bar the first (post-tip) distal tooth, intercalated and proximal teeth on average decline in height posteriorly (grey block arrow in Fig. 29). Usually gullets deepen posteriorly (grey block arrow in Fig. 29). The scale of food penetration matches the degree of chelicerisation. The moveable digit proximal mastication surface region is decoupled from chelal robustification. Work by the teeth increases posteriorly. Robustification has the largest adaptive impact in the intercalated (pre-molar) tooth region.

Cutting and crushing style chelae are found, some suitable for adventitious nematophagy. Digit tips matter. Sharper teeth and more pocketed gullets tend to be closer together. Typically most species carry an approximately Gaussian mastication surface. However size matters. Larger tooth row larger mites have a more fluctuating or a more differentiated composite mastication surface. Some species mastication surfaces are not Gaussian in nature suggesting concerted evolution into functional ‘modules’ for such digits. The moveable digit surface neither overall ‘crinkles up’ nor ‘flattens/smooths out’ on lengthening of the mastication surface, rather asperity angles are conserved on digit extension/shrinkage. However, changes are not concerted over regions, although they may be concerted within ‘modules’. Digit thickening or thinning on increased tooth row length varies across species. Most free-living bird nest astigmatans grossly, somewhere along their moveable digit, are Type B ‘nibblers’ catching onto material on cheliceral retraction (regionally ‘contradicting’ the default distal Type A architecture but functioning as a ‘hook’ similarly). Claw length equivalents for moveable digits were around $9\mu$m. Overall hook angles ($\gamma$) were on average $=112^{\circ }$ for gross ‘tearing/chewing’ Type A designs, $90^{\circ }$ for gross ‘nibbling’ Type B designs. Various patterns of being ‘open-faced’ or ‘pinched face’ variants of gross Type A and gross Type B designs were found. There was some evidence that any larger overall mastication surface fluctuations need to be further apart i.e., the original small mites were probably trophically undifferentiated. Size is important to feeding differentiation.

Returning to the four conundra of: *Acarus immobilis* A1, *Tyrophagus palmarum* T17 and *Tyrophagus similis* T21 being in the same surface fragmentary feeding class; *Aleuroglyphus ovatus* AL2, *Chortoglyphus arcuatus* CH1, *Glycometrus hugheseae* G3, *Glycyphagus domesticus* G5 being in the same particular interstitial omnivore class; *Acarus gracilis* A4 and *Tyrophagus putrescentiae* T13 being together in one specific interstitial fragmentary feeding design class, and *Tyrophagus palmarum* T32 and *Tyrophagus similis* T44 being together in another specific interstitial fragmentary feeding design class, one concludes...

The above paragraphs in the Results and Discussion explain how *Acarus immobilis* A1, *Acarus gracilis* A4, *Chortoglyphus arcuatus* CH1, *Glycometrus hugheseae* G3, *Glycyphagus domesticus* G5, *Tyrophagus putrescentiae* T13 are trophically distinct (and by inference *Aleuroglyphus ovatus* AL2 too) using mastication surface features.

However, the ability for *Tyrophagus palmarum* and *Tyrophagus similis* variants to coexist without direct trophic competition in the bird nest habitat is unclear. These species both represent a very general ‘central’ undifferentiated trophic design for a fragmentary feeding variously sized acarid. Conundrum (iv) is unresolved by trophic morphology. Perhaps these two species’ feeding behaviour is at different substrate heights like that suggested for herbivorous dinosaurs Mallon et al. (2013). Indeed, just how airorhynchid or klinorhynchid is their feeding strategy in the wild? Perhaps resource partioning (Begon and Mortimer 1981) is achieved by them being preferentially attracted to different parts of the nest habitat for other reasons than foodstuffs? Something is different for *Tyrophagus similis* since as Owen Seeman (*pers.comm.* points out this species "... is well known for attacking young plants (like spinach)...." (which *Tyrophagus palmarum* seemingly does not).

### Specific summary for each bird nest astigmatan species

This will be framed in the family, genus, species order of Table 1. Within species dental polymorphisms occur. Detailed points may be abbreviated and a summary offered gross-referenced to Fig. 29. The functional spaces classification scheme of Qin et al. (2023) (i.e., piercing, gryporial hook-and-pull, and scalporial scratch-digging) may be used.

#### *Acarus farris* A17

*Acarus farris* A17 is the most plesiomorphic moveable digit in that it has the shallowest distal gullet and blunter distal teeth that the other species. *Acarus farris* A17 may possibly show alternating tooth sharpening and blunting of intercalated teeth as one moves along the mastication surface. *Acarus farris* A17 is noticeably heterodontous and has the highest heterogeneity of (small) gullets, suggesting random small scale (measurement) variation around a bar-like form. This species has noticeably flatter FT teeth with average zenith angles $\approx 115^{\circ }$. *Acarus farris* A17 shows a degree of differentiation proximally compared to a basis form. The $\gamma$ ‘claw’ angle is more obtuse in this species giving a more ‘open face’ to its design. In having blunt asperity regions following blunt asperity regions this species is the most bar-like in general form. The relatively unornamented fairly long digit is rather like *C.lactis* (see Bowman 2021, 2024a, b), so feeble piercing/gripping, fluid ‘skimming’ and ‘picking’ of foodstuffs is indicated. *Acarus farris* A17 (along with *Suidasia pontifica* S5) has the smoothest potentially fine-grinding action mastication surface. Consiliently, the moveable digit disproportionately thickens on digit length increase (thus becoming more ‘crushing’ style adapted).

#### *Acarus gracilis* A4

*Acarus gracilis* A4 usually showed a gross Type A hooking/tearing design adapted for protrusion (piercing and slicing) into foodstuff. The surface teeth as a set are like other *Acarus* spp.. *Acarus gracilis* A4 (like *Tyrophagus putrescentiae* T13) has more intercalated teeth than other bird nest astigmatan species (except the multi-dentate *Glycyphagus domesticus* G5 and *Lepidoglyphus destructor* G6). There is some evidence of intercalated gullet sharpening as more gullets are intercalated too. This all indicates not just gryporial adaptations but also a grinding saw design. Again the moveable digit disproportionately thickens on digit length increase (thus becoming more ‘crushing’ style adapted).

#### *Acarus immobilis* A1

*Acarus immobilis* A1 shows morphological specialisation to grip, tear and chew small objects distally. The $\gamma$ ‘claw’ angle is particularly more obtuse in this species giving a strongly ‘open face’ to its design. However, the more acute proximal $\gamma$ ‘hook’ or ‘claw’ angle suggests a ‘nibbling’ specialisation here for this species. The surface teeth as a set are like other *Acarus* spp.

#### *Aleuroglyphus ovatus* AL2

In having blunt asperity regions following blunt asperity regions this species, on the face of it, is the most bar-like in general form. The surface teeth as a set are bladed and *Aleuroglyphus ovatus* AL2 has teeth on average with the highest penetration into foodstuff ($3.2\mu$m). Distally, there is a large initial rising first tooth in this species. Intercalated teeth also show large rising and falling ($R_{t}$) values in *Aleuroglyphus ovatus* AL2. However, proximally the mastication surface in *Aleuroglyphus ovatus* AL2 shows a rather feeble design reflecting the change in gullet depth as one moves posteriorly being variable in this species. The $\gamma$ ‘claw’ angle is more acute in this species giving a more ‘pinched face’ to its gross Type A tearing/chewing design. The more acute proximal $\gamma$ ‘hook’ or ‘claw’ angle suggests a ‘nibbling’ specialisation here for this species. *Aleuroglyphus ovatus* AL2 is morphologically specialised to grip, tear and chew large objects. Indeed the teeth of this species do much more (penetrative) work on chelal occlusion compared to most other species. As the hypothesis of a Gaussian surface is rejected for this species, there are not independent fluctuations along the reference axis *L*2*M* i.e., concerted adaptations (into ‘modules’) are present. It is a powerful crushing species. The mastication surface in this species has also, compared to most other bird nest astigmatans, coarser and more rasping properties.

#### *Rhizoglyphus robini* R1

This (often bulb-feeding) species has a distinct design. *Rhizoglyphus robini* R1 has the longest distance from digit tip to the first gullet (in line with its large size, *IL*). Distally, there is a large initial rising first tooth in this species. This long distal gryporial hook-and-pull design is promptly followed by multiple teeth ($\equiv$ enhanced Type A design). The change in gullet depth as one moves posteriorly is variable in this species. Compared to most other species, the teeth of this species do a little bit more (penetrative) work on chelal occlusion than the norm. Uniquely amongst the bird nest astigmatans ATB-like teeth tend to alternate with FT teeth suggesting a particular type of ripsaw (or certain cross-cut saw) design like that used for the rough sawing of wooden lumber.

#### *Thyreophagus entomophagus* TH3

*Thyreophagus entomophagus* TH3 has a long distal hook promptly followed by multiple teeth ($\equiv$ enhanced Type A design). The change in gullet depth as one moves posteriorly is variable in this species. It is not very common to have intercalated teeth in this species.The surface teeth as a set have a tendency to form runs of the same type. Unusually for a large mite, the moveable digit disproportionately thins on digit length increase (thus becoming more ‘cutting’ style adapted). Could this be an opportunistic facultative predator? It "...can be reared on brewer‘s yeast..." and has been found "...on plants in agriculture..." (Owen Seeman *pers.comm.*) although Klimov et al (2023) suggests it likes secluded habitats (like under bark - and thus nests too).

#### *Tyrolichus casei* T62

No individual showed any chelal underbite. The mastication surface of *Tyrolichus casei* T62 is furnished with relatively short, stout teeth capable of sustaining repeated loads. All individuals showed a gross Type B (nibbling design) suitable for foodstuff cutting/slicing on cheliceral retraction. However, the $\gamma$ ‘claw’ angle is slightly more acute in this species giving a rather ‘pinched face’ to its gross Type A tearing/chewing design. The more acute proximal $\gamma$ ‘hook’ or ‘claw’ angle suggests a ‘nibbling’ specialisation here for this species. The mastication surface in this species has, compared to most other bird nest astigmatans, coarser and more rasping properties. As a hypothesis of a Gaussian surface is rejected for this species, there are not independent fluctuations along the reference axis *L*2*M* i.e., concerted adaptations (into ‘modules’) are present. The surface teeth as a set alternate in type. Compared to most other species, the teeth of this species do a little bit more (penetrative) work on chelal occlusion than the norm. Could this be a rasping chewer and excavator of foodstuffs (after all it is often found associated with cheese)?

#### *Tyrophagus longior* T40

The $\gamma$ ‘claw’ angle is more obtuse in this species giving a more ‘open face’ to its design than the default bird nest astigmatan Bauplan. In this species the proximal ‘pocket’ becomes much bigger as the chela becomes more robust suggesting a type of power-gleaning design for this known plant pest (of cucumbers, Buxton 1989).

#### *Tyrophagus palmarum* T17, T32

An unremarkable form. The surface teeth as a set are like the three *Acarus* spp. in *Tyrophagus palmarum* T32. The moveable digit disproportionately thickens on digit length increase in *Tyrophagus palmarum* T17 (thus becoming more ‘crushing’ style adapted).

#### *Tyrophagus putrescentiae* T13

*Tyrophagus putrescentiae* T13 shows a degree of differentiation proximally compared to a basis form. Indeed proximally this species’ tooth back angle is markedly polymorphic. *Tyrophagus putrescentiae* T13 is a heterodontous outlier in terms of variation in tooth zenith angles. *Tyrophagus putrescentiae* T13 (together with *Glycometrus hugheseae* G3 and *Lepidoglyphus destructor* G6) also have the most ‘pocketed’ gullets (nadir angle $\le 93^{\circ }$) in general. Notably *Tyrophagus putrescentiae* T13 has a strongly pocketed proximal gullet (i.e., after the last most posterior tooth) suitable for ‘stripping’. The $\gamma$ ‘claw’ angle is more obtuse in this species giving a more ‘open face’ to its design. *Tyrophagus putrescentiae* T13 also has the flattest ATB-like teeth with a zenith angle on average $=95^{\circ }$.The surface teeth as a set have a tendency to form runs of the same type. *Tyrophagus putrescentiae* T13 (and *Acarus gracilis* A4) also have more intercalated teeth than other bird nest astigmatan species (other than the multi-dentate *Glycyphagus domesticus* G5 and *Lepidoglyphus destructor* G6). Even amongst the intercalated teeth, there is a posterior trend for ‘latch-like’ pocketing. The same specialisations as in previous studies (Bowman 2023a, 2024a, b, 2025b) were found. *Tyrophagus putrescentiae* T13 is confirmed in being able to catch onto, grab and strip larger and larger hyphae in relation to increases in chelal size. It may represent a composite ‘Swiss Army Penknife’ of free-living astigmatan designs able to handle all sorts of detritus. Despite its generalist habit it is designed uniquely.

#### *Tyrophagus similis* T21, T44

An unremarkable form. It is not very common to have intercalated teeth neither in *Tyrophagus similis* T21 nor in *Tyrophagus similis* T44. Both *Tyrophagus similis* T21 and *Tyrophagus similis* T44 show a more ‘open faced’ nibbling design than in other species. The surface teeth as a set are bladed.

#### *Chortoglyphus arcuatus* CH1

*Chortoglyphus arcuatus* CH1 usually showed a gross Type A hooking/tearing design adapted for protrusion into foodstuff. However, *Chortoglyphus arcuatus* CH1 had a reduced scale of distal rake to its teeth (i.e., $-56^{\circ }$ compared to the typical $-70^{\circ }$). This species distally has a more saw-like nibbling Type B mastication surface design. Post-tip there is a large drop in *Chortoglyphus arcuatus* CH1 into the first gullet. Furthermore, distally there is a large initial rising first tooth in this species. Intercalated teeth also show large rising and falling values in *Chortoglyphus arcuatus* CH1. The change in gullet depth as one moves posteriorly is variable in this species. The surface teeth as a set are blade-like. Compared to most other species, the teeth of this species do much more (penetrative) work on chelal occlusion. The $\gamma$ ‘claw’ angle is more acute in this species giving a more ‘pinched face’ to its gross Type A tearing/chewing design. The mastication surface in this species has, compared to most other bird nest astigmatans, coarser and more rasping properties. As a hypothesis of a Gaussian surface is rejected for this species, there are not independent fluctuations along the reference axis *L*2*M* i.e., concerted adaptations into ‘modules’ are present. *Chortoglyphus arcuatus* CH1 is highly derived distally. Indeed, *Chortoglyphus arcuatus* CH1 is distinctly trophically specialised not just to hook larger objects but it is also morphologically specialised to grip, tear and chew those large objects.

#### *Glycometrus hugheseae* G3

*Glycometrus hugheseae* G3 had a reduced scale of distal rake to its teeth (i.e., $-56^{\circ }$ compared to the typical $-70^{\circ }$). It is not very common to have intercalated teeth in this species. *Glycometrus hugheseae* G3 (with *Lepidoglyphus destructor* G6) has the sharpest zenith angle for its ATB-like teeth on average at $\approx 73^{\circ }$. This species has noticeably flatter FT teeth (blades?) with average zenith angles $\approx 115^{\circ }$. Distally, there is a large initial rising first tooth in this species. Intercalated teeth show large rising and falling values in *Glycometrus hugheseae* G3. The surface teeth as a set are toothed. Compared to most other species, the teeth of this species do much more (penetrative) work on chelal occlusion. *Glycometrus hugheseae* G3 (together with *Lepidoglyphus destructor* G6 and *Tyrophagus putrescentiae* T13) have the most ‘pocketed’ gullets (nadir angle $\le 93^{\circ }$). In *Glycometrus hugheseae* G3, intercalated gullets are not more pocketed than the angle of the distal gullet behind the moveable digit tip. *Glycometrus hugheseae* G3 shows morphological specialisation to grip, tear and chew small objects distally. Indeed, the $\gamma$ ‘claw’ angle is more acute in this species giving a more ‘pinched face’ to its gross Type A tearing/chewing design. The more acute proximal $\gamma$ ‘hook’ or ‘claw’ angle suggests a ‘nibbling’ specialisation here for this species. The mastication surface in this species has, compared to most other bird nest astigmatans, coarser and more rasping properties. As a hypothesis of a Gaussian surface is rejected for this species, there are not independent fluctuations along the reference axis *L*2*M* i.e., concerted adaptations into ‘modules’ are present. Could this be a ‘picker’ of relatively smaller objects that it then cuts/slices up?

#### *Glycyphagus domesticus* G5

The same specialisations as in previous studies (Bowman 2023a, 2024a, b, 2025b) were found. *Glycyphagus domesticus* G5 is highly derived distally. This species distally has a more saw-like nibbling Type B mastication surface design. Together with *Lepidoglyphus destructor* G6, *Glycyphagus domesticus* G5 showed markedly more teeth and gullets, the mastication surface clearly approximating a standard saw. Proximally this species’ tooth back angle is markedly polymorphic. The more acute proximal $\gamma$ ‘hook’ or ‘claw’ angle suggests a ‘nibbling’ specialisation here for this species. Proximally this species is the most ‘pocketed’. *Glycyphagus domesticus* G5 (with *Lepidoglyphus destructor* G6) have the sharpest teeth (zenith angle $\le 80^{\circ }$) over all the birds nest astigmatans, suggesting that its mastication surface acts like a grater, surform or coarse rasp suitable to attack chitin-like material. For gullet nadir angles, *Glycyphagus domesticus* G5 has high within individual variation. This species has particularly low values for gullet nadir angles suggesting adaptation for scratch digging and ‘hook and pull digging’ into foodstuffs. *Glycyphagus domesticus* G5 has a sharp proximal pocket that may function as a latch (see Bowman 2025b). The surface teeth as a set are saw-like. Indeed, *Glycyphagus domesticus* G5 is unusual in having repeated extra intercalated gullets. Amongst the multi-gulleted species, *Glycyphagus domesticus* G5 particularly shows evidence of alternating sharpening and blunting of intercalated gullets along the mastication surface. There is also some evidence of extra sharpening of intercalated gullets as more are intercalated. Even amongst the intercalated teeth, there is also a posterior trend for ‘latch-like’ pocketing. *Glycyphagus domesticus* G5 is morphologically specialised to grip, tear/chew and saw (triturate) large objects. Accordingly, *Glycyphagus domesticus* G5 has a distinct special micro-hooking form of mastication surface (like a hacksaw).

#### *Lepidoglyphus destructor* G6

*Lepidoglyphus destructor* G6 is highly derived distally. The surface teeth as a set are tenon saw-like. Together with *Glycyphagus domesticus* G5, *Lepidoglyphus destructor* G6 showed markedly more teeth and gullets, the mastication surface generally approximating a standard saw. *Lepidoglyphus destructor* G6 had a reduced scale of distal rake to its teeth (i.e., $-56^{\circ }$ compared to the typical $-70^{\circ }$). *Lepidoglyphus destructor* G6 (with *Glycyphagus domesticus* G5) have the sharpest teeth (zenith angle $\le 80^{\circ }$) over all the birds nest astigmatans, suggesting that its mastication surface acts like a grater, surform or coarse rasp suitable to attack chitin-like material. *Lepidoglyphus destructor* G6 (together with *Glycometrus hugheseae* G3 and *Tyrophagus putrescentiae* T13) have the most ‘pocketed’ gullets (nadir angle $\le 93^{\circ }$). *Lepidoglyphus destructor* G6 distally has a more saw-like nibbling Type B mastication surface design. The greater the number of intercalated teeth in *Lepidoglyphus destructor* G6, the noticeably sharper the teeth become. *Lepidoglyphus destructor* G6 (*Glycyphagus domesticus* G5 and possibly *Acarus farris* A17) show alternating tooth sharpening and blunting of intercalated teeth as one moves along the mastication surface. *Lepidoglyphus destructor* G6 is particularly unusual in also having many repeated extra intercalated gullets. Amongst the multi-gulleted species, *Lepidoglyphus destructor* G6 particularly shows evidence of alternating sharpening and blunting of intercalated gullets along the mastication surface as well. *Lepidoglyphus destructor* G6 (with *Glycometrus hugheseae* G3) has the sharpest zenith angle for its ATB-like teeth on average at $\approx 73^{\circ }$. Distally, there is a large initial rising first tooth in this species. Compared to most other species, the teeth of this species do much more (penetrative) work on chelal occlusion. The $\gamma$ ‘claw’ angle is more acute in this species giving a more ‘pinched face’ to its gross Type A tearing/chewing design. The more acute proximal $\gamma$ ‘hook’ or ‘claw’ angle suggests a ‘nibbling’ specialisation here for this species. The mastication surface in this species has, compared to most other bird nest astigmatans, coarser and more rasping properties. As a hypothesis of a Gaussian surface is rejected for this species, there are not independent fluctuations along the reference axis *L*2*M* i.e., concerted adaptations into ‘modules’ are present. The mastication surface in *Lepidoglyphus destructor* G6 is adapted to grip slippery foodstuffs so being a possible facultative nematode feeder? Consiliently, the moveable digit disproportionately thins on digit length increase (thus becoming more ‘cutting’ style adapted).

#### *Dermatophagoides pteronyssinus* D3

Along with *Suidasia pontifica* S5, *Dermatophagoides pteronyssinus* D3 has the greatest degree of chelal underbite - suitable for prising or levering up material from the substrate (dehiscing skin scales?). Indeed in having blunt asperity regions following blunt asperity regions this species is most bar-like in general form. Although distally, there is a large initial rising first tooth in this species. *Dermatophagoides pteronyssinus* D3 is highly derived distally and is particularly heterodontous. *Dermatophagoides pteronyssinus* D3 had a reduced scale of distal rake to its teeth (i.e., $-56^{\circ }$ compared to the typical $-70^{\circ }$). This species distally has a more saw-like nibbling Type B mastication surface design. The surface teeth as a set are strong blade-like. Intercalated teeth are rare in this species. This species shows the biggest difference between the proximal and distal regions (see the peak zenith and valley nadir angle data above). Proximally the profile is (uniquely) disproportionately rather feeble or frail (i.e., the opposite of robustified). The characteristics of the proximal gullet favours a (unique) grappling action. This species has noticeably flatter FT teeth with average zenith angles $\approx 115^{\circ }$ (more like scrapers). *Dermatophagoides pteronyssinus* D3 has a long proximal inter-peak and inter-valley distance ($\equiv$ enhanced Type B design here). The change in gullet depth as one moves posteriorly is variable in this species. *Dermatophagoides pteronyssinus* D3 usually showed a gross Type A hooking/tearing design adapted for protrusion into foodstuff. The $\gamma$ ‘claw’ angle is more acute in this species giving a more ‘pinched face’ to its gross Type A tearing/chewing design. Yet, *Dermatophagoides pteronyssinus* D3 shows a more ‘open faced’ nibbling design when present than in other species. *Dermatophagoides pteronyssinus* D3 teeth on average have the lowest penetration into foodstuff ($0.3\mu$m) and the moveable digit disproportionately thins on digit length increase (thus becoming more ‘cutting’ style adapted). *Dermatophagoides pteronyssinus* D3 in being distinct is to be expected as Pyroglyphidae although free-living "...feed mainly on the corneous material desquamating from the skin of their host..." and from a morphological standpoint are "... characteristics of the parasitic Psoroptidia.." of mammals (Fain et al. 1993). Perhaps they concentrate on blade-like slicing of scraped, gathered and manoeuvred food material?

#### *Suidasia pontifica* S5

Along with *Dermatophagoides pteronyssinus* D3, *Suidasia pontifica* S5 has the greatest degree of chelal underbite - suitable for prising or levering up material from the substrate. Although *Suidasia pontifica* S5 has the shortest distance from digit tip to the first gullet (in line with its small size, *IL*). Intercalated teeth are rare in this species. *Suidasia pontifica* S5 has a long proximal inter-peak and inter-valley distance ($\equiv$ enhanced Type B design). *Suidasia pontifica* S5 is unusual in having relatively blunt teeth. *Suidasia pontifica* S5 shows morphological specialisation to grip, tear and chew small objects distally. *Suidasia pontifica* S5 may have a special micro-hooking form of mastication surface. *Suidasia pontifica* S5 shows a slightly more ‘open faced’ nibbling design than in other species. *Suidasia pontifica* S5 (along with *Acarus farris* A17) has the smoothest fine-grinding action mastication surface (like a fret-saw). However, it may simply represent a small form mite - one that klinorhynchidly collects and gently squashes material with its moveable digit blades.

### Future work

Various other animals could be usefully compared to mite moveable digit designs e.g., placodonts (https://qilong.wordpress.com/2012/11/12/placodonts-are-also-cool/, ichthyosaurs (Huang et al. 2020), fossil crocodylomorphs (Ösi 2014, Muscioni et al. 2024), amphisbaenians and more fossil lizards (Bolet et al. 2014, Villa and Delfino 2019, Bochaton et al. 2016), even ancient marsupial-forms (Cácceres and Dickman 2023). Many of these exemplars are known durophages. The lizard *Pseudopus apodus* even has the distal to proximal within-individual variation of tooth zenith angle and variable tooth faceting (albeit via processes and striations) found in astigmatans (see Fig. 5 in Klembara et al. 2014). While some extinct kangaroos had jaws which were specialised for crushing (Mitchell 2019).

To what extent could the diversity of free-living bird nest astigmatans be mimicking the variety of feeding designs seen in the herbivorous and omnivorous artiodactyls (https://en.wikipedia.org/wiki/Artiodactyl)? For sure, examining the fixed digit for any complementary topology or unique adaptations is a clear opportunity for future work. Are there astigmatan analogues of reptilian archosaurs (https://en.wikipedia.org/wiki/Archosaur)? What about more comparisons to the jaws of extinct herbivorous sauropods (https://en.wikipedia.org/wiki/Sauropodomorpha), omnivorous theropods (https://en.wikipedia.org/wiki/Theropoda or ornithischians (https://en.wikipedia.org/wiki/Ornithischia)? Indeed, is there any evidence of an acarine beak-like function as in ceratopsids (see http://markwitton-com.blogspot.com/2015/02/controversial-ceratopsids-revisited.html)? Is there any evidence of paired upper (fixed digit) and lower (moveable digit) teeth modified in such a way as to allow enlarged and often self-sharpening edges to pass by each other in a shearing manner like vertebrate carnassials (https://en.wikipedia.org/wiki/Carnassial)? A SEM follow-up study is needed.

Could the ‘latch’ mechanism described for several bird nest astigmatan species actually be an adaptation to trap material of just the right diameter as the proximal gullet and then not just hold it and strip it of material but then to ‘snap’ it off from the substrate? The bite force at this proximal area will be the highest a mite can deliver given its particular cheliceral and chelal design. Better measurement, given the maximum gape of the chela, of the likely diameter of this ‘socket’ between the moveable and fixed digit surfaces would help match this to candidate fibrous foodstuffs.

Grinding material (like various types of metal files do) requires knowing the characteristics of the substrate well. Bremer and Matthiesen (2020) points out that, besides "... general classifications, the mechanical properties of foods are complex to describe and depend on the production processes, for example on the maturation process of cheese..." material. Hubert and Mourek (2002) reviews the variety of interactions between astigmatans and fungi. So a better characterisation of potential trophic organic matter found in bird nests is needed. Indeed as Bremer and Matthiesen (2020) goes on "... In addition to similar dependencies on chemical properties like pH, meat products usually have structural influences, examples being, fibrous structures in muscle tissues or the composition of fat and muscle tissues...". So, intensive sampling and mechanical (for example rigidity and penetrative resilience) characterisation of potentially nutritive nidicolous material for astigmatans is needed. Feeding mites deliberately on different durophagous diets and examining micro-wear patterns (as in fishes Purnell and Darras 2016 and in pterosaurs Bestwick et al. 2020) on the chitin of their digit teeth compared to wild-collected samples may help understand astigmatan ecomorphology more.

More detailed investigations of the mite ‘saw-like’ designs could be made comparing them to known literature (see http://www.backsaw.net/index.php/2-uncategorised/4-saw-literature-on-line) using other characterisations of the bird nest astigmatan moveable digit surfaces. For instance *m* (the ‘drape’ or ‘chain’ distance) or the tooth-row scaled *rel* measure from Bowman (2024a) and comparisons made to bee-hive astigmatans and other zoological surfaces like coral. However, these are functions of vertical fluctuations integrated over asperity spacing (albeit over a proportion of *L*2*M*), so they may be essentially already captured by the other characteristics used herein.

An agreed functional classification scheme for arthropod jaws needs to be developed much like the eight categories used for claws (Thomson and Motani 2021). Within such, then for at least grasping and cutting/slicing, more follow-up work on acarine chelae is needed (Bowman 2024b). Since, for sure as Joseph Roland, The Amateur of Fencing, said in 1809:

"...That there are persons of mistaken ideas in almost every Art or Science, is what few will deny. Yet I am inclined to believe there are more erroneous opinions entertained with regard to the Art of using the Sword than on most other subjects..."

Indeed just like a fencer advances as well as strikes, then concomitant gnathosomal or cheliceral movements (e.g., chelal ‘waggling’) will also affect the physics involved. Sabre-tooth cat bite mechanics is a function of tooth, jaw, neck, head and forelimb design (Wroe et al. 2013, Brown 2014). Exactly, how a free-living astigmatan mite feeds airorhynchidly or klinorhynchidly matters. More detailed live observations are needed.

Furthermore, as Owen Seeman (*pers.comm.*) has pointed out, whether chelicerae work in tandem or not "...alters thoughts about trophic function...". Extending this thought for instance that "...If somebody were to tell me to eat a tough old steak with a pair of identical tools, what would be best? I doubt that I would use each identically, but would use one to hold and the other to saw...". So perhaps the chelicerae combine to work like a ‘knork’? He suspects "...that at least dual roles have to be fulfilled by most chelicerae - except species with stylets that merely stab-and-suck (i.e. parasites)....".

That is not to say, that even more unusual mechanisms may actually come into play with mite chelae. For instance could the bird nest astigmatan digits be deploying an additional substance to abrade at foodstuff much like the silicon carbide crystal pastes applied to the wire bow-saw surface used in ‘pietra dura’ cutting (https://www.greenawaymosaics.com/pietra-dura)? Could viscous semi-granular salt-like material be produced by the enigmatic supra-coxal glands of astigmatans (see Miller 2019) and be channeled into the gnathosoma? The supracoxal setae acting like the tritosternum of mesostigmatids (Bowman 2023b)? Such lubrication or special surface coating properties could be "... influencing the frictional contact between slice and blade surface..." as in food processing (Bremer and Matthiesen 2020).

Whether the moveable digit design Type A is plesiomorphic or design Type B is the least derived needs investigation. If astigmatans followed one of the proposed paths for scarabaeid beetle mouthpart evolution (Bai et al. 2015), then Type A suitable for micro-coprophagy would be the more derived form. Indeed is there any evidence from gut contents that *Acarus gracilis* A4, *Chortoglyphus arcuatus* CH1 or *Dermatophagoides pteronyssinus* D3 are moist material feeding coprophiles in bird nests?

Other biologically distinct species (listed by Hughes 1976) could be used as an out-group test, e.g., *Cosmoglyphus oudemansi* C10, *Lardoglyphus konoi* L1, *Lardoglyphus zacheri* L3 (typical of dead bodies), *Sancassania berlesei* C3 (common in wet cadavers) and *Tyroborus lini* T66, as well as some other taxa which also encompass from feathers (like other *Dermatophagoides spp.* do) and fruit (e.g., *Carpoglyphus lactis*, Ca4). The moveable digit profiles of more *Acarus* spp., *Rhizoglyphus* spp., *Thyreophagus* spp. and *Thyreophagus* spp. from other habitats could be usefully assayed as taxonomically matched out-groups. Finally, less well known acarids *Cosmoglyphus hugheseae* (C5), *Forcellinia galleriella* (F1) and *Kuzinia laevis* (KL), or even less related taxa like the suidasid *Neosuidasia* sp. (LA1) or the winterschmidtidiid (CV1) from Bowman (2021) could be investigated as counterfactuals.

Some marsupials show durophagous strengthening adaptations to their anterior i.e., their distal teeth (Churchill et al. 2022) whilst retaining slicing posterior dentition. Are there similar mite examples? Indeed, modelling the variation in the depth and thickness of digit dentition so as to better understand the actual stresses and strains upon them given the postulated functions is needed (see Blanco et al. 2010 for some simple morphological methods). Is there any evidence of strain-induced assembly (like in bone Erhlich and Lanyon 2002) in the form of the over-reinforced chitinous moveable digit tips of *Acarus gracilis* A4, *Glycyphagus domesticus* G5 and *Lepidoglyphus destructor* G6 (Bowman 2025b)? A start on investigating this perhaps could be first done by estimating the overall moveable digit 3D shape from the observed shading (Snape 2016) in follow-up scanning electron micrographs before attempting (micro-) computerised tomography usually needed for finite element analysis in animals (e.g., Qin et al. 2023). This might also allow the development of a useful simple scoring scheme for gullet shape (like the ATB versus FT descriptor for teeth) for taxonomic acarologists to use.

What might be the situation for male bird nest astigmatans? Sexual dimorphism is common in arachnids (McLean et al. 2018). Sex-differentiating shape variations were found along the first principal component of size-normalised statistical shape models (particularly in the symphysis and posterior ramus regions) of human mandibles Vallabh et al. (2020). An ontogenetic investigation (e.g., Estes and Williams 1984, Frederich et al. 2008) would be useful to see if the various different biting capacities are maintained in juvenile stages of each species. In particular, what may be very interesting would be to see if durophagy in juvenile mites is associated with an even bigger degree of robustification since they should have a lower absolute adductive input force *F*1 (see argument in Herrel and Holanova 2008).

The temporal and spatial variation already recognised as major factors in explaining morphological changes in the mammalian skull at both micro- and macroevolutionary scale (Crampton et al. 2024) must play a part in bird nest astigmatans. For sure, bird nests show phased colonisation by mites (Fain et al. 1993). The occurrence of apparent within-taxon variation in astigmatid dentition offers the opportunity to try to isolate genetic mutants of such (as done in the aetiology of zebrafish craniofacial morphology Parsons et al. 2011). Developmental variants are found in mammal mandibles (Jentzsch et al. 2020). Once similar is found in mites, experimental manipulations could be made and complex patterning (Levin 2012) dissected. The recent discovery of 6-phospho-gluconate dehydrogenase polymorphisms directly linked to whole animal phenotypes in *Rhizoglyphus robini* (Unnikrishnan et al. 2024) offers optimism that this might be achieved.

The segmentation discovered in the morphology of the laboratory-based set of individuals for each species needs to be replicated by wild-collected specimens and validated by feeding studies. For sure, "... behavioural flexibility...." in "... the absence of a clear one to one relationship between structures and functions...." (Purnell and Darras 2016) may occur. How prevalent astigmatan infestation of bird nests is, needs to be better elucidated - one easily accessible model system might be to survey artificial sand-martin nest bank ‘hotels’ used in restorative bio-conservation (e.g., https://www.bbc.co.uk/news/uk-england-nottinghamshire-26498229).

Surprising trophic abilities can be discovered in unlikely animals, e.g., the recent "...report of a wolf — a large, terrestrial-bound mammal and otherwise strict carnivore — as a nectar feeder and potential pollinator..." Lai et al. (2024), Detailed fieldwork on the mites’ actual diet could convert their morphological functional groups into guilds (Denzinger and Schnitzler 2013) and resolve multiple distinct morphologies (as in bats, Hedrick and Dumont 2018) for the same apparent niche. Niche partitioning by character displacement (Begon and Mortimer 1981) when in co-existent competition within a habitat is expected (much as the ecomorphological correlates of anole lizard claw shape changes, e.g., Yuan et al. 2020). Can this be found in free-living bird nest astigmatan communities? Could any ‘character release’ (Begon and Mortimer 1981) be detected in disturbed environments? Is it possible to relate particular free-living astigmatan designs to the commensal opportunity of scavengeable food material produced by other particular co-existent animals in the bird nest community (much as posed for early hominids accessing carcasses left over from carnivore attack, Harstone-Rose 2008)?

Finally formal phylogenetic investigations as to the eco-morphological drivers of trophic size and shape in astigmatans could be carried out such as like in toothed whales Vicari et al. (2023) or carnivorous mammals Goswami et al. (2011). However, just as in fossorial rodents (Rodrigues et al. 2023) multiple modifications of the different components of the astigmatan masticatory apparatus may have led to similar overall morphologies and functions, overcoming phylogenetic inheritance.

## Overall conclusion

The basic design of a bird nest astigmatan moveable digit approximates a multi-purpose metal weapon called a glaive (https://en.wikipedia.org/wiki/Glaive which can be used like a spear, a sickle, a serrated knife, an axe and a sword (Fig. 29). Just as in vertebrates (Gálvez-López and Cox 2022), highly similar astigmatan species can have substantial biomechanical differences pertinent to feeding.

The early 20th century arms collector and fencing historian Sir Frederick Pollock wrote in 1911:

"... A good sword should be elastic, so as to stand bending or a heavy blow without breaking or permanent deformation, and yet stiff enough to deliver a powerful thrust without yielding too readily from the straight: it must also be as light as is possible consistently with strength, and well balanced. All four desiderata are met in the main by the use of a suitable steel, properly treated and disposed, but balance is also dependent on the weight and form of the hilt. As regards the effect of disposition, grooving or ‘fullering’ the flats of the blade reduces weight without impairing strength, and is now very largely adopted...".

The (also ‘gripping-style’) moveable digits of bird nest astigmatans are similarly well designed for their different scratching, piercing, hooking, grinding, slice cutting and crushing functions within the cheliceral chela. These astigmatan designs may lead to new biomimetic tools for particular macro-scale usage. However, like with soft robot fingers (Cutkosky and Wright 1986) how friction arises when hard chitinous asperities grasp compliant materials, like mite foodstuffs, remains to be be investigated.

Bar *Tyrophagus palmarum* and *Tyrophagus similis*, morphological adaptations were found in free-living bird nest astigmatans to facilitate their co-existence (via ecological partition into different trophic niches) for all the other surface omnivores, surface fragmentary feeders, interstitial omnivores and interstitial fragmentary feeders studied.

## Figures and Tables

**Fig. 30:**
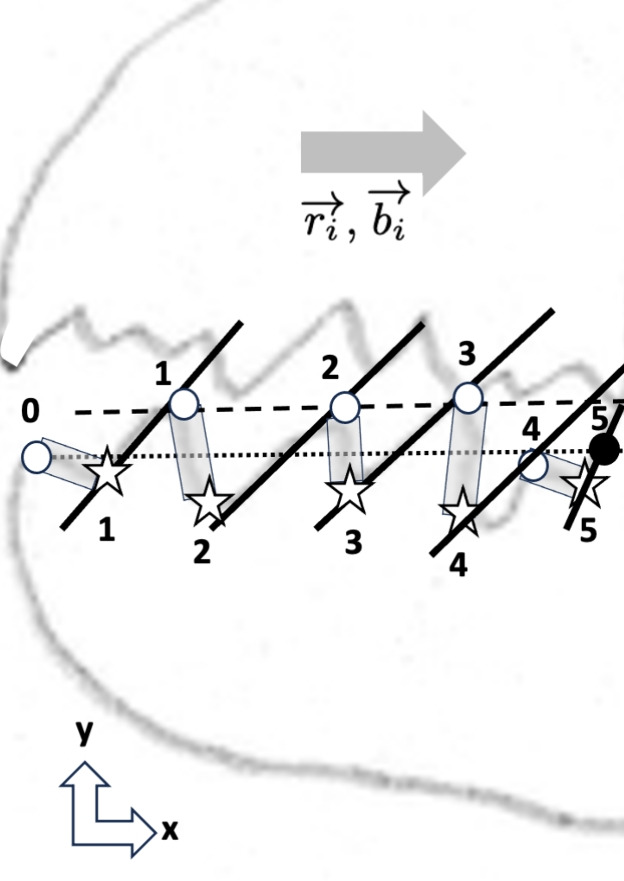
Rake (grey cutting surfaces) and back angle (solid lines) for peaks (dots) and gullets (stars) at moveable digit profile [*x*, *y*] numbered locations in *Glycyphagus domesticus* from Bowman (2024a) (amended with permission, Creative Commons Attribution 4.0 International License http://creativecommons.org/licenses/by/4.0/). Chelicera pulled posteriorly (large grey arrow). Black dot is at end of mastication surface ($x_{i_{e}}$) along reference *L*2*M* axis (tip to condyle). Here there are $(0,1,2,3,4,5)=6$ peaks and 5 gullets. The last peak only has a back angle (where *W* is measured)

**Fig. 31:**
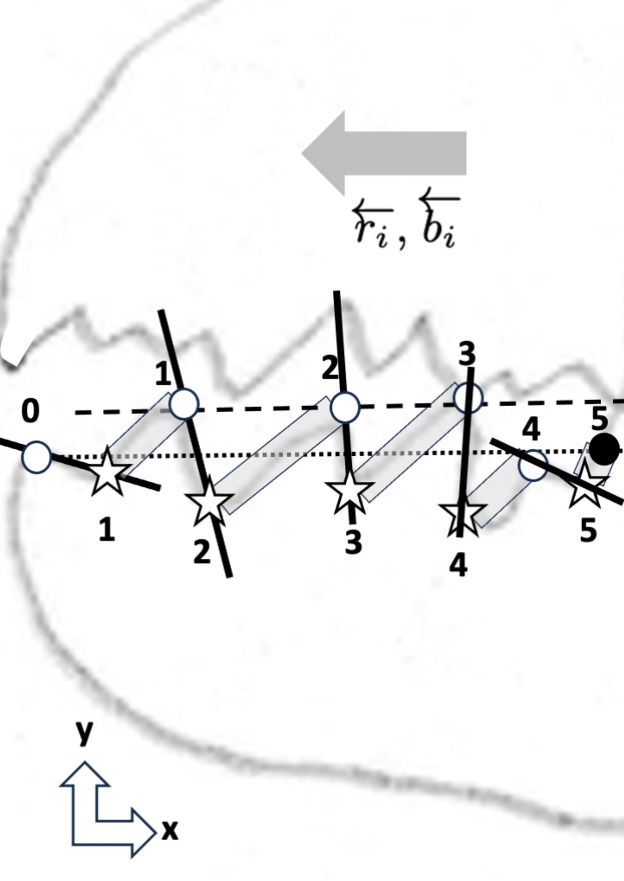
Rake (grey cutting surfaces) and back angle (solid lines) for peaks (dots) and gullets (stars) at moveable digit profile [*x*, *y*] numbered locations in *Glycyphagus domesticus* from Bowman (2024a) (amended with permission, Creative Commons Attribution 4.0 International License http://creativecommons.org/licenses/by/4.0/). Chelicera pushed anteriorly (large grey arrow). Black dot is at end of mastication surface ($x_{i_{e}}$) along reference *L*2*M* axis (tip to condyle). Here there are $(0,1,2,3,4,5)=6$ peaks and 5 gullets. The last peak only has a back angle (where *W* is measured)

**Fig. 32:**
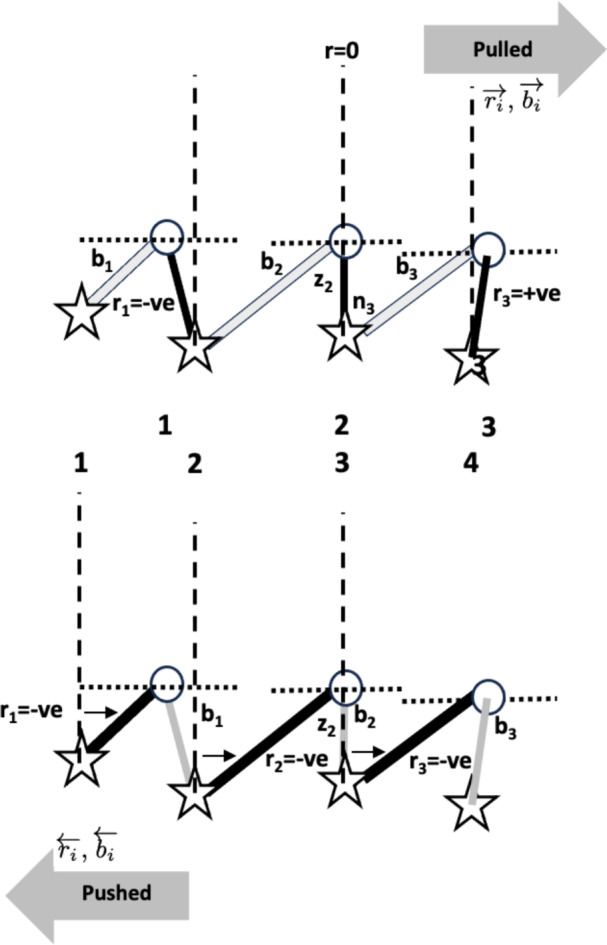
Diagram how angle of mastication surface asperities are related. Upper: digit pulled rightwards into foodstuff (matches Fig. 30). Lower: digit pushed leftwards through foodstuff (matches Fig 31). Dotted lines are parallel to reference *L*2*M* axis for back angle (*b*) measurement. Central figures indicate teeth and gullet numbering as in Figs. 30, 31. Vertical dashed lines are reference basis for rake angle (*r*) measurement. Cutting face of tooth in black, trailing facet of tooth in grey. *z* is an example second tooth zenith angle, *n* is an example third gullet nadir angle. Back angles are always positive, the sign of the rake angle changes with the direction of sawing in this example as well as the teeth design

## Data Availability

All new data generated or analysed, plus any model specifications are included in this published article, or in compliance with EPSRC’s open access initiative are available from https://ora.ox.ac.uk/objects/uuid:c4132662-f53a-4dee-a342-c94f202b0e8f

## Technical Appendix

### Push versus pull

In Fig. 30, let open dots denote the location of peaks, and open stars the location of gullets along the astigmatan moveable digit mastication surface. Let the moveable digit tip be the zero-th peak, and the position of $x_{i_{e}}$ on the *L*2*M* axis be the last ‘peak’ (here highlighted in black) where *W* is assessed.

Then for the digit tip being at $x=0,y=0$, for the moveable digit being **pulled posteriorly**, the cutting surface of a rising asperity (or numbered tooth) has a

$$rake_{peak(i)}=tan^{-1}\left[ \frac{y_{gullet(I+1)}-y_{peak(i)}}{x_{gullet(i+1)}-x_{peak(i)}}\right] -\frac{\varPi }{2}$$

in radians. This is equivalent to

$$-tan^{-1}\left[ \frac{x_{gullet(i+1)}-x_{peak(i)}}{y_{gullet(I+1)}-y_{peak(i)}}\right]$$

Also then for the digit tip being at $x=0,y=0$, for the moveable digit being **pulled posteriorly**, the trailing surface of a rising asperity (or numbered tooth) has a

$$back\_angle_{peak(i)}=tan^{-1}\left[ \frac{y_{peak(i)}-y_{gullet(i)}}{x_{peak(i)}-x_{gullet(i)}}\right]$$

in radians. This tooth action (counter-intuitively) matches the action of a conventional ‘Western-style’ push saw.

Another way to look at this is to consider any $\left[ \frac{\varDelta (y)}{\varDelta (x)}\right]$ terms in these definitions as ‘slopes’ along an [*x*, *y*] profile with increasing *x* from the tip origin (like the $g_{i}$ values in Bowman 2024a). Then (for the chela pulled posteriorly) if the slope is expected to be negative due to the presence of a topological decline in the asperity (i.e., $\varDelta (y)<0$) this maps to a *rake* given by $-tan^{-1}(\frac{1}{slope})$, or if the slope is expected to be positive due to the presence of a topological rise in the asperity (i.e., $\varDelta (y)>0$) then this maps to a $back\_angle$ given by $tan^{-1}(slope)$. Recall that back angle is positive but rake can be 0, negative or positive (the latter as in recurved ripping saw teeth).

Then, the tooth zenith angle of the *i*th peak $=\frac{\varPi }{2}-rake_{peak(i)}-back\_angle_{peak(i)}$ ($modulo\ \varPi$) and, the gullet nadir angle of the $(i-1)$th gullet $=\frac{\varPi }{2}-rake_{peak(i-1)}-back\_angle_{peak(i)}$ ($modulo\ \varPi$). Note that $b_{last}$ is the back angle at *W* i.e., adjusted by the last gullet depth.

If the moveable digit is **pushed anteriorly** (into food) then this of course has no effect on zenith and nadir angles but cutting surfaces now become trailing surfaces and vice versa for each tooth (Fig. 31). Then the ‘slope-based’ rule becomes if $\varDelta (y)>0$ this becomes a rake and if $\varDelta (y)<0$ this becomes a back angle. This tooth action (counter-intuitively) matches the use of a Japanese pull saw employed for precise cuts in multiple types of materials.

Changing notation is useful here to summarise relationships.

For the chelicera pulled posteriorly (Fig. 30) let

$$rake_{peak(i)} \Rightarrow \overrightarrow{r_{i}}$$

$$back\_angle_{peak(i)} \Rightarrow \overrightarrow{b_{i}}$$

and for the chelicera pulled anteriorly (Fig. 31) let

$$rake_{peak(i)} \Rightarrow \overleftarrow{r_{i}}$$

$$back\_angle_{peak(i)} \Rightarrow \overleftarrow{b_{i}}$$

The arrows above the parameter indicating the direction (left$$\rightarrow$$right Fig. 30; right$$\rightarrow$$left Fig. 31) on the printed page and *i* being the peak counter from the digit tip (at $$x=0$$) to the end of the mastication surface ($$x_{i_{e}})$$ Then for angles in radians, by simple trigonometry

$$\overleftarrow{r_{i}}=-\left( \frac{\varPi }{2}-\overrightarrow{b_{i}}\right)$$

$$\overleftarrow{b_{i}}=\frac{\varPi }{2}+\overrightarrow{r_{i}}$$

Furthermore, the tooth zenith angle of the *i*th peak in radians is

$$\varPi -\overleftarrow{b_{i}}-\overrightarrow{b_{i}}$$

and, the gullet nadir angle of the *i*th gullet in radians is

$$-(\overrightarrow{r_{i-1}}+\overleftarrow{r_{i}})$$

This is all illustrated in Fig. 32. Note this Figure has the chelicera pushed into food towards the right (in the upper subfigure, equivalent to Fig. 30) and pulled through food towards the left (in the lower subfigure, equivalent to Fig. 31) and the saw cutting/trailing edges swopped in colours. For the middle tooth $\overrightarrow{r}=0$ and correspondingly $\overleftarrow{b}=\frac{\pi }{2}$ radians. A different lower figure with $\overleftarrow{r}>0$ (and resultant small $\overleftarrow{b}$) could be drafted.

Note that from simple trigonometry, circular functions combining these angles (along with the *L*2*M* scale) will determine moveable digit inter-peak distances, inter-gullet distances, rising totals (*rRt*(*i*)) and falling totals (*fRt*(*i*). The latter measures may be easier to interpret functionally but all are aliased with the set of zenith and nadir angles for an individual. They are as fundamental as the triangular basis of geometric morphometrics in understanding deformation (i.e., slope changes of a profile).
